# Identifying the women most vulnerable to intimate partner violence: A decision tree analysis from 48 low and middle-income countries

**DOI:** 10.1016/j.eclinm.2021.101214

**Published:** 2021-12-02

**Authors:** Carolina V N Coll, Thiago M Santos, Karen Devries, Felicia Knaul, Flavia Bustreo, Anne Gatuguta, Gbenankpon Mathias Houvessou, Aluísio J D Barros

**Affiliations:** a International Center for Equity in Health, Federal University of Pelotas, Pelotas, RS, Brazil; b Postgraduate Program in Epidemiology, Federal University of Pelotas, Pelotas, RS, Brazil; c London School of Hygiene and Tropical Medicine, 15-17 Tavistock Place, London WC1H 9SH, United Kingdom; d Institute for Advanced Study of the Americas, University of Miami, Coral Gables, FL 33146, United States; e Fondation Botnar, Geneva, Switzerland; f Department of Global Health and Infection, Brighton and Sussex Medical School, University of Sussex, Brighton, United Kingdom

**Keywords:** Violence against women, Intimate partner violence, Low and middle income countries, Interventions

## Abstract

**Background:**

Primary prevention strategies are needed to reduce high rates of intimate partner violence (IPV) in low- and middle-income countries (LMICs). The effectiveness of population-based approaches may be improved by adding initiatives targeted at the most vulnerable groups and tailored to context-specificities.

**Methods:**

We applied a decision-tree approach to identify subgroups of women at higher risk of IPV in 48 LMICs and in all countries combined. Data from the most recent Demographic and Health Survey carried out between 2010 and 2019 with available information on IPV and sociodemographic indicators was used. To create the trees, we selected 15 recognized risk factors for IPV in the literature which had a potential for targeting interventions. Exposure to IPV was defined as having experienced physical and/or sexual IPV in the past 12 months.

**Findings:**

In the pooled decision tree, witnessing IPV during childhood, a low or medium empowerment level and alcohol use by the partner were the strongest markers of IPV vulnerability. IPV prevalence amongst the most vulnerable women was 43% compared to 21% in the overall sample. This high-risk group included women who witnessed IPV during childhood and had lower empowerment levels. These were 12% of the population and 1 in 4 women who experienced IPV in the selected LMICs. Across the individual national trees, subnational regions emerged as the most frequent markers of IPV occurrence.

**Interpretation:**

Starting with well-known predictors of IPV, the decision-tree approach provides important insights about subpopulations of women where IPV prevalence is high. This information can help designing targeted interventions. For a large proportion of women who experienced IPV, however, no particular risk factors were identified, emphasizing the need for population wide approaches conducted in parallel, including changing social norms, strengthening laws and policies supporting gender equality and women´s rights as well as guaranteeing women´s access to justice systems and comprehensive health services.

**Funding:**

Bill and Melinda Gates Foundation (Grant INV-010051/OPP1199234), 10.13039/100010269Wellcome Trust (Grant Number: 101815/Z/13/Z) and Associação Brasileira de Saúde Coletiva (ABRASCO).

**Research in context**

**Evidence before this study**

The quantity and scope of IPV prevention interventions in low and middle-income countries (LMICs) has increased recently and many innovative programmes were shown to have impact in reducing IPV within programmatic timeframes. To maximize the effectiveness of promising interventions and ensure the best use of scarce resources in LMICs, efforts should tackle context relevant drivers that place women at higher risk of IPV. We used the search terms (“intimate partner violence” AND “risk factors” OR “vulnerable groups” AND “low and middle-income countries”). Globally a few multi-country studies to identify women at increased risk for IPV in the context of LMICs were found, but none applied a decision tree approach to identify the most vulnerable women.

**Added value of this study**

The identification of population targets is an essential step to guide informed decision-making and ensure that interventions and public health programming are effective in achieving sustained population level reductions of IPV. We used a decision-tree approach to identify the women most vulnerable to IPV and context-specific markers of IPV risk across 48 LMICs. A small subset of indicators identified groups of women with high levels of IPV and shared risk factors around which interventions could be focused.

**Implications of all the available evidence**

The evidence generated in the current study has the potential to guide stakeholders from multiple sectors in tailoring programs and interventions around locally relevant drivers of IPV. A large proportion of women who experienced IPV, however, had no indicators that could help with either targeting or tailoring interventions indicating the need for increased population awareness about the pervasiveness of IPV as well as continued improvements in legislation and access to health and protective services.

Alt-text: Unlabelled box

## 1 Introduction

Gender-based violence is ubiquitous and has severe consequences for women, their children, communities and for social and economic development [1], [2], [3]. Intimate partner violence (IPV) is the most common form of violence against women worldwide [4] and is considered a major obstacle to the fulfilment of human rights and the achievement of the Sustainable Development Agenda [5]. In response, there has been increased investment in intervention development and evaluation to reduce IPV and mitigate its consequences [6], [7], [8], [9]. A considerable number of interventions to prevent IPV have now been tested in randomised controlled trials with some achieving large effects in programmatic timeframes [10]. Some of the most effective examples include cash transfers programmes, combined economic empowerment and social empowerment interventions for women, participatory group-based approaches delivered to couples, parenting programmes and interventions that work with individuals and/or couples to reduce alcohol and substance abuse [11]. Similarly, community activism to transform social norms that support violence and promote non-violent, gender equitable behaviour have been highly successful [11].

But how can we get closer to population-wide reductions in IPV? A woman's risk of experiencing IPV is not uniform, and depends on the country she lives in [1,4], her social environment [7], her own characteristics and experiences and those of partners. [7,12,13] and there are likely combination of factors operating at multiple levels of the social-ecological model that may converge to increase the individual risk of IPV under different circumstances [12]. While there is growing consensus around the critical elements required for effective prevention, evaluations that demostrate significant and sustained changes in IPV levels still relatively scarce [10,14]. To advance the field of violence prevention and maximise the impact of promising interventions, the identification of context specific population targets as well as expansion in the range of drivers tackled is deemed necessary.

In this paper we use a decision tree approach in order to identify groups of women across 48 LMICs that are at the highest risk of IPV in all countries pooled together and in each country individually [15]. Decision trees are a robust statistical tool that can take into account the complex set of factors associated with IPV, identifying the most relevant intersections between those factors and relevant subgroups of women in the sample [16]. This analysis therefore builds on our earlier work which performed data stratification by a limited set of pre-specified sociodemographic indicators to identify subgroups of women at increased risk of IPV and estimate inequalities [1]. Creating decision trees for IPV can be thought of as a process of identifying smaller and somewhat homogeneous groups of women with a higher prevalence of IPV who could be specifically targeted for interventions and whose underlaying characteristics could provide key insights on how to tailor interventions. Using this approach, we aim to contribute evidence to optimize IPV prevention program design across the different LMICs and globally, supporting governments towards reducing population levels of IPV and reach the most vulnerable women considering their particularities.

## 2 Methods

For this analysis, we used data from Demographic and Health Surveys (DHS http://dhsprogram.com) because they include a standardized module on violence against women and cover a large number of LMICs. We selected the most recent survey carried out between 2010 and 2019 for which the data had been made publicly available. We restricted the time period to avoid surveys that are more than a decade apart in the pooled analysis and to ensure comparable questionnaires and methodologies. We identified surveys for 48 countries spanning 7 world regions, and representing 68% of all low-income, 37% of lower-middle and 16% of upper-middle countries, according to the World Bank classification for the median survey year (2015). The countries are listed in supplementary Table S1. All women aged 15–49 years who were usual residents of the selected households or had slept in the household the night before the interview were eligible for the survey.

### 2.1 How IPV was defined

DHS collect data on IPV using a standard module and methodology which follows the World Health Organization ethical guidelines. The questionnaire is applied to one randomly selected eligible woman per household. In all countries, interviewers were instructed to discontinue the interview if privacy could not be assured.

The IPV questions are based on a modified version of the Conflict Tactics Scale [17] which asks women whether their current or most recent husband/partner (if divorced, separated, or widowed) perpetrated a series of specific acts of physical, sexual, or emotional violence. In the current study, exposure to IPV was defined as having experienced at least one act of physical and/or sexual IPV by a current or former partner in the 12 months preceding the interview, regardless of frequency. Acts of physical IPV included pushing, shaking, throwing things, slapping, arm twisting, pulling hair, punching with the fist or something else that can hurt, kicking, dragging, strangling, burning, and threatening and/or attacking with a knife, gun, or other type of weapon. Sexual IPV was defined as having experienced one of the following acts: forcing sexual intercourse or any other sexual act when the respondent did not want to through the use of physical force or threats or any other way.

### 2.2 IPV risk factors

Variables were selected according to the criteria: i. being a well-established risk factors for IPV in the literature; [8,13] ii. being available and comparable in DHS surveys; iii. having a potential for tailoring and targeting of interventions. The following 12 risk factors (or proxies) were used for identifying women at increased risk of physical and/or sexual IPV: woman's age, number of living children, witnessed IPV in childhood (father-to-mother), woman´s empowerment level, woman´s and partner´s education (classified as no education, primary, secondary or higher), partner´s occupation, partner´s current alcohol use (yes/no), polygyny (number of partner´s co-wives), religious affiliation (categorised as Christian, Muslim or other), place of residence (urban/rural) and household wealth quintiles.

Woman's empowerment was assessed through the Survey-based Women´s emPowERment Global index (SWPER Global) [18]. The index is based on 14 items, allowing the assessment of three empowerment domains: i. attitude to violence, which comprises items related to the women's opinion on whether beating the wife is justified in some situations; ii. social independence, which includes access to information, educational attainment, age at first marriage and first child, and difference in age and education between the woman and her partner; and iii. decision making, which comprises three questions on who makes decisions in regard to the woman's health care, to major expenses, and to visits to family or relatives. Women were categorised into low, medium and high empowerment level based on their SWPER scores in each domain [18].

Partner occupation was classified into five categories: sales and services (clerical, household and domestic services), agricultural activities (self-employed or employee), manual labour (skilled or unskilled), professional/technical/managerial, and not at work.

Households were classified into wealth quintiles based on a wealth index calculated through principal components analysis using household assets, building characteristics of the dwelling and access to utilities (such as electricity and water mains) [19]. The wealth score was adjusted for urban or rural residence using a regression-based scaling procedure [20].

Subnational regions were included in country-level analyses and followed the classification of each country report using level one administrative areas. Supplementary Table S2 presents a list of all indicators and their availability amongst the selected surveys.

### 2.3 Statistical analysis

We employed a decision tree approach to find specific groups of at-risk women. The most commonly used method for creating decision trees is the Classification and Regression Tree (CART) [15]. Other methods are available, and the Conditional Inference tree (CTree) technique has been proposed as a valuable alternative to CART in epidemiological research. However, CTree has been shown to divide the sample in an overly large number of subgroups when the sample size is large – since it is based on a statistical hypothesis testing framework – rendering its interpretation impractical [16]. Due to the size of our sample and CART's stability when dealing with larger sample sizes, we chose CART for creating the decision trees.

In CART, the classification algorithm performs a binary recursive partitioning process. This means that, using the IPV risk factors, it will start by partitioning the complete sample of women into two groups (therefore binary): one with higher and other with lower prevalence of IPV. Those groups will then be split into ever smaller groups (therefore recursive) until a stopping rule is triggered. The stopping rule can be, for example, a minimal number of women in a group. In the end, each woman will be assigned to only one subgroup.

For the partition, the algorithm will explore all possible indicators (such as age or religion) and possible cut-off points (such as being under or above X years of age or belonging or not to a specific religion). The indicator and cut-off point that creates the two most homogenous groups (within each group) will be used as criteria for partitioning. In our case, a completely homogenous group would contain only women who experienced IPV (100% prevalence) or only women who did not experienced it (0% prevalence). For the intermediate cases, the level of homogeneity was measured using the Gini index, the most commonly used measure for deciding the partition criteria [21].

Decision trees are useful for identifying high-risk groups because they deal naturally with complex intersections between continuous and categorical indicators. This can help to investigate the intersections of social and demographic determinants of IPV, without relying on previous specifications by the investigators [21]. Ideally, the algorithm will create groups with high and low levels of IPV, depending on the availability of good enough predictors. Those indicators should be interpreted as descriptive markers that help to identify women within groups with a somewhat similar IPV profile.

We used CART to create trees for each country and for all countries combined. For most countries, the proportion of women who reported recent IPV was well below 50%, creating an unbalanced sample. When a decision tree is created, the algorithm performs a classification of each woman, determining if she is likely to have experienced IPV or not, based on which terminal group, also called node, she is assigned. If the prevalence of IPV is low, this is similar to a diagnostics tool used for a rare disease: the tree can simply classify every woman as “not having experienced IPV” and have high accuracy, but 0% sensitivity. To increase the tree's sensitivity, we adjusted the cost of misclassifying a woman who experienced IPV as not having experienced IPV (false negative) as double the cost of a false positive. This reduced the IPV prevalence threshold that is necessary in a node for the women in it to be classified as having experienced IPV, increasing its sensitivity while reducing its specificity. This double cost been used previously in CART in order to take into account a similarly large and multicountry unbalanced sample [22]. A more detailed description of how those costs are used in CART can be found elsewhere [23]. Any split leading to a node with less than 50 women was discarded.

R 4.0.2 and the rpart package version 4.1–15 were used for the analyses. Besides the misclassification cost and the minimum number of women in a node, we used all the standard parameters in the rpart package. Those parameters, as well as a sensitivity analysis exploring the effect of changing some of them in the resulting pooled tree are presented in the Supplementary Materials. All the analyses used sampling weights to adjust for the sample design. For the pooled analysis, weights were recalculated to take into account the population size of women aged 15–49 years in each country.

We estimated IPV prevalence and respective confidence intervals using Stata (StataCorp. 2019. Stata Statistical Software: Release 16. College Station, TX: StataCorp LLC.) and created a world map to present them using R and publicly available map datasets from Natural Earth (https://github.com/nvkelso/natural-earth-vector). We also created country profiles including the national decision tree alongside with key indicators related to IPV. We selected Kenya as a case study and further created a country map with IPV prevalence displayed at the province level. This choice for Kenya was because the national tree features subnational regions as the main IPV risk marker and because it is the country of origin of an author of this paper who was able to further contextualize the findings.

All analyses relied on publicly available, anonymized databases for which permission to access was received through the DHS Program. The organisations who administered the surveys were responsible for ethical clearance according to the norms of each country.

### 2.4 Role of the funding source

The funder of the study had no role in study design, data collection, data analysis, data interpretation, or writing of the report. The corresponding author had full access to all the data and had final responsibility for the decision to submit for publication.

## 3 Results

### 3.1 Intimate partner violence prevalence

**Fig. 1:**
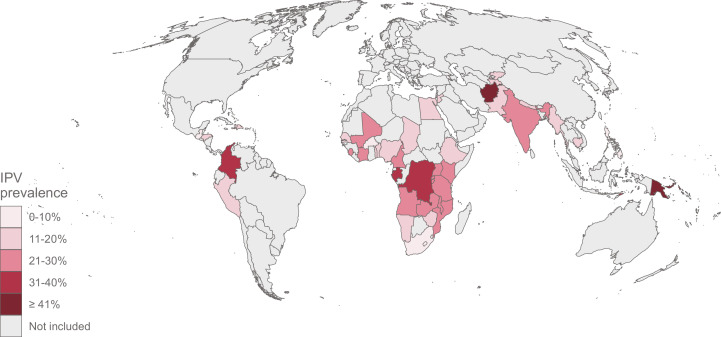
Intimate partner violence prevalence map and top 5 countries with highest levels. The colour was used to indicate the IPV prevalence in each country. Countries in grey were not included in the analysis. Countries in lighter pink have lower IPV prevalence, while countries in darker red have higher prevalence.

Data on IPV was available for 368,302 ever-partnered women aged 15–49 years, from 48 LMICs. The characteristics of the overall sample are presented in Table S3. The reported 12-month physical and/or sexual IPV prevalence varied significantly across countries with a median prevalence of 18.2% (interquartile range 11.2–25.5%). Papua New Guinea had the highest IPV prevalence: 47.6% (95% confidence interval 44.0–51.1%); followed by Afghanistan with 46.0% (43.9–48.2%); Congo DR with 36.7% (33.9–39.7%); Timor-Leste with 34.6% (32.4–36.9%) and Colombia with 33.3% (32.2–34.3%). Armenia had the lowest IPV prevalence across the countries studied, 3.5% (2.8–4.4%). National IPV levels are presented in Fig. 1 and Table S4. The median proportion of women for whom the necessary privacy was not obtained for administering the domestic violence module was 0.5%, ranging from 0.0% in Nepal and Tanzania, to 8.6% in South Africa (Table S4).

### 3.2 Pooled decision tree

**Fig. 2:**
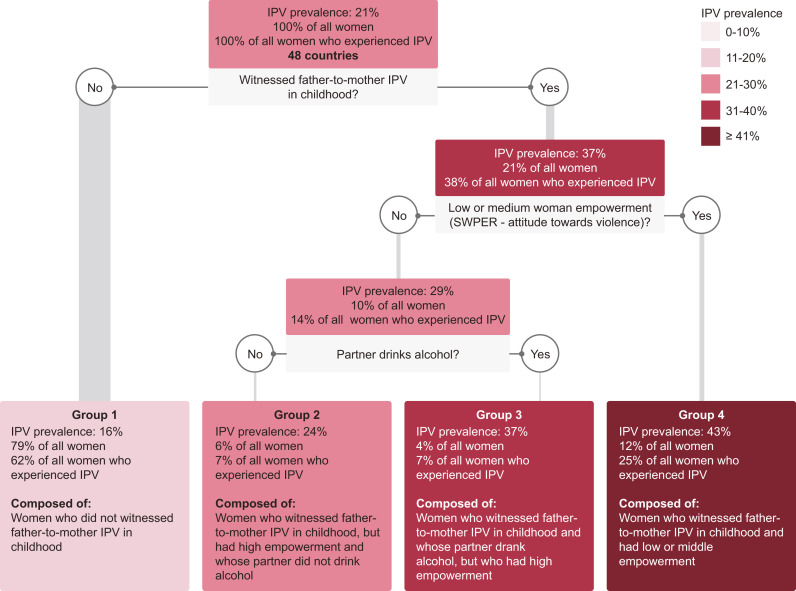
Decision tree for all countries. For each node (coloured box) in the decision tree, the following are presented: (1) the IPV prevalence amongst the women assigned to that node; (2) the percentage of all women in the sample who were assigned to that node; and (3) the percentage of all women who experienced IPV in the sample who were assigned to that node. Nodes in lighter pink have lower IPV prevalence, while nodes in darker red have higher prevalence. Grey boxes show the indicators used for splitting the group which they are directly below, as well as their respective cut off points. The final nodes (Groups 1–4) have a direct textual description of how they are composed, based on the splitting criteria that generated those groups.

The decision tree pooling data for the 48 LMICs divided the sample into 4 groups of women with rather different levels of IPV (Fig. 2). Three variables were selected by the model for grouping the women: having witnessed IPV during childhood, low or medium empowerment level in the attitude to violence SWPER domain and partner's current alcohol use. The group with the highest prevalence of IPV (Group 4) included women who witnessed IPV during childhood and with a low or medium empowerment level. 43.3% (95% CI 41.9–44.7) of women in this group experienced IPV, and they represent 12% of all women and 25% of all women experiencing IPV. The second highest prevalence of IPV (37.2%; 95% CI 34.9–39.6) was amongst women who witnessed IPV in childhood, had a high empowerment level and a partner who drinks alcohol (Group 3). Next, group 2 was composed of women similar to group 3, but whose partners did not drink alcohol (IPV prevalence of 23.6%; 95% CI 22.3–25.1). The lowest prevalence of IPV was found amongst women who did not witness IPV during childhood (group 1, prevalence of 16.1%; 95% CI 15.8–16.5).

### 3.3 National trees and country profiles

Individual country profiles are presented in the appendix (Figs. S1–S96). Of the 48 LMICs included in the analysis, 32 had trees that successfully split the women into high-risk groups. For the remaining 16 countries (Armenia, Burkina-Faso, Comoros, Dominican Republic, Gambia, Guatemala, Haiti, Honduras, Maldives, Myanmar, Nepal, Peru, Philippines, Rwanda, South Africa, and Togo), no specific indicator was found to predict woman´s exposure to IPV. These countries had a median IPV prevalence of 10.7% (interquartile range 6.9–11.6%), while the other 32 had a median of 23.4% (IQR 17.3–28.8%). Lower national prevalence makes the identification of high-risk groups less likely.

The indicators frequency across the 48 countries are presented on Table 1. Partner's alcohol use and witnessing father-mother IPV during childhood emerged as frequent markers of IPV risk in nearly half of the countries. With a lower frequency we had women's age and empowerment level, in 13 and 12 countries, respectively. One important difference for the national trees, when compared with the global one, is the addition of *subnational regions* as a potential indicator of regional inequalities in IPV risk, which was present in 25 of all national trees, making it the most frequent of all indicators that emerged (Table 1). One national example of significant regional variation in IPV levels is illustrated in Fig. 2 (Kenya).

**Table 1:** Indicators of IPV risk featured in the 48 national decision trees.

Country	Year	Subnational regions	Partner's alcohol use	Witnessed father-to-mother IPV in childhood	Woman's age	Woman's empowerment – attitude to violence	Partner's education	Partner's occupation	Woman's empowerment – decision making	Wealth quintiles	Number of living children	Woman's education	Woman's empowerment – social independence	Polygamy	Area of residence (urban/rural)	Religious affiliation
Afghanistan	2015	✓		✓	✓	✓										
Angola	2015	✓	✓		✓	✓						✓				
Armenia	2015															
Benin	2017	✓		✓												
Burkina Faso	2010															
Burundi	2016		✓		✓	✓										
Cambodia	2014	✓		✓			✓									
Cameroon	2018	✓	✓													
Chad	2014	✓	✓		✓					✓						
Colombia	2015			✓							✓					
Comoros	2012															
Congo, DR	2013	✓	✓	✓	✓		✓		✓					✓		
Côte d'Ivoire	2011	✓	✓						✓							
Dominican Republic	2013															
Egypt	2014			✓	✓	✓										
Ethiopia	2016	✓	✓	✓					✓	✓			✓			
Gabon	2012		✓			✓	✓		✓				✓			
Gambia	2013															
Guatemala	2014															
Haiti	2016															
Honduras	2011															
India	2015	✓	✓	✓		✓										
Jordan	2017	✓		✓												
Kenya	2014	✓	✓	✓												
Kyrgyzstan	2012	✓	✓	✓		✓		✓								
Malawi	2015	✓	✓		✓		✓	✓	✓							
Maldives	2016															
Mali	2018		✓	✓				✓								
Mozambique	2011	✓	✓	✓				✓								
Myanmar	2015															
Namibia	2013	✓	✓	✓			✓									
Nepal	2016															
Nigeria	2018	✓	✓	✓												
Pakistan	2017	✓		✓	✓						✓	✓				
Papua New Guinea	2016		✓	✓	✓											
Peru	2018															
Philippines	2017															
Rwanda	2014															
Senegal	2017	✓	✓	✓												
Sierra Leone	2013	✓		✓	✓	✓		✓								
South Africa	2016															
Tajikistan	2017	✓	✓	✓	✓		✓			✓	✓					
Tanzania	2015	✓	✓		✓	✓										
Timor-Leste	2016	✓	✓			✓										
Togo	2013															
Uganda	2016	✓	✓			✓				✓						
Zambia	2018	✓	✓	✓		✓	✓									
Zimbabwe	2015		✓	✓	✓					✓						
Number of national trees that featured the variable		25	24	22	13	12	7	5	5	5	3	2	2	1	0	0

**Fig. 3:**
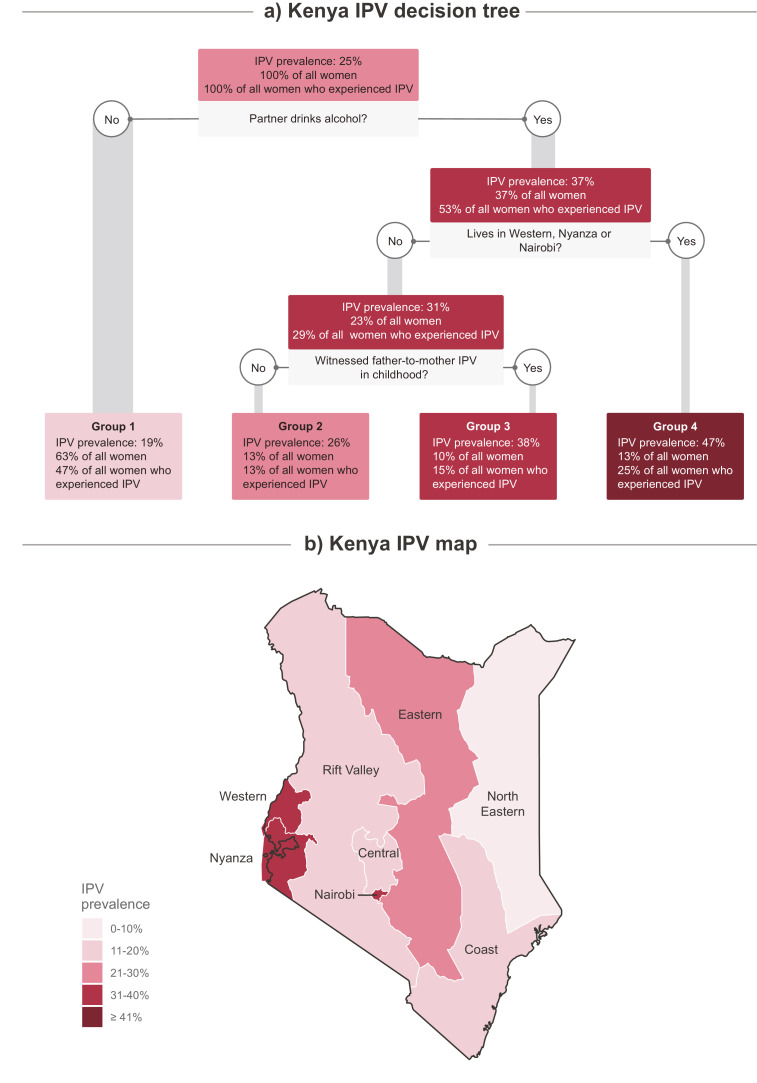
Kenya subnational variations in IPV levels. The figure is divided in two panels: the top panel contains the Kenya's decision tree and the bottom panel an IPV prevalence map, showing subnational regions. Nodes in the decision tree and regions in the map coloured in lighter pink have lower IPV prevalence, while those in darker red have higher prevalence. For each node in the decision tree, the following are presented: (1) the IPV prevalence amongst the women assigned to that node; (2) the percentage of all women in the sample who were assigned to that node; and (3) the percentage of all women who experienced IPV in the sample who were assigned to that node. Grey boxes show the indicators used for splitting the group which they are directly below, as well as their respective cut off points.

Fig. 3 represents a case study Kenya. In Kenya, partner´s alcohol comsumption was a marker of increased risk for IPV, nearly doubling the prevalence when compared to women whose partner does not drink. For women living in the Western, Nyanza or Nairobi regions (a) this prevalence rises further to 47%. As observed in the map (b), significant regional variability in the proportion of IPV exists across Kenya suggesting the presence of relevant context-specific factors in determining IPV vulnerability. The grade of patriarchy, as well as ethnic and religious beliefs are known to play a key role in the social acceptability of violence in Kenya and might be behind the regional differences found [24]. Western and Nyanza are two neighbouring regions with ethnic communities that share cultural practices that predispose women to violence in their relationships. For instance, widow cleansing, wife inheritance and sexual practices like the requirement for a couple to engage in sexual intercourse before major life events [25,26]. Widow cleansing, a ritual where a woman is culturally required to have intercourse with her husband's relative or a paid ‘cleanser’ after her husband's death as well as wife inheritance where a widow risk being ostracized if they are not inherited by her deceased husband's relative contribute to curtailing women's rights and freedoms within these intimate relationships. Luo and Luhya women, the predominant ethnic groups in Nyanza and Western, respectively – and who have the aforementioned practices – also report higher levels of IPV compared to Kikuyu women, a community which does not have these practices and is predominantly found in the Central region [25]. Moreover, the majority of women in these regions live in rural communities and are more likely to: be in polygynous relationships; have achieved less than secondary education; marry at a younger age; and, have their husband solely deciding how household earnings are spent compared to the national average – all factors that increase women's vulnerability to IPV [27]. Women in Western are also more likely to justify wife beating (52%) compared to the national average (42%) [28].

Nairobi – the other region with higher prevalence of IPV – is the capital city of Kenya. It comprises a multi-ethnic population with over 60% of its inhabitants residing in slums which only cover about 5% of the residential area [29,30]. Compared to other areas in Nairobi and the general population, IPV levels in the informal settlements are significantly higher: studies in two of the largest slums, Kibera and Mathare, found that 85% and 66% of women have experienced IPV respectively [31,32]. Populations in these informal settlements are highly mobile, have poor education attainment, low economic status, high crime rates and lack of well-functioning governance systems [33]. These are all factors that can contribute to increased levels of violence in the community.

Overall, the three subnational regions – Western, Nyanza and Nairobi – also manifest higher levels of exposure to alcohol when compared to the national average and to areas of low IPV prevalence. For instance, Nairobi has the highest prevalence of alcohol consumption in the whole country amongst both men (40%) and women (10%) compared to a national average of 30% and 5%, respectively [28]. Alcohol consumption prevalence in rural communities in Western can be up to two times the national prevalence [34,35]. In contrast, the North Eastern region, which has the lowest level of IPV, also has the lowest prevalence of alcohol consumption in the country. This suggests that alcohol may amplify the effect of, and/or, act synergistically with other factors and sociocultural norms supportive of IPV in these regions.

## 4 Discussion

This study contributes evidence that can be used to plan and improve the design of policies and programmes aimed at reducing population levels of IPV by in LMICs. By using a decision-tree approach - a simple-to-use yet powerful statistical tool we provide important insights about subpopulations of women where IPV is concentrated and whose characteristics could be used for prevention and tracing of women experiencing IPV. When considering all countries together, witnessing IPV during childhood, having a lower empowerment in the attitude to violence domain, and having a partner who uses alcohol were the most relevant markers of IPV vulnerability. Amongst the highest-risk group of women, including those who witnessed IPV during childhood and had lower empowerment levels, IPV prevalence were more than double of that observed in the overall sample (43% vs. 21%). Still, the group with the lowest risk had an IPV prevalence of 16% and represented a large portion of those who experienced IPV (62%). This finding confirms the pervasiveness of IPV and how women from vastly different backgrounds can still experience it. Unfortunately, no safe group of women was identified.

Though there were significant variations in the number of subgroups identified by the national trees, there was great similarity between the most frequent markers of IPV risk featured along the individual trees and the pooled tree. In the country-level analysis, however, the subnational regions were included and emerged as the commonest markers of IPV risk, indicating the presence of substantial geographical disparities in IPV levels in many of the countries (53% of the national trees). As exemplified by the case study developed for Kenya, a potential explanation for the subnational inequalities could be that diverse factors can act synergistically to elevate the regional impact on IPV levels. Perhaps individual factors that are not strong enough to be selected by the decision tree may have a cumulative effect when combined with other factors within these regions, resulting in higher regional IPV prevalence. Although not accessed in our study, the existence of other gender, economic and political-legal structural disparities such as lack of economic rights and ease of divorce for women, might also be behind the subnational variations encountered [13]. Another possible fact that could help to explain at least partially why the subnational regions were the preeminent indicator in the national trees is the fact that CART tends to select indicators with many possible splitting points (such as indicators with many categories) [15]. We tried to limit this effect by ruling out any subgroup with less than 50 women, thus avoiding conclusions based on small subgroups. Nevertheless, any inferences based on subnational regions should be interpreted with caution, considering the national context as we did for Kenya case study.

Because the individual risk and vulnerability for IPV is known to arise from gender inequity manifestations operating at different levels of the social ecology, our overall findings support greater emphasis on policy reforms at the macro-level and the design of interventions that take these cross-level effects into account as the responsibility for change should not only be targeted to women [13,36]. In LMICs, despite the scarcity of evidence, promising violence prevention programmes have been commonly participatory, engage multiple stakeholders, and are based on theories of gender and social empowerment that view behaviour change as a collective effort, supporting greater communication and shared decision-making amongst family members, as well as non-violent behaviour. One encouraging case of community intervention engaging stakeholders at many levels is the SASA! programme in Uganda, designed to prevent violence against women at the populational level by shifting the power imbalance between men and women in relationships through complementary approaches targeted to both individuals and communities [37].

By identifying witnessing IPV during childhood and lower women´s empowerment amongst the most important factors featured in the trees, our findings reinforce the relevance of strategies challenging harmful gender attitudes, beliefs, norms, and stereotypes and aiming to break the intergenerational cycle of violence transmission such as those aimed to stablish gender equitable and nurturing care relationships by parents. These are indeed relevant components to also be considered in the design of commonly seen social-structural interventions (e.g., women´s income generation). The identification of partner alcohol use as an widespread marker of at-risk women across countries also brings up the potential for coordinated and intersectoral responses, particularly in the context of concentrated disadvantage and high levels of interpersonal violence within families [38,39]. All these factors emphasize the relevance of studies that measure the characteristics of not just women but their partners for informing preventive interventions that will accounts for women's experiences of IPV but also the critical role of perpetrating partners’ circumstances and the importance of engaging men and boys for increased intervention success.

Despite the high consistency on characteristics identifying the high-risk woman in the pooled tree and the individual national trees, however, many particularities on the underlying risk factors for IPV are observed at the country level which holds promise for context specific programming and are a key potential of this study. Lower partner´s education, for example, seems to be an important marker of IPV risk in a few LMICs although not present in the pooled tree. The decision tree approach used in the present work is exactly suitable for deciding who might benefit from receiving an intervention considering the particularities of each setting. Instead of trying to work with all women in a community, a short questionnaire applied to women when they seek care in a primary health care facility can flag women at higher risk. Taken the results of the pooled tree as an example, it is one or two questions about witnessing IPV in the past, plus five questions on attitude toward violence. With this simple tool, a group that is reduced to around 10% of female population, with an estimated prevalence of violence around 40% could be invited for discussion groups, support groups or even to a more in-depth interview depending on the local context and resources.

Some limitations should be considered when interpreting the findings. Although the characteristics included provide an important appraisal of the most vulnerable groups of women, we were unable to include some relevant predictors of IPV risk in our analysis because they were not assessed in DHS (e.g. woman´s mental health, early childhood experiences of violence, presence of disabilities, and transgender identity) [3]. Because our work is limited to whatever is available on surveys, the methods used here could be adjusted and tailored to country-specific situations where possibly more relevant traits could be used. It is also important to note that the countries included in our analysis represent 35% of all LMICs, with varying representativeness across UNICEF regions (Table S5). For this reason, the pooled tree should be interpreted as a summary of evidence for the countries with available data but not necessarily representative of all LMICs.

The choice of parameters used by CART can affect the trees that are created. We have evaluated our results’ sensitivity to those parameters testing 24 different scenarios (Supplementary Materials). In general, the indicators selected during the pooled tree creation are consistent. The partner's alcohol use was the most unstable indicator, being included or not in the final pooled tree depending on the choice of some of the parameters, as well as splitting other subgroups, such as women who did not witness father-to-mother IPV in childhood. This is likely related to fact that alcohol use varies greatly between regions – with a country like Afghanistan, for example, having the second highest IPV prevalence and virtually no reported alcohol use.

Under-reporting is an important issue in IPV research and is likely to vary with woman´s characteristics as well as contextual factors such as cultural and social norms. As so, we underscore the possibility of a significant underestimate of IPV prevalence which can have varying levels depending on the country. However, although the prevalence of IPV varied substantially across the countries studied – from 47.6% in Papua New Guinea to 3.5% in Armenia, the general pattern of characteristics identifying the most vulnerable groups of women proved to be consistent. The use of a 12-month period to define IPV and ignoring the level of abuse may lead to mixing very different profiles of women victim of IPV. There might be women who recently experienced one or a few incidents and might possibly leave their relationships (more common in places where it is easier for women to get divorced) as well as those who have been experiencing IPV for a long time (prior to and during the past 12 months). Still concerning the IPV measure, frequency and severity were not investigated in our study, and we encourage future work to explore these aspects for the identification of high-risk women. Finally, while recognizing the significant adverse impacts that psychological violence can have on women's physical and mental health, we opted not to consider it in the present work as there is strong recommendation in the field for not combining it with the other types of IPV [40].

In conclusion, this study provides a comprehensive understanding of the patterns of risk factors identifying women most at risk of IPV across 48 countries with important implications for optimizing the design of IPV-focused interventions in LMICs. Because the trees were based on known risk factors for IPV in the broad literature, our findings usurpingly confirm most of what is already known in terms of factors that place women at higher risk of IPV. The illustration of how these interact in different countries and regions, the selection of those most relevant to identifying high risk groups where they exist as well as the estimation of IPV prevalence in each of these groups is, however, a great advance of the present study. The individual country profiles generated have the potential to guide policymakers and program planners on where to prioritize investment by targeting risk groups and/or tailoring intervention content to specific contextually relevant drivers of IPV within their countries. On the other hand, the factors that emerged in the pooled analysis can be thought as common contributing causes that places women at increased risk of IPV and could be addressed in interventions that want to be transferrable across contexts. A large proportion of women who experienced IPV, however, had no indicators that could help with either targeting or tailoring interventions indicating the need for higher population awareness about the issue as well as continued improvements in legislation, access to health and protective services and data collection. Future studies on the determinants of the geographical distribution of IPV can provide useful insights for violence prevention, especially if included in household surveys such as DHS, alloying their routine monitoring and further risk groups identification.

## Contributors

Conceptualization, C.V.N.C., T.M.S. and A.J.D.B.; data curation, C.V.N.C., T.M.S. and A.J.D.B.; formal analysis, C.V.N.C and T.M.S.; funding acquisition, A.J.D.B.; investigation, C.V.N.C. and T.M.S.; methodology, C.V.N.C.,T.M.S. and A.J.D.B.; project administration, C.V.N.C. and A.J.D.B.; resources, F.K., F.B., A.J.D.B.; software, T.M.S.; supervision, A.J.D.B.; validation, C.V.N.C. and T.M.S.; visualization, C.V.N.C., T.M.S., and A.J.D.B.; writing—original draft, C.V.N.C.,T.M.S., and A.G.; writing—review and editing, C.V.N.C., T.M.S., K.D. F.K., F.B., A.G., G.M.H., and A.J.D.B. All authors have read and agreed to the published version of the manuscript.

## Data sharing statement

All data relevant to the study are included in the article or available as supplementary information. The data used in the analyses is publicly available, anonymised and geographically scrambled to ensure confidentiality. More information on DHS can be found at https://dhsprogram.com/, where survey datasets can be obtained.

## Funding

Bill and Melinda Gates Foundation (Grant INV-010051/OPP1199234), 10.13039/100010269Wellcome Trust (Grant Number: 101815/Z/13/Z) and Associação Brasileira de Saúde Coletiva (ABRASCO).

## Declaration of Competing Interest

FK is the co-chair of the Lancet Commission on Gender-based Violence and Maltreatment of Young People and has received a research grant from Merck KGaA/EMD Serono in support of the commission. FK is also a member of the Board of Directors, Esperanza United. No conflicts of interest were declared by the other authors.

## Appendix Supplementary materials

Image, application 1

Image, application 2
