# Supplementary material for: Identifying the women most vulnerable to intimate partner violence: A decision tree analysis from 48 low and middle-income countries
Source: eClinicalMedicine. 2021 Dec 2;42:101214. doi: 10.1016/j.eclinm.2021.101214 (PMC8712229; doi:10.1016/j.eclinm.2021.101214)
Supplement: Supplementary file 1 [file mmc1.docx]

# Caption for supplementary material

Caption for: Supplementary materials.docx

Supplementary Table S1 – Country, year, UNICEF region, World Bank income group and number of women for the surveys included in the sample

Supplementary Table S2 – Availability of indicators among surveys

Supplementary Table S3 – Women’s sample description. Includes a description of the age group, the area of residence, the wealth index, the highest educational level and the number of children

Supplementary Table S4 – Percentage of women not interviewed because privacy could not be obtained, prevalence of intimate partner violence against women and of key covariates (women with high empowerment in attitude towards violence domain, partner’s alcohol use and women who witnessed violence in childhood)

Supplementary Table S5 – Representativeness of the sample according to UNICEF regions

Supplementary Figures S1-S96 – Country profile and decision tree of 48 countries in the study. The country profile contains the prevalence of physical or sexual IPV combined and separated as well as the prevalence of women in each group of empowerment, level of education and area of residence. It also includes the prevalence of women who witnessed father-to-mother IPV in childhood and who have a partner who drinks alcohol or has extra wives. For each node (coloured box) in the decision tree, the following are presented: 1) the IPV prevalence among the women assigned to that node; 2) the percentage of all women in the sample who were assigned to that node; and 3) the percentage of all women who experienced IPV in the sample who were assigned to that node. Nodes in lighter pink have lower IPV prevalence, while nodes in darker red have higher prevalence. Below each group is presented the indicators used for splitting that group, as well as their respective cut off points.

Supplementary Table S6 – Results for the sensitivity analysis performed, including the parameters used in each test, the resulting number of nodes and which variables are included in the pooled tree that was created.

Supplementary Figures S97-S108 – Decision trees created for the sensitivity analysis. For each node (coloured box) in the decision tree, the following are presented: 1) the IPV prevalence among the women assigned to that node; 2) the percentage of all women in the sample who were assigned to that node. Nodes in lighter pink have lower IPV prevalence, while nodes in darker red have higher prevalence. Below each group is presented the indicators used for splitting that group, as well as their respective cut off points.
