# Supplementary material for: Identifying the women most vulnerable to intimate partner violence: A decision tree analysis from 48 low and middle-income countries
Source: eClinicalMedicine. 2021 Dec 2;42:101214. doi: 10.1016/j.eclinm.2021.101214 (PMC8712229; doi:10.1016/j.eclinm.2021.101214)
Supplement: Supplementary file 2 [file mmc2.docx]

**Table S1 – DHS surveys selected for the analyses**

| **Country** | **Year** | **UNICEF Region** | **World Bank Income Group** | **Number of women** |
| --- | --- | --- | --- | --- |
| Afghanistan | 2015 | South Asia | Lower | 21324 |
| Angola | 2015 | Eastern and Southern Africa | Upper middle | 7669 |
| Armenia | 2015 | Eastern Europe and Central Asia | Lower middle | 3540 |
| Benin | 2017 | West and Central Africa | Lower | 4488 |
| Burkina Faso | 2010 | West and Central Africa | Lower | 10009 |
| Burundi | 2016 | Eastern and Southern Africa | Lower | 7366 |
| Cambodia | 2014 | East Asia and Pacific | Lower | 3499 |
| Cameroon | 2018 | West and Central Africa | Lower middle | 4690 |
| Chad | 2014 | West and Central Africa | Lower | 3814 |
| Colombia | 2015 | Latin America and Caribbean | Upper middle | 24862 |
| Comoros | 2012 | Eastern and Southern Africa | Lower | 2529 |
| Congo, D.R. | 2013 | West and Central Africa | Lower | 5691 |
| Côte d’Ivoire | 2011 | West and Central Africa | Lower middle | 5018 |
| Dominican Republic | 2013 | Latin America and Caribbean | Upper middle | 5803 |
| Egypt | 2014 | Middle East and North Africa | Lower middle | 6693 |
| Ethiopia | 2016 | Eastern and Southern Africa | Lower | 4720 |
| Gabon | 2012 | West and Central Africa | Upper middle | 4147 |
| Gambia | 2013 | West and Central Africa | Lower | 3542 |
| Guatemala | 2014 | Latin America and Caribbean | Lower middle | 6512 |
| Haiti | 2016 | Latin America and Caribbean | Lower | 4322 |
| Honduras | 2011 | Latin America and Caribbean | Lower middle | 12494 |
| India | 2015 | South Asia | Lower middle | 66013 |
| Jordan | 2017 | Middle East and North Africa | Upper middle | 6852 |
| Kenya | 2014 | Eastern and Southern Africa | Lower | 4519 |
| Kyrgyzstan | 2012 | Eastern Europe and Central Asia | Lower middle | 4832 |
| Malawi | 2015 | Eastern and Southern Africa | Lower | 5406 |
| Maldives | 2016 | South Asia | Upper middle | 3388 |
| Mali | 2018 | West and Central Africa | Lower | 3356 |
| Mozambique | 2011 | Eastern and Southern Africa | Lower | 5824 |
| Myanmar | 2015 | East Asia and Pacific | Lower | 3425 |
| Namibia | 2013 | Eastern and Southern Africa | Upper middle | 1449 |
| Nepal | 2016 | South Asia | Lower | 3826 |
| Nigeria | 2018 | West and Central Africa | Lower middle | 8910 |
| Pakistan | 2017 | South Asia | Lower middle | 4085 |
| Papua New Guinea | 2016 | East Asia and Pacific | Lower middle | 3955 |
| Peru | 2018 | Latin America and Caribbean | Upper middle | 22604 |
| Philippines | 2017 | East Asia and Pacific | Lower middle | 13215 |
| Rwanda | 2014 | Eastern and Southern Africa | Lower | 1908 |
| Senegal | 2017 | West and Central Africa | Lower middle | 2660 |
| Sierra Leone | 2013 | West and Central Africa | Lower | 4315 |
| South Africa | 2016 | Eastern and Southern Africa | Upper middle | 2354 |
| Tajikistan | 2017 | Eastern Europe and Central Asia | Lower | 5313 |
| Tanzania | 2015 | Eastern and Southern Africa | Lower | 7597 |
| Timor-Leste | 2016 | East Asia and Pacific | Lower middle | 3694 |
| Togo | 2013 | West and Central Africa | Lower | 5376 |
| Uganda | 2016 | Eastern and Southern Africa | Lower | 7536 |
| Zambia | 2018 | Eastern and Southern Africa | Lower middle | 7358 |
| Zimbabwe | 2015 | Eastern and Southern Africa | Lower | 5800 |

**Table S2 – Availability of indicators among surveys**

| **Indicator** | **Countries with indicator available^1^** |
| --- | --- |
| Area of residence (urban/rural) | All |
| Number of living children | All |
| Partner’s alcohol use | All but Colombia and Jordan |
| Partner’s education | All |
| Partner’s occupation | All but Colombia |
| Polygamy | All but Armenia, Colombia, Dominican Republic, Guatemala, Honduras, Kyrgyzstan and Peru |
| Religious affiliation | All but Afghanistan, Armenia, Colombia, Jordan, Kyrgyzstan, Maldives, Myanmar, Pakistan, Peru, South Africa, Tajikistan and Tanzania |
| Subnational regions | All |
| Wealth quintiles | All |
| Witnessed father-to-mother IPV in childhood | All |
| Woman’s age | All |
| Woman’s education | All |
| Woman’s empowerment – attitude to violence | All but Colombia, Jordan, Maldives and Tajikistan |
| Woman’s empowerment – decision making | All but Colombia, Jordan, Maldives and Tajikistan |
| Woman’s empowerment – social independence | All but Colombia, Jordan, Maldives and Tajikistan |

^1^At least one woman in the dataset with available information

**Table S3 – Women’s sample description**

| **Characteristic** | **Group** | **Percentage** |
| --- | --- | --- |
| Age group | 15-19 years | 4.2% |
|  | 20-29 years | 34.8% |
|  | 30-39 years | 34.9% |
|  | 40-49 years | 26.0% |
| Area of residence | Urban | 37.1% |
|  | Rural | 62.9% |
| Wealth index | Poorest | 17.9% |
|  | Second | 19.7% |
|  | Third | 20.5% |
|  | Fourth | 20.8% |
|  | Wealthiest | 21.1% |
| Highest educational level | No education | 32.9% |
|  | Primary | 20.1% |
|  | Secondary | 35.8% |
|  | Higher | 11.1% |
| Number of children | 0 children | 9.8% |
|  | 1 child | 18.6% |
|  | 2 children | 27.4% |
|  | 3 children | 18.5% |
|  | 4+ children | 25.7% |

**Table S4 – Percentage of women not interviewed because privacy could not be obtained, prevalence of intimate partner violence against women and of key covariates**

|  |  |  | **Prevalence (%) of** | | | |
| --- | --- | --- | --- | --- | --- | --- |
| **Country** | **Year** | **% privacy not obtained** | **women who experienced IPV** | **women with high empowerment (attitude to violence)** | **partner’s alcohol use** | **women who witnessed violence in childhood** |
| Afghanistan | 2015 | 2.3 | 46.0 | 14.1 | 0.7 | 48.9 |
| Angola | 2015 | 4.3 | 25.8 | 71.6 | 42.1 | 31.0 |
| Armenia | 2015 | 0.9 | 3.5 | 86.0 | 63.5 | 6.5 |
| Benin | 2017 | 0.5 | 13.9 | 66.5 | 27.8 | 10.2 |
| Burkina Faso | 2010 | 0.1 | 9.2 | 53.5 | 26.3 | 8.8 |
| Burundi | 2016 | 0.1 | 27.8 | 38.6 | 68.2 | 41.3 |
| Cambodia | 2014 | 0.2 | 10.9 | 44.0 | 84.9 | 19.7 |
| Cameroon | 2018 | 0.6 | 21.5 | 68.1 | 45.9 | 22.8 |
| Chad | 2014 | 1.2 | 17.4 | 25.0 | 31.2 | 22.0 |
| Colombia | 2015 | 1.6 | 33.3 | - | - | 36.6 |
| Comoros | 2012 | 0.9 | 4.8 | 57.4 | 2.6 | 4.8 |
| Congo, D.R. | 2013 | 0.2 | 36.7 | 23.2 | 51.1 | 36.9 |
| Côte d’Ivoire | 2011 | 0.4 | 23.0 | 49.8 | 35.1 | 14.7 |
| Dominican Republic | 2013 | 0.1 | 15.6 | 97.6 | 67.4 | 15.9 |
| Egypt | 2014 | 0.8 | 14.0 | 63.6 | 0.7 | 19.2 |
| Ethiopia | 2016 | 1.1 | 19.7 | 32.5 | 31.6 | 29.1 |
| Gabon | 2012 | 0.4 | 31.2 | 48.2 | 62.4 | 39.9 |
| Gambia | 2013 | 0.3 | 7.3 | 37.0 | 1.4 | 10.5 |
| Guatemala | 2014 | 0.1 | 8.5 | 88.7 | 39.7 | 33.9 |
| Haiti | 2016 | 0.1 | 13.8 | 83.7 | 37.6 | 12.9 |
| Honduras | 2011 | 0.1 | 10.9 | 86.2 | 38.1 | 27.7 |
| India | 2015 | 0.5 | 23.7 | 53.5 | 28.9 | 21.8 |
| Jordan | 2017 | 0.6 | 13.8 | - | - | 7.3 |
| Kenya | 2014 | 0.1 | 25.4 | 56.2 | 36.7 | 40.0 |
| Kyrgyzstan | 2012 | 0.2 | 17.1 | 56.2 | 39.2 | 15.4 |
| Malawi | 2015 | 1.0 | 24.1 | 84.5 | 30.9 | 27.1 |
| Maldives | 2016 | 5.2 | 5.5 | - | 2.1 | 10.0 |
| Mali | 2018 | 1.4 | 20.9 | 19.1 | 4.3 | 11.1 |
| Mozambique | 2011 | 0.6 | 27.7 | 75.8 | 38.9 | 30.4 |
| Myanmar | 2015 | 0.2 | 11.0 | 44.7 | 46.8 | 18.9 |
| Namibia | 2013 | 0.3 | 20.2 | 68.5 | 57.7 | 27.5 |
| Nepal | 2016 | 0.0 | 11.2 | 71.0 | 44.3 | 14.6 |
| Nigeria | 2018 | 0.4 | 13.7 | 71.3 | 23.1 | 10.4 |
| Pakistan | 2017 | 0.9 | 14.5 | 57.7 | 3.6 | 21.9 |
| Papua New Guinea | 2016 | 0.9 | 47.6 | 24.4 | 59.3 | 49.0 |
| Peru | 2018 | 0.9 | 10.9 | 98.4 | 78.5 | 42.2 |
| Philippines | 2017 | 0.8 | 5.4 | 88.3 | 64.5 | 19.5 |
| Rwanda | 2014 | 0.1 | 20.6 | 62.5 | 64.8 | 40.8 |
| Senegal | 2017 | 0.2 | 12.2 | 46.9 | 2.4 | 3.8 |
| Sierra Leone | 2013 | 0.4 | 28.6 | 31.1 | 16.7 | 36.1 |
| South Africa | 2016 | 8.6 | 10.5 | 94.9 | 40.3 | 16.0 |
| Tajikistan | 2017 | 3.6 | 19.0 | - | 18.0 | 13.9 |
| Tanzania | 2015 | 0.0 | 29.5 | 38.9 | 33.5 | 39.4 |
| Timor-Leste | 2016 | 3.2 | 34.6 | 19.5 | 35.9 | 19.5 |
| Togo | 2013 | 0.2 | 12.7 | 69.0 | 46.6 | 16.9 |
| Uganda | 2016 | 0.5 | 29.6 | 49.7 | 43.2 | 37.9 |
| Zambia | 2018 | 1.4 | 25.1 | 52.6 | 37.7 | 30.7 |
| Zimbabwe | 2015 | 0.2 | 19.8 | 63.2 | 40.0 | 38.1 |

**Table S5 – Representativeness of the sample according to UNICEF regions**

| **UNICEF Region** | **Number of LMIC^1^** | **Number of LMIC in sample** | **% of LMIC in sample** | **% of women aged 15-49 years from LMIC countries in sample^2^** |
| --- | --- | --- | --- | --- |
| West and Central Africa | 24 | 13 | 54% | 83% |
| Eastern and Southern Africa | 24 | 14 | 58% | 81% |
| Middle East and North Africa | 12 | 2 | 17% | 27% |
| Eastern Europe and Central Asia | 20 | 3 | 15% | 4% |
| East Asia and Pacific | 23 | 5 | 22% | 9% |
| South Asia | 8 | 5 | 63% | 89% |
| Latin America and Caribbean | 26 | 6 | 23% | 21% |

^1^World Bank income classification from the median year of all surveys (2015);

^2^WHO population estimates of women aged 15-49 years in 2015. Data not available for Dominica, Marshall Islands, Palau, Tuvalu and West Bank and Gaza, which were estimated based on the average percentage of women aged 15-49 years in the total population of the other countries. World Bank estimates of total population.

**Country profiles**


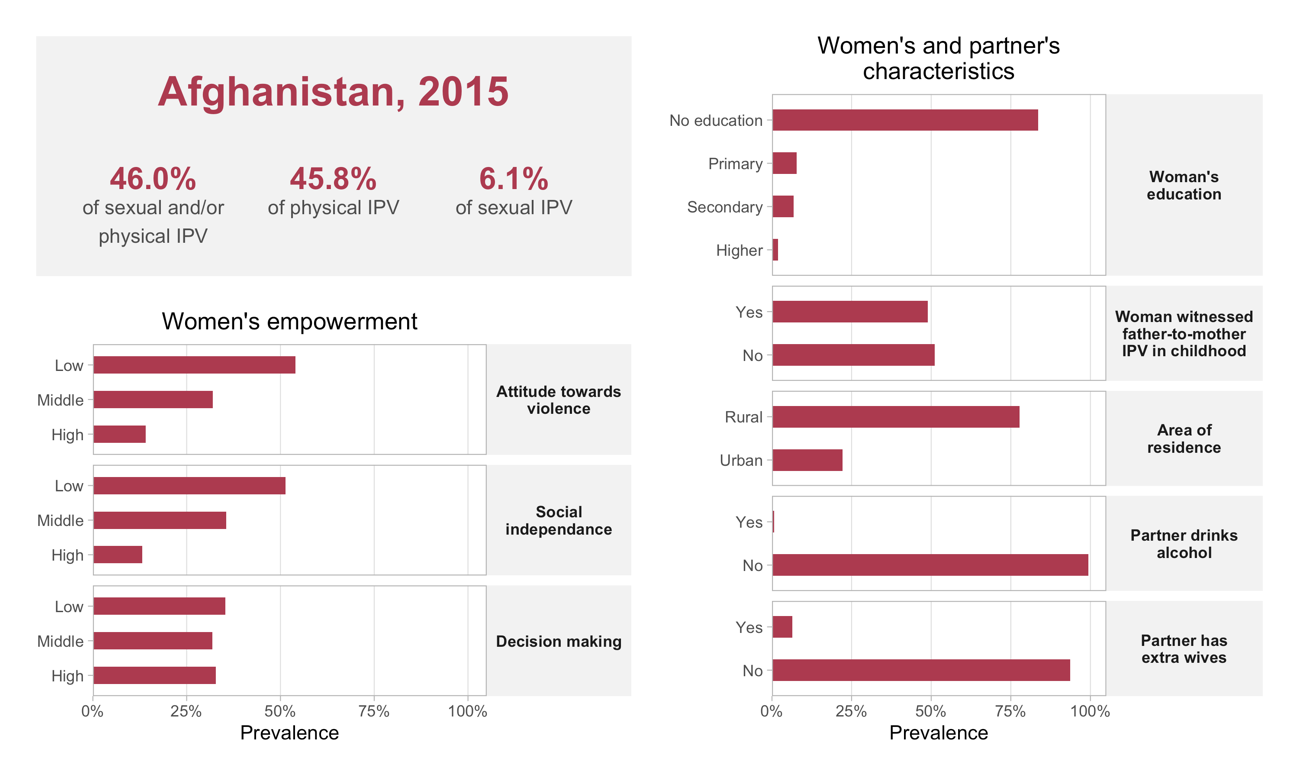


Figure S1 – Afghanistan, 2015: country profile


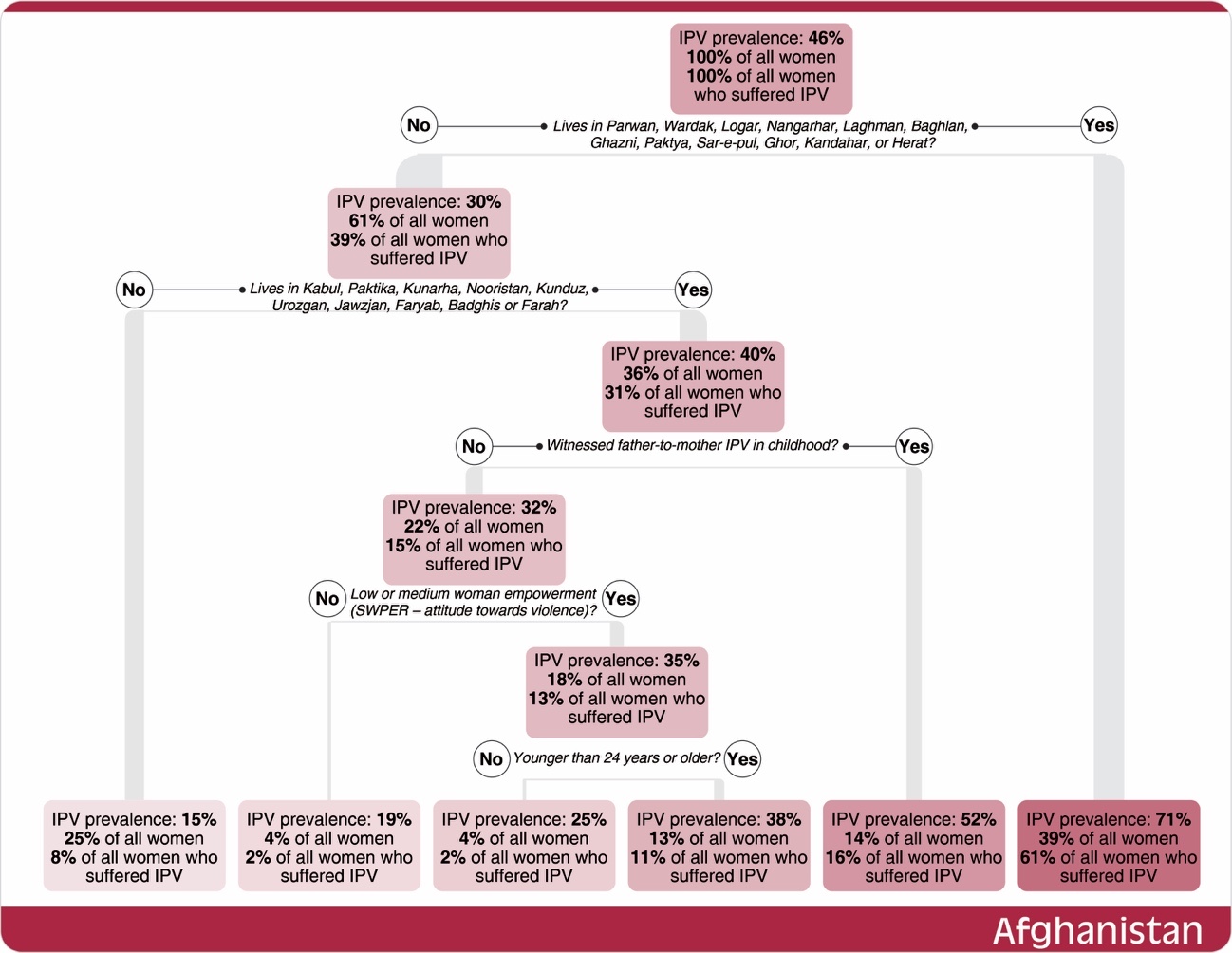


Figure S2 – Afghanistan, 2015: decision tree


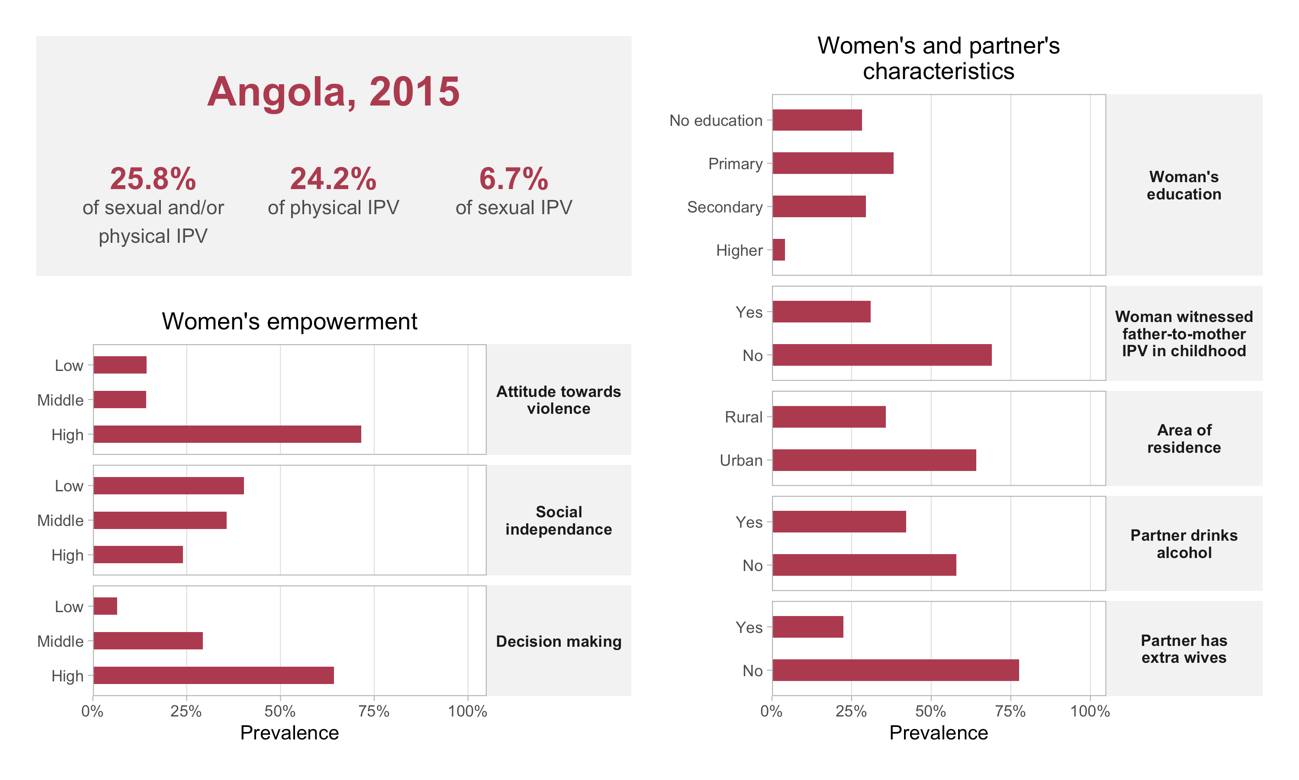


Figure S3 – Angola, 2015: country profile


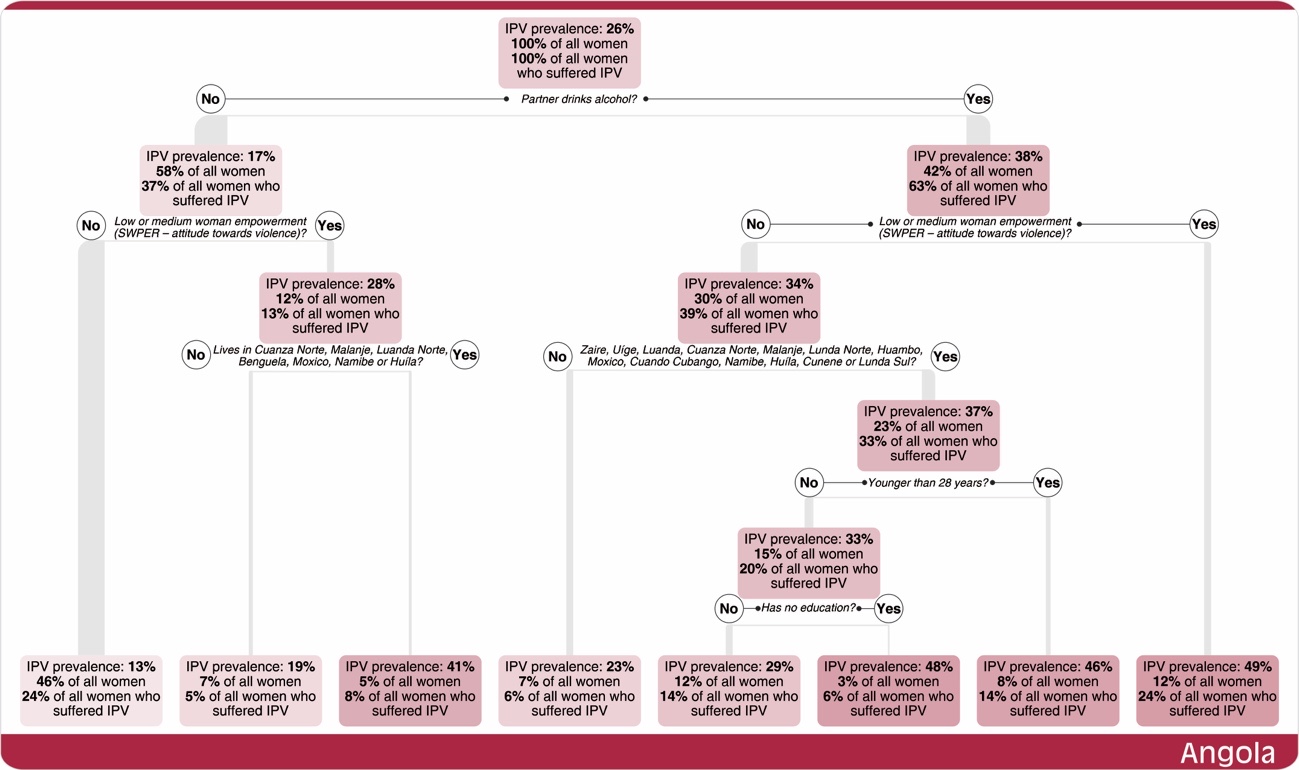


Figure S4 – Angola, 2015: decision tree


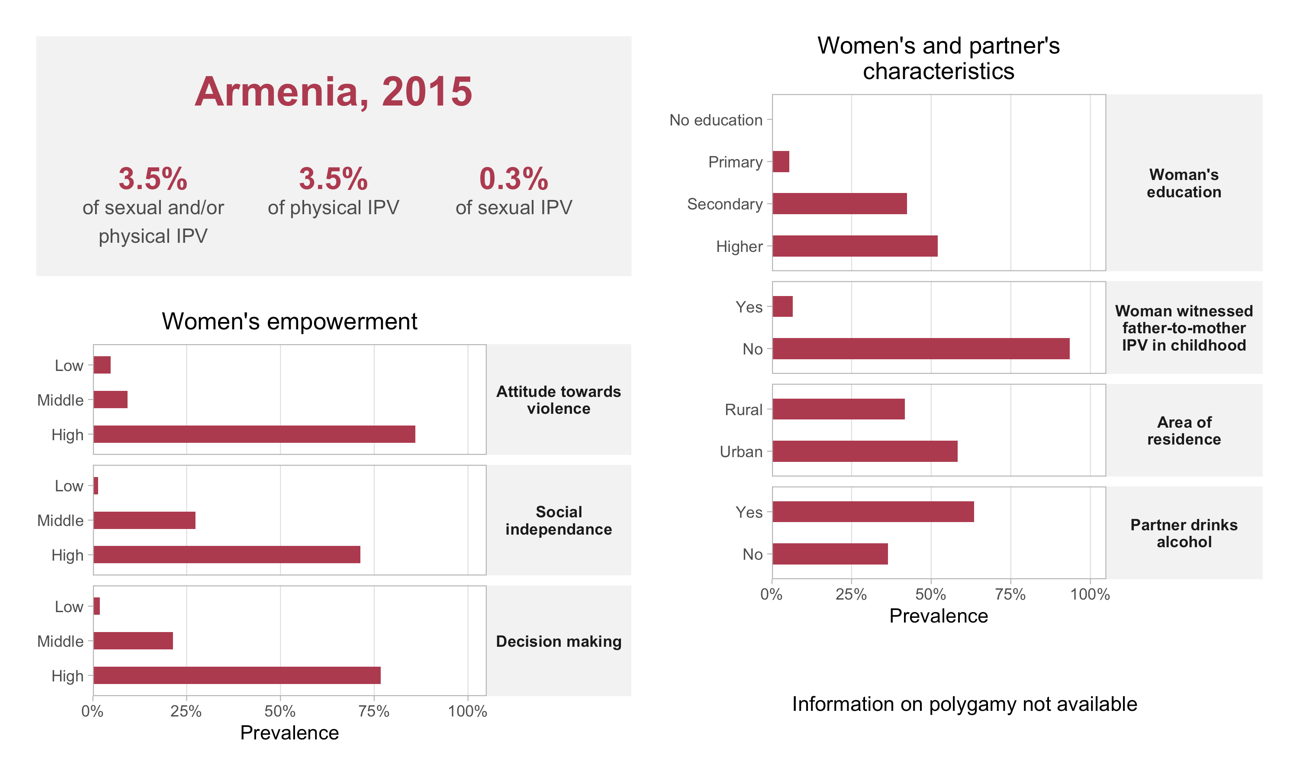
Figure S5 – Armenia, 2015: country profile


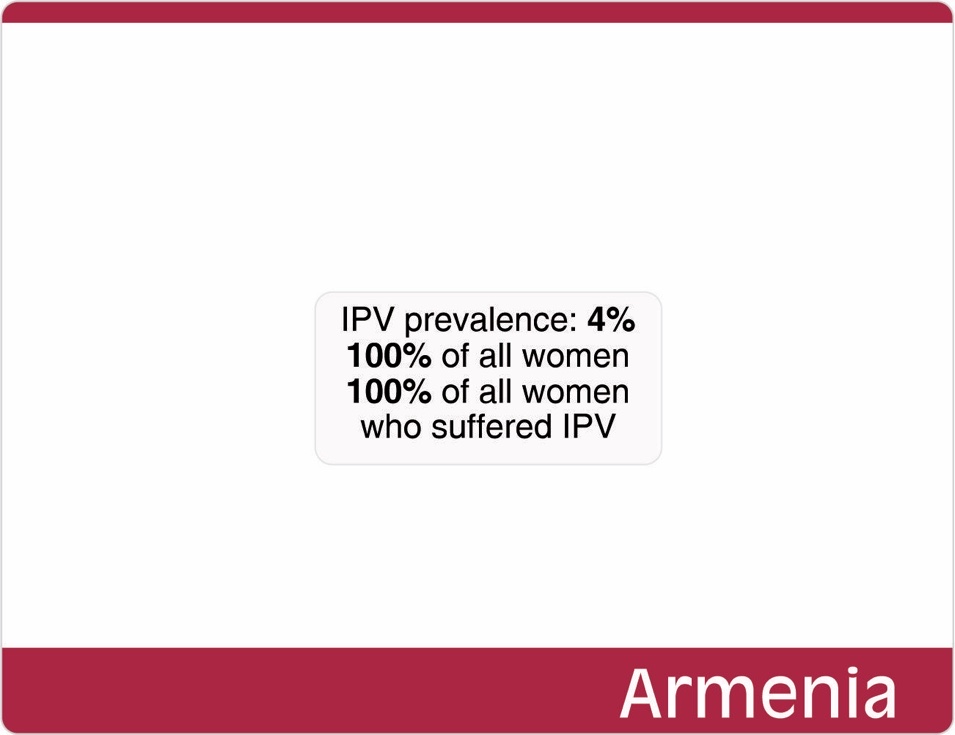


Figure S6 – Armenia, 2015: decision tree


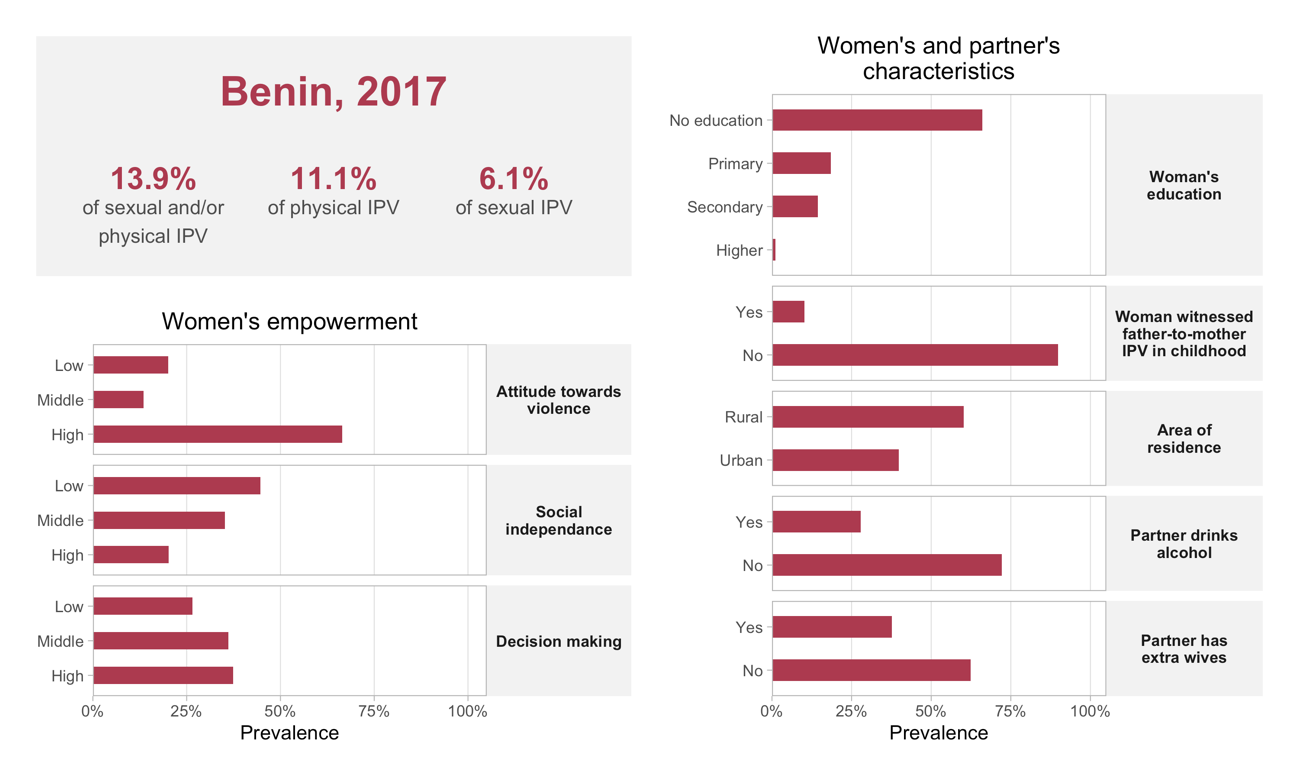
Figure S7 – Benin, 2017: country profile


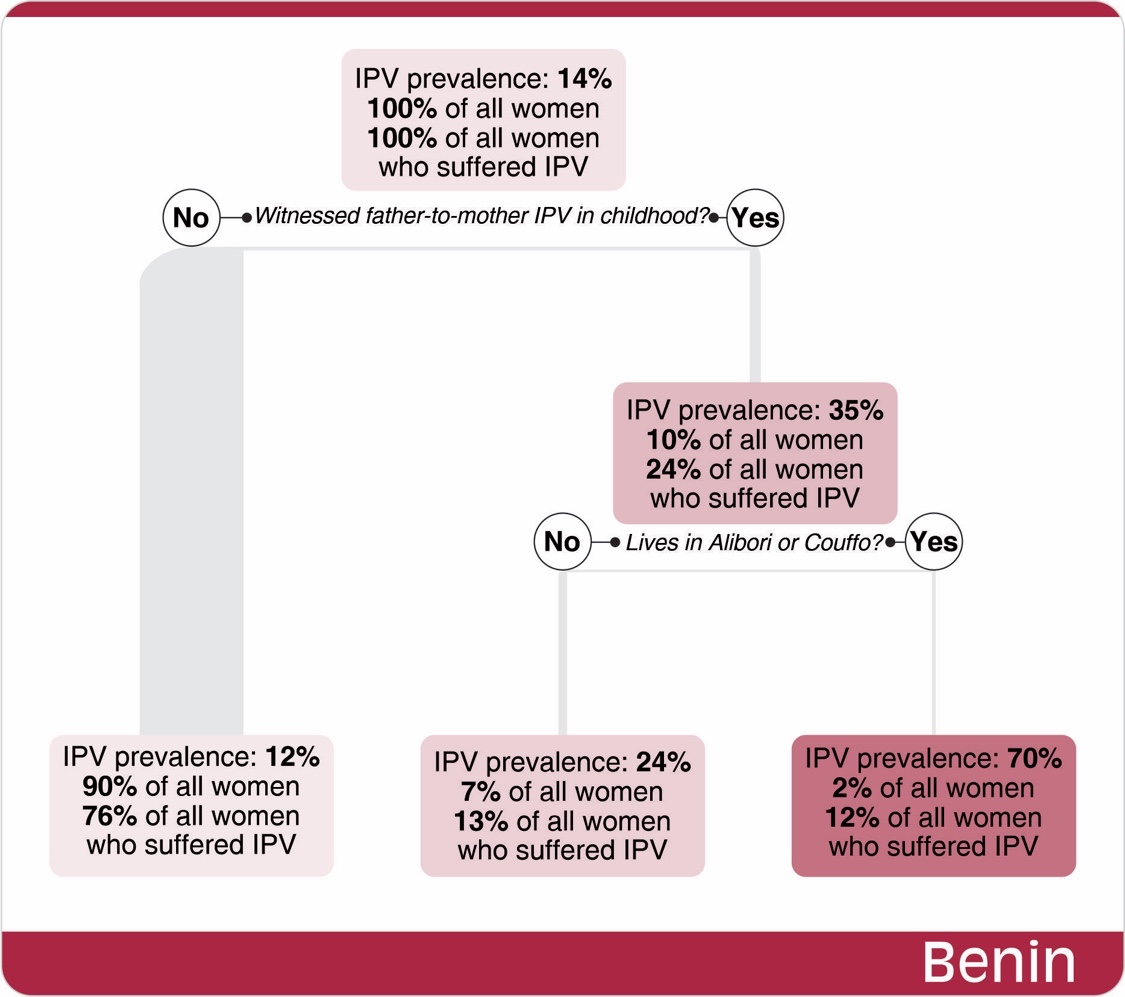


Figure S8 – Benin, 2017: decision tree


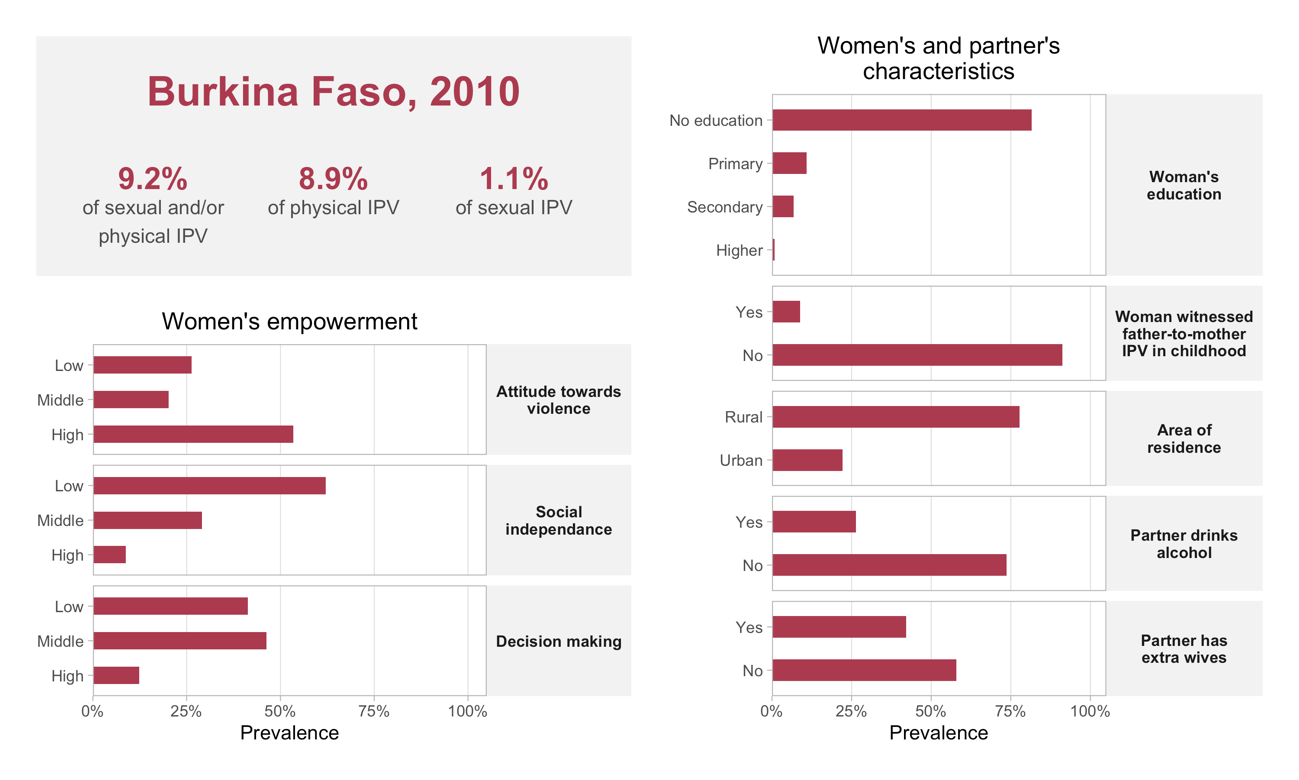
Figure S9 – Burkina Faso, 2010: country profile


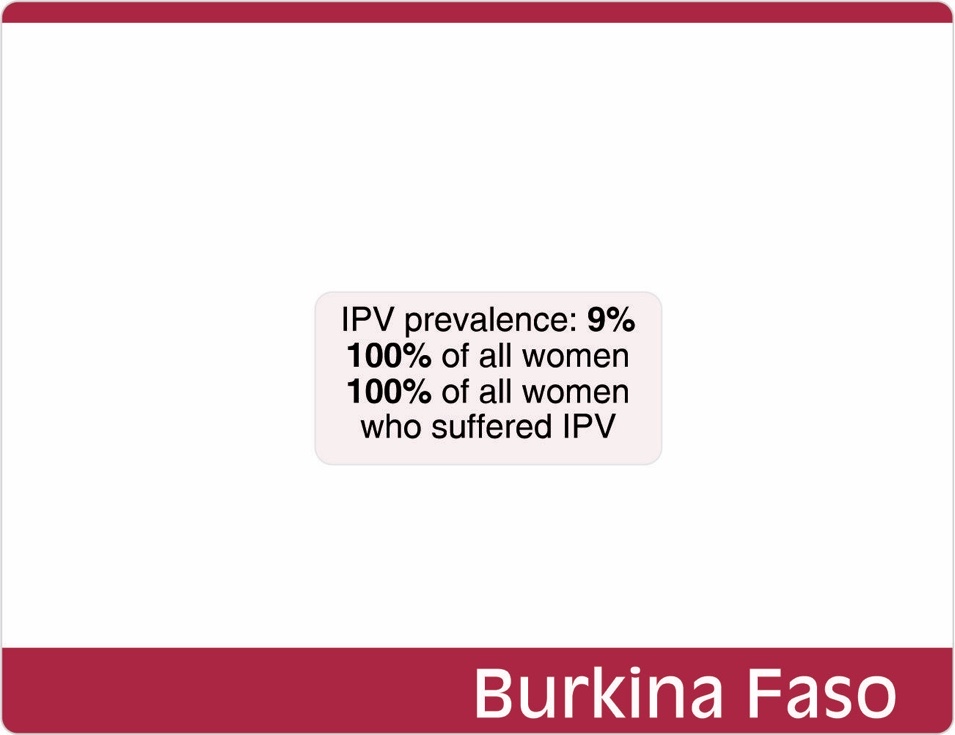


Figure S10 – Burkina Faso, 2010: decision tree


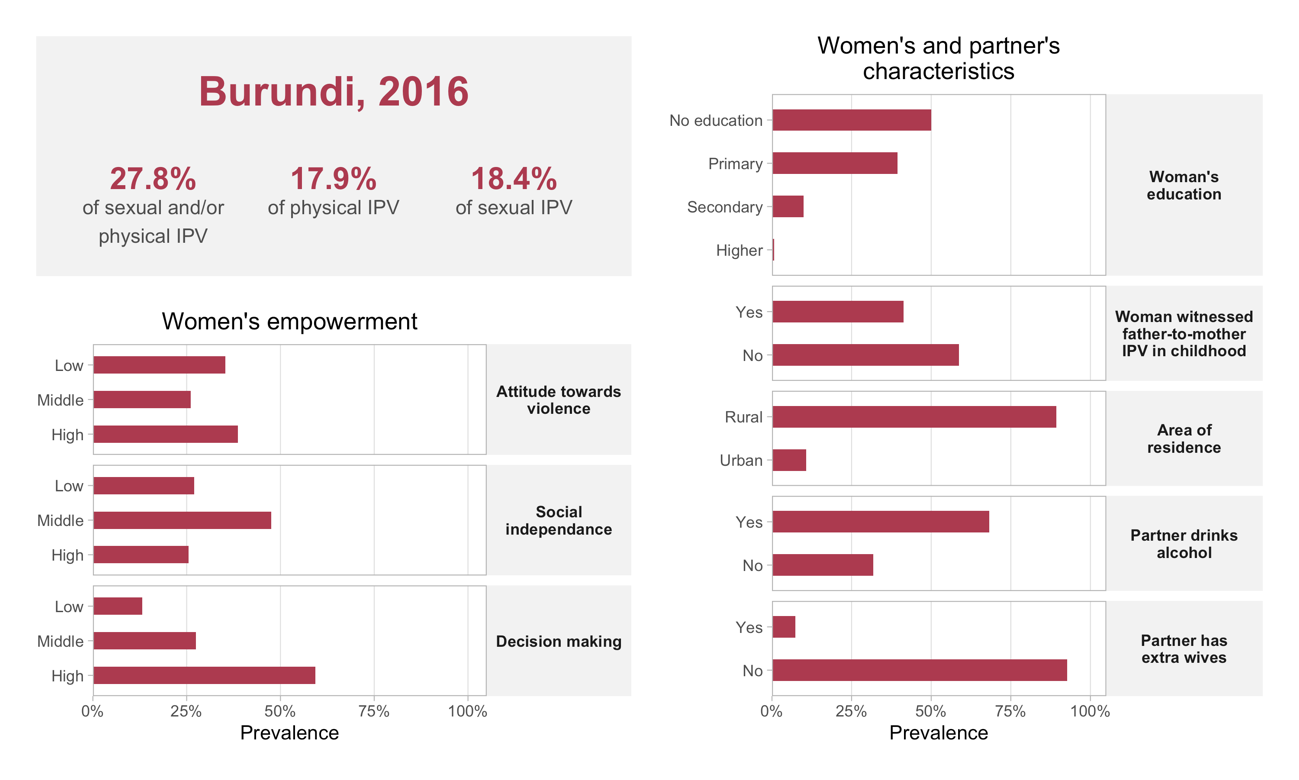
Figure S11 – Burundi, 2016: country profile


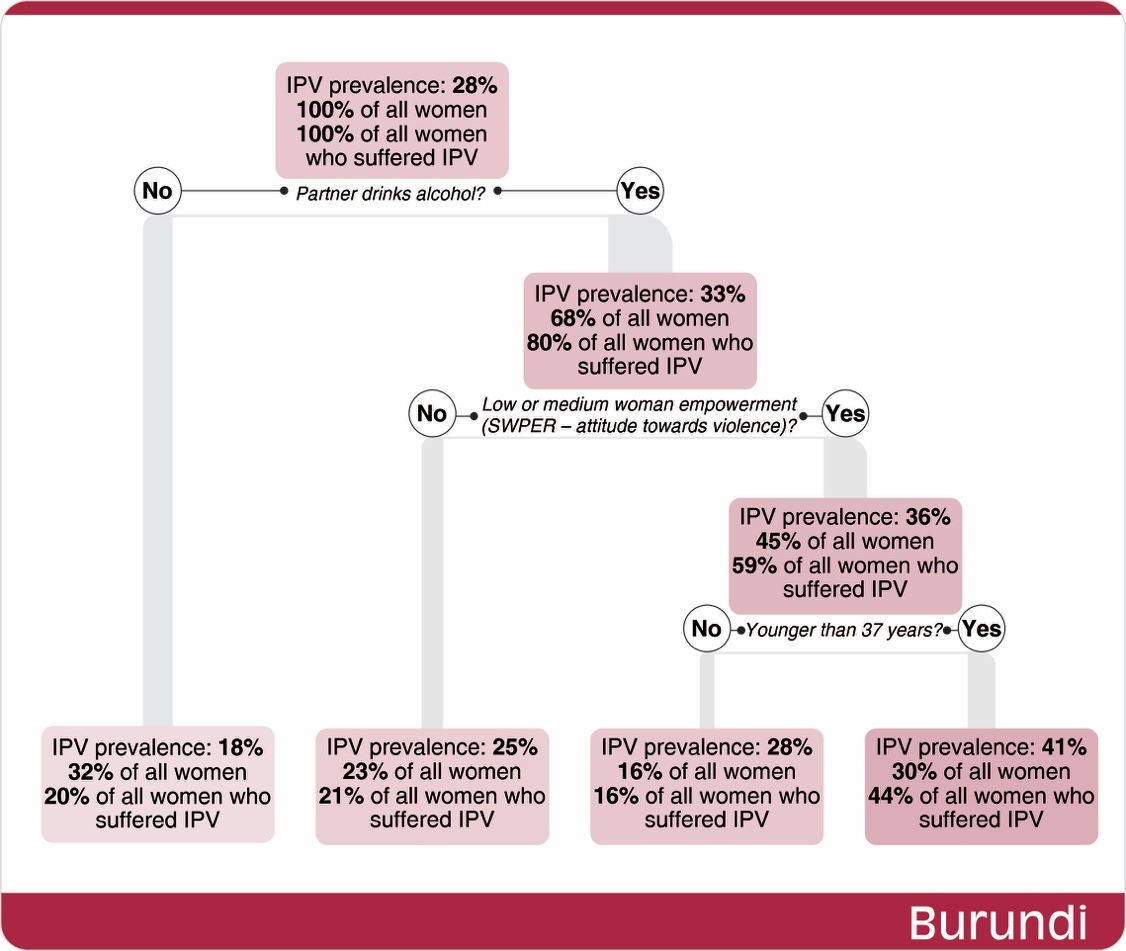


Figure S12 – Burundi, 2016: decision tree


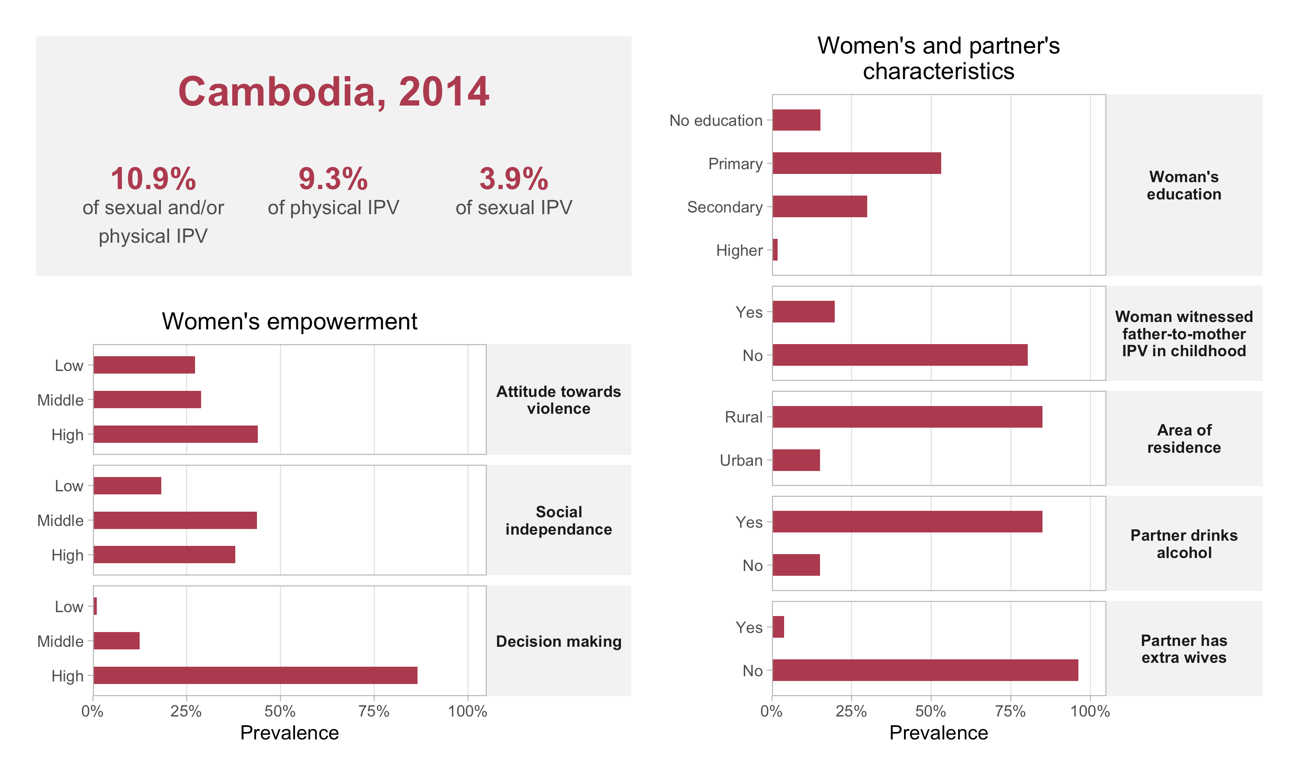
Figure S13 – Cambodia, 2014: country profile


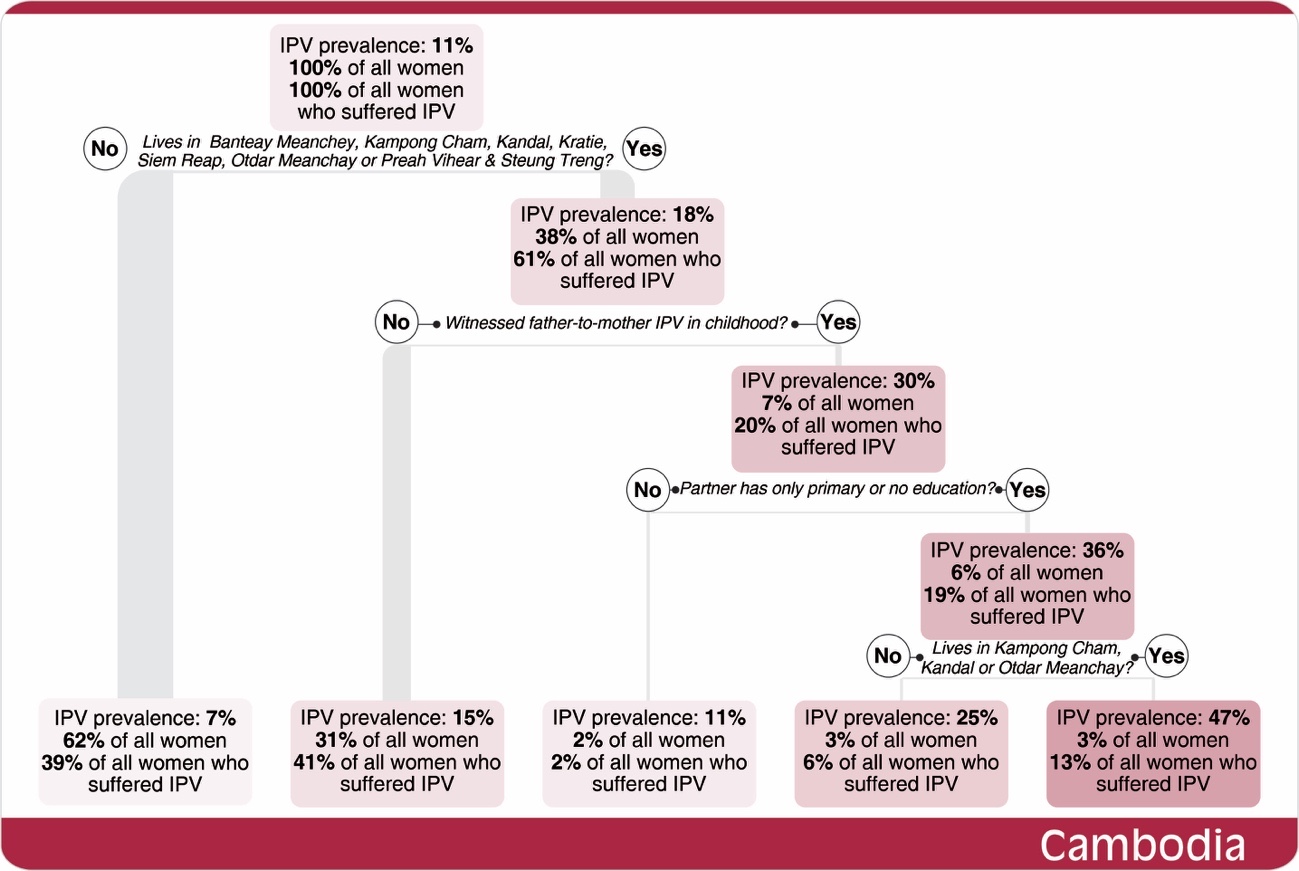


Figure S14 – Cambodia, 2014: decision tree


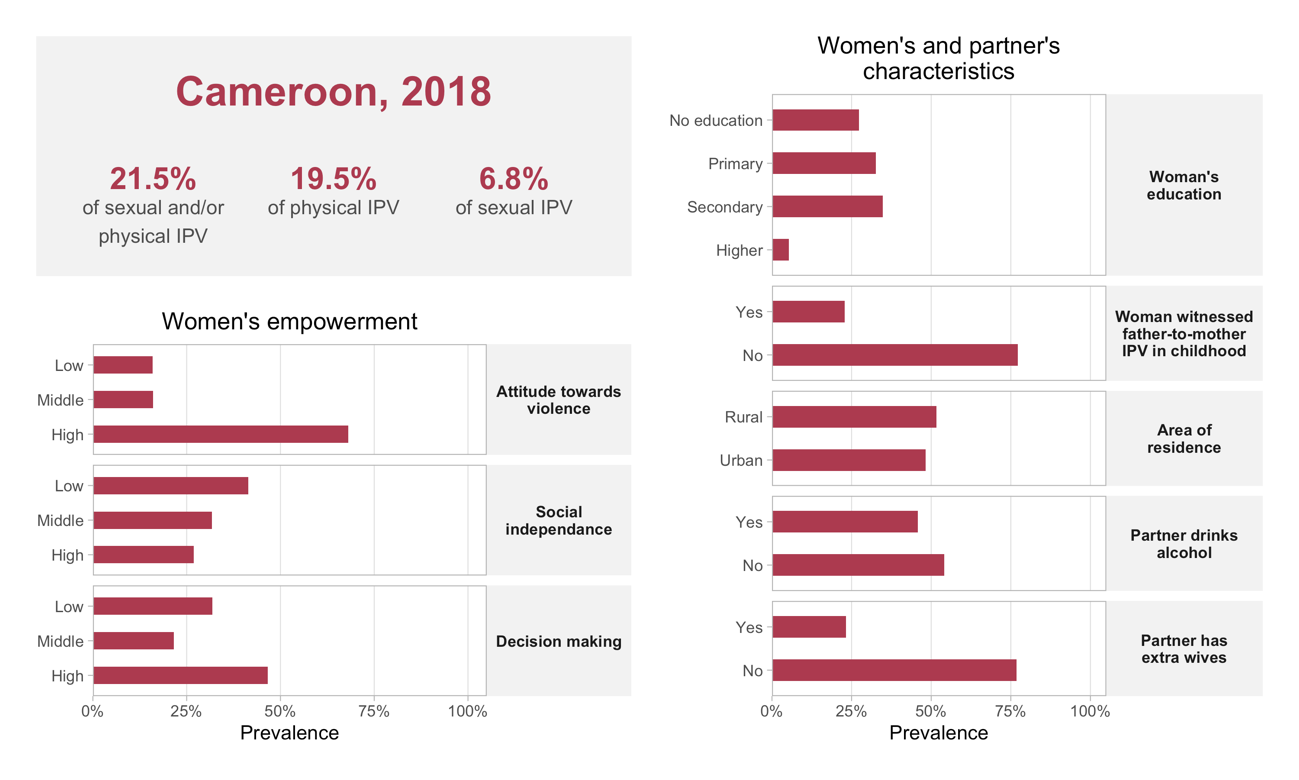
Figure S15 – Cameroon, 2018: country profile


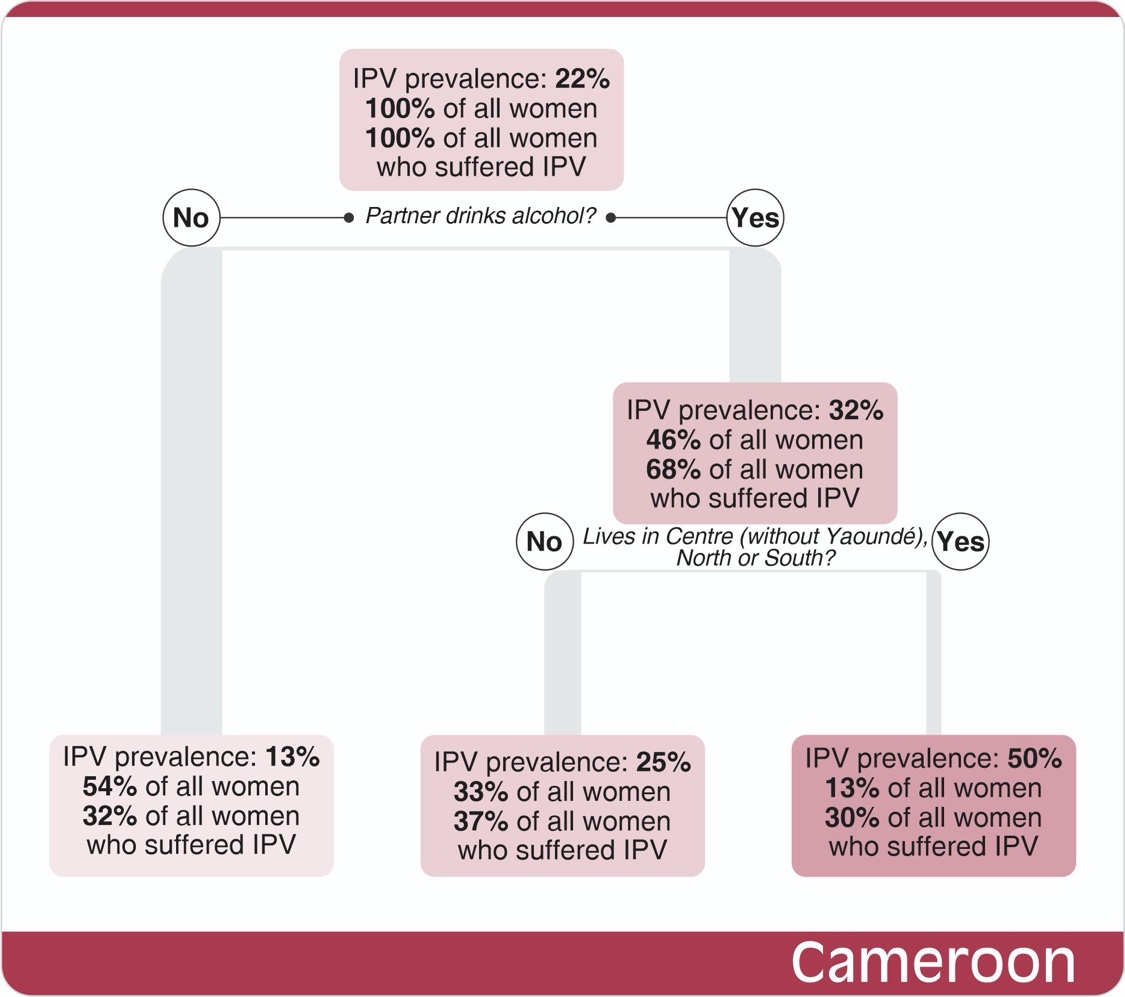


Figure S16 – Cameroon, 2018: decision tree


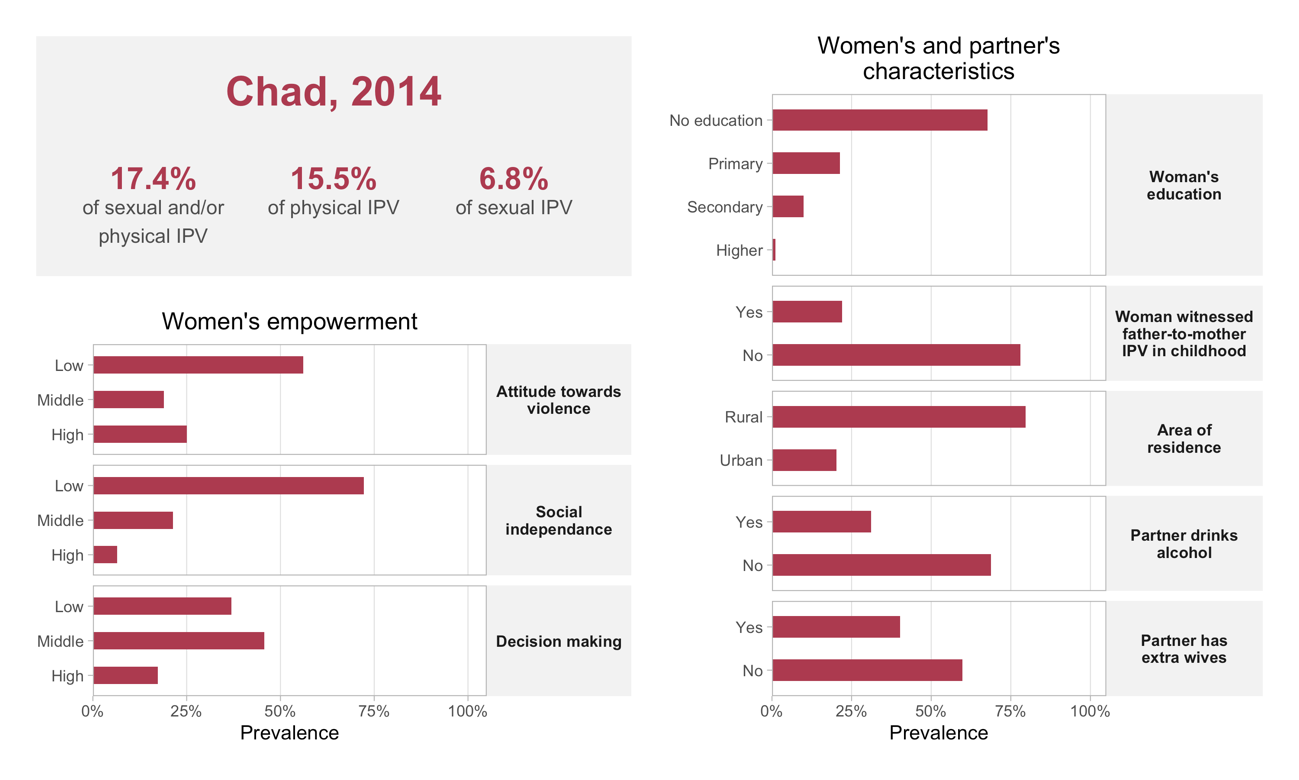
Figure S17 – Chad, 2014: country profile


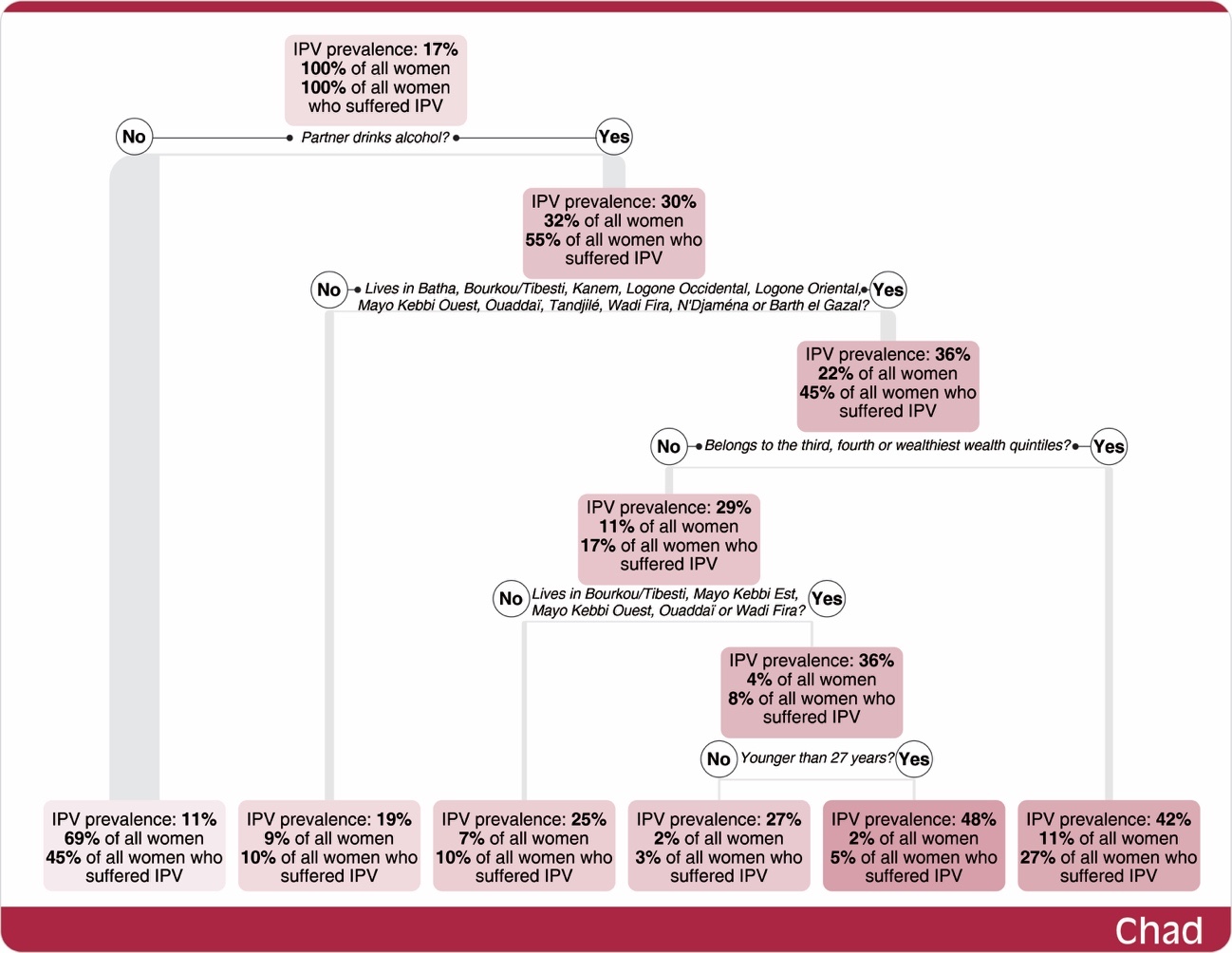


Figure S18 – Chad, 2014: decision tree


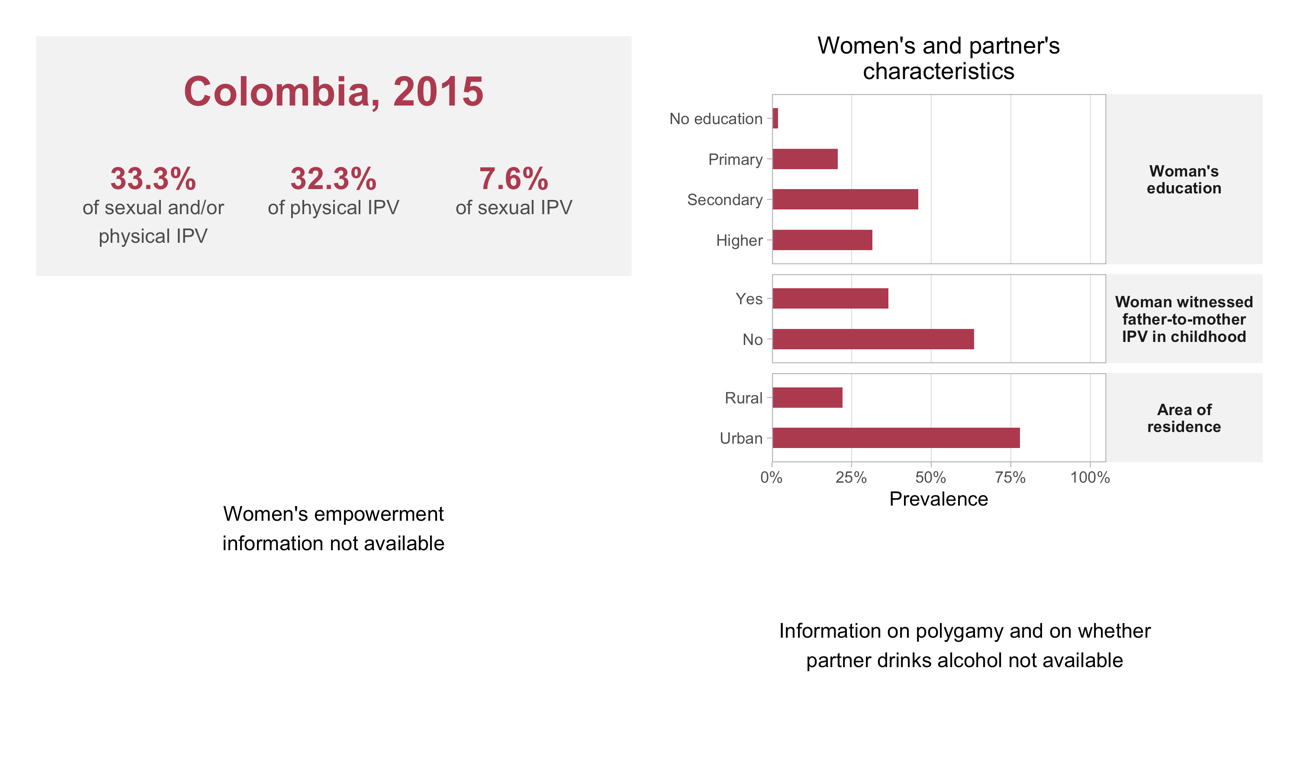
Figure S19 – Colombia, 2015: country profile


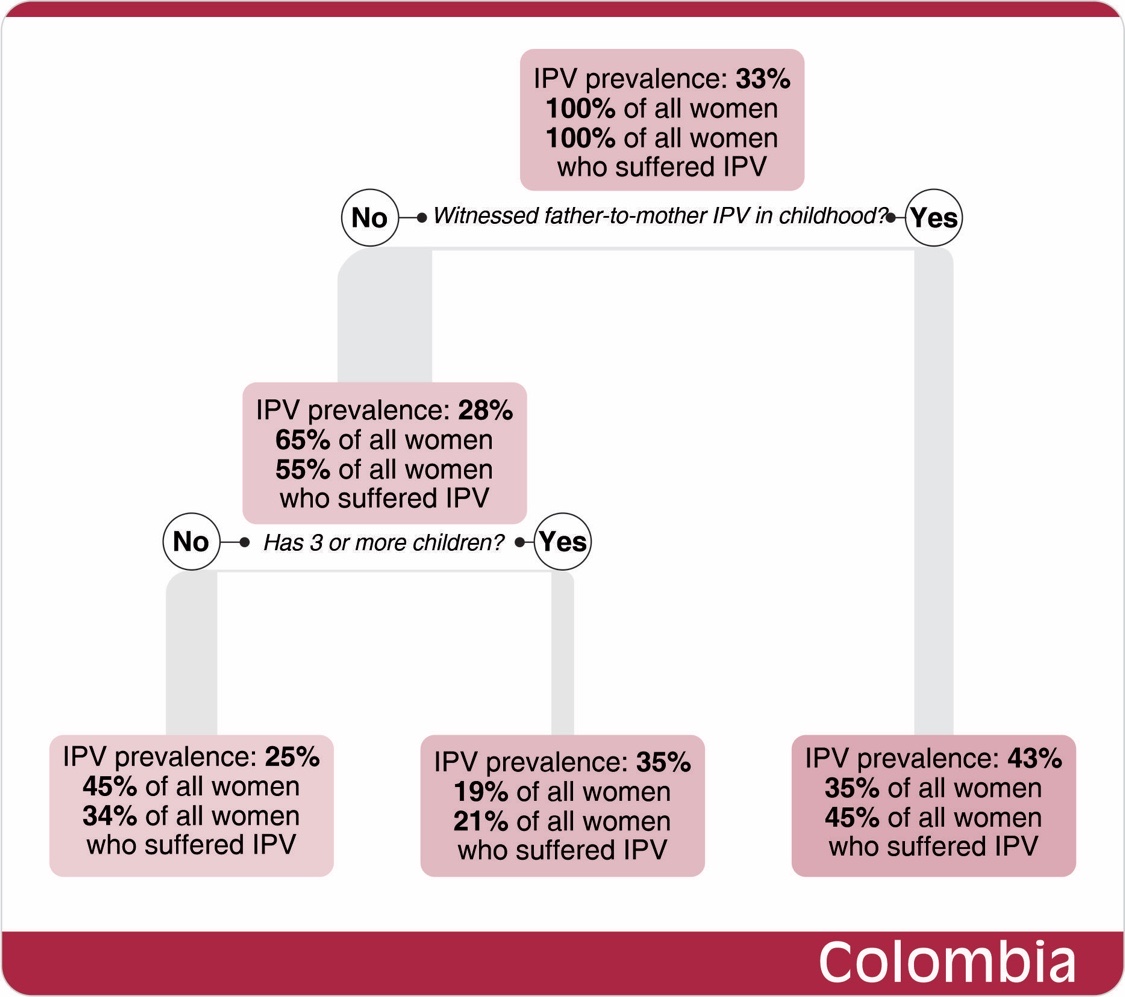


Figure S20 – Colombia, 2015: decision tree


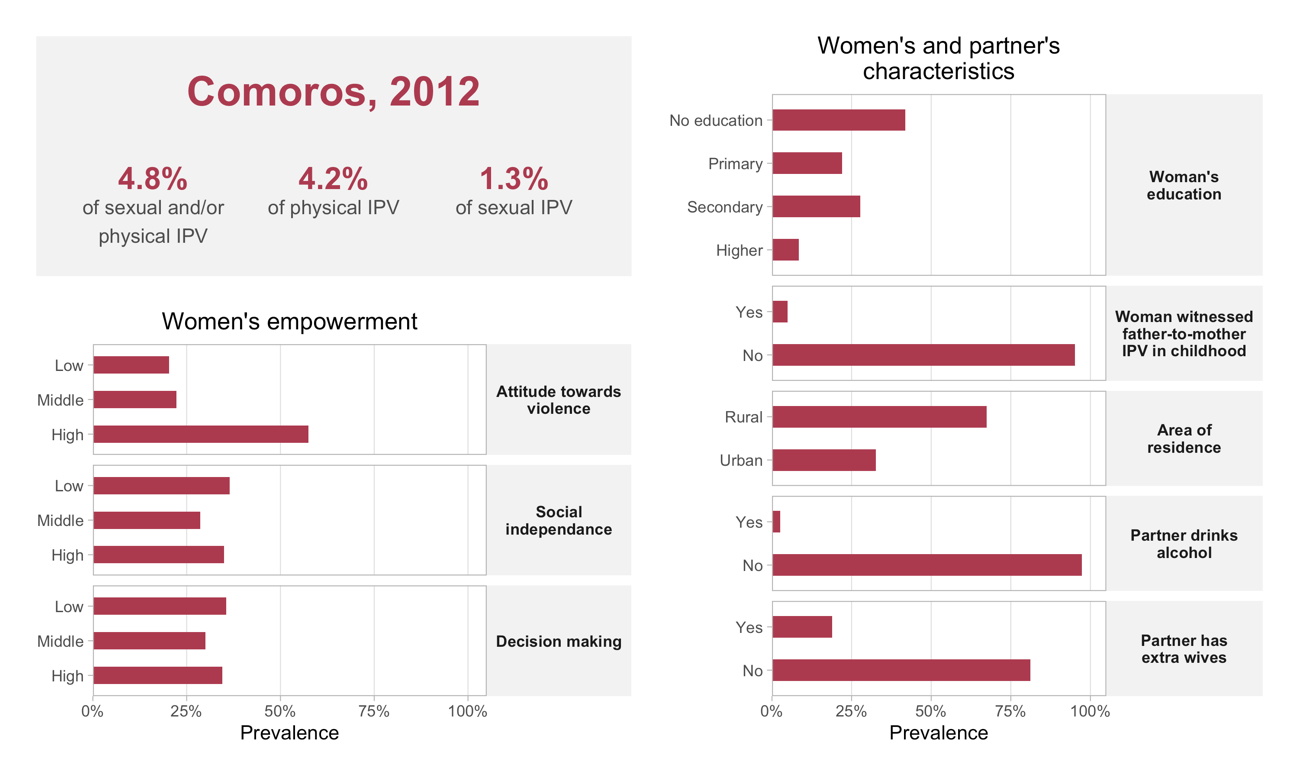
Figure S21 – Comoros, 2012: country profile


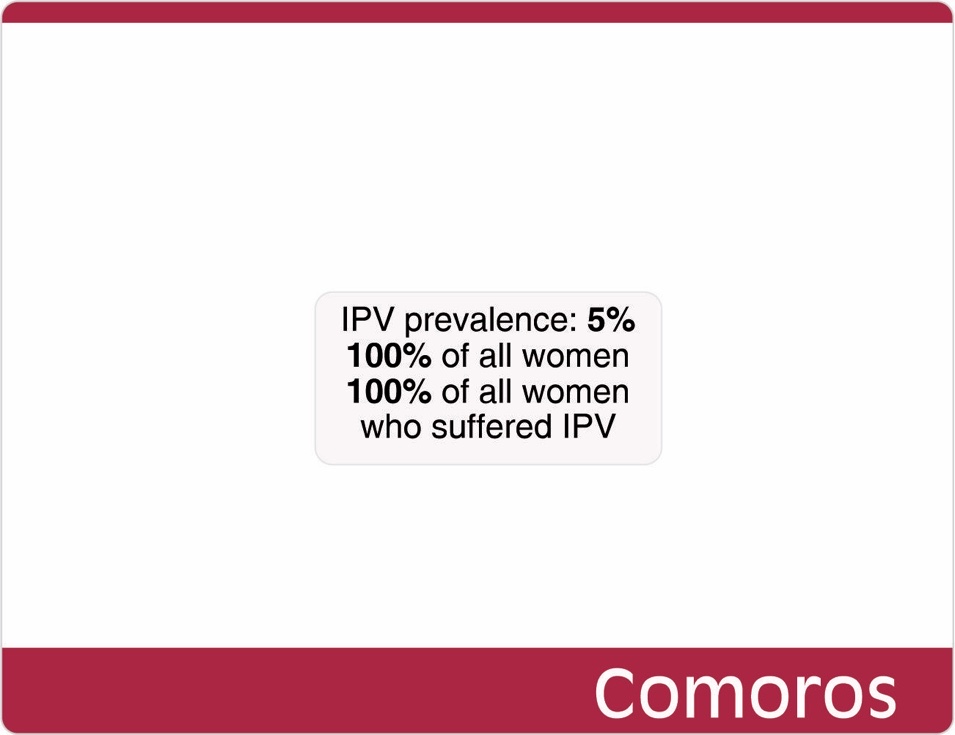


Figure S22 – Comoros, 2012: decision tree


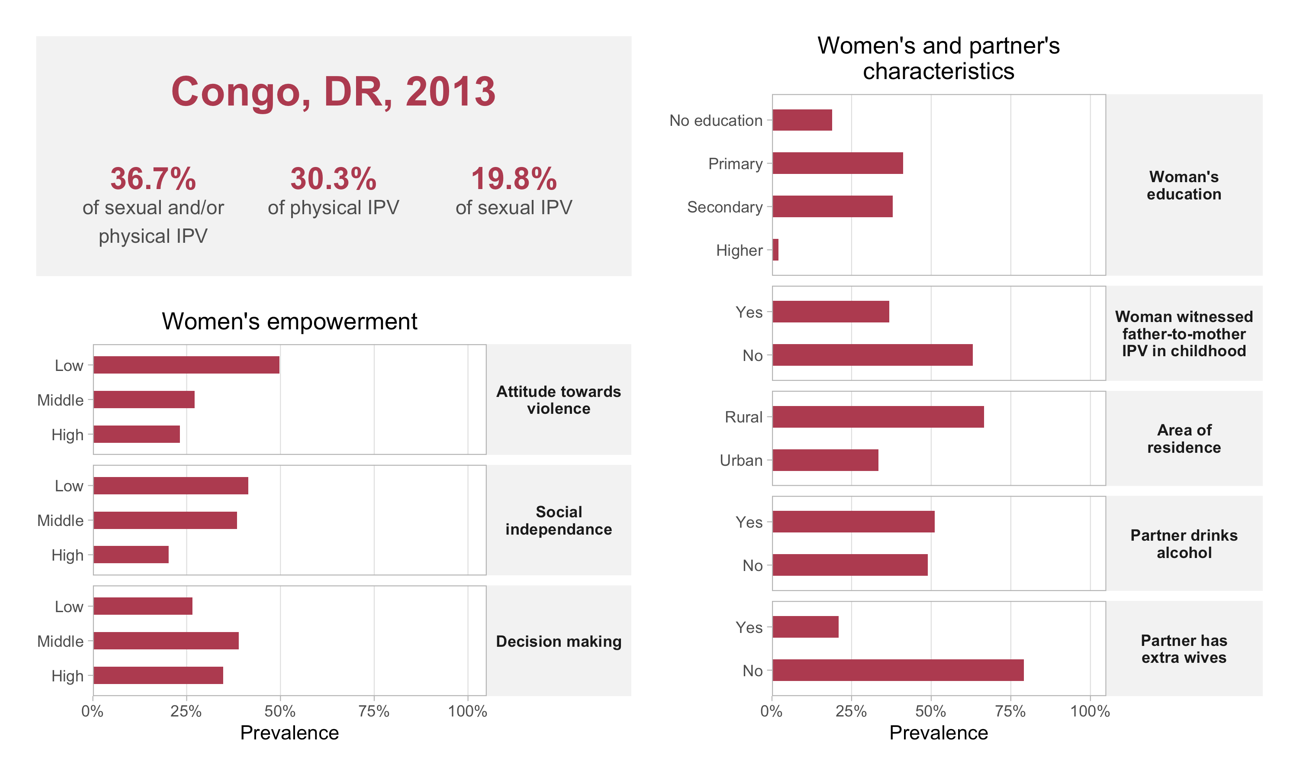
Figure S23 – Congo, DR, 2013: country profile


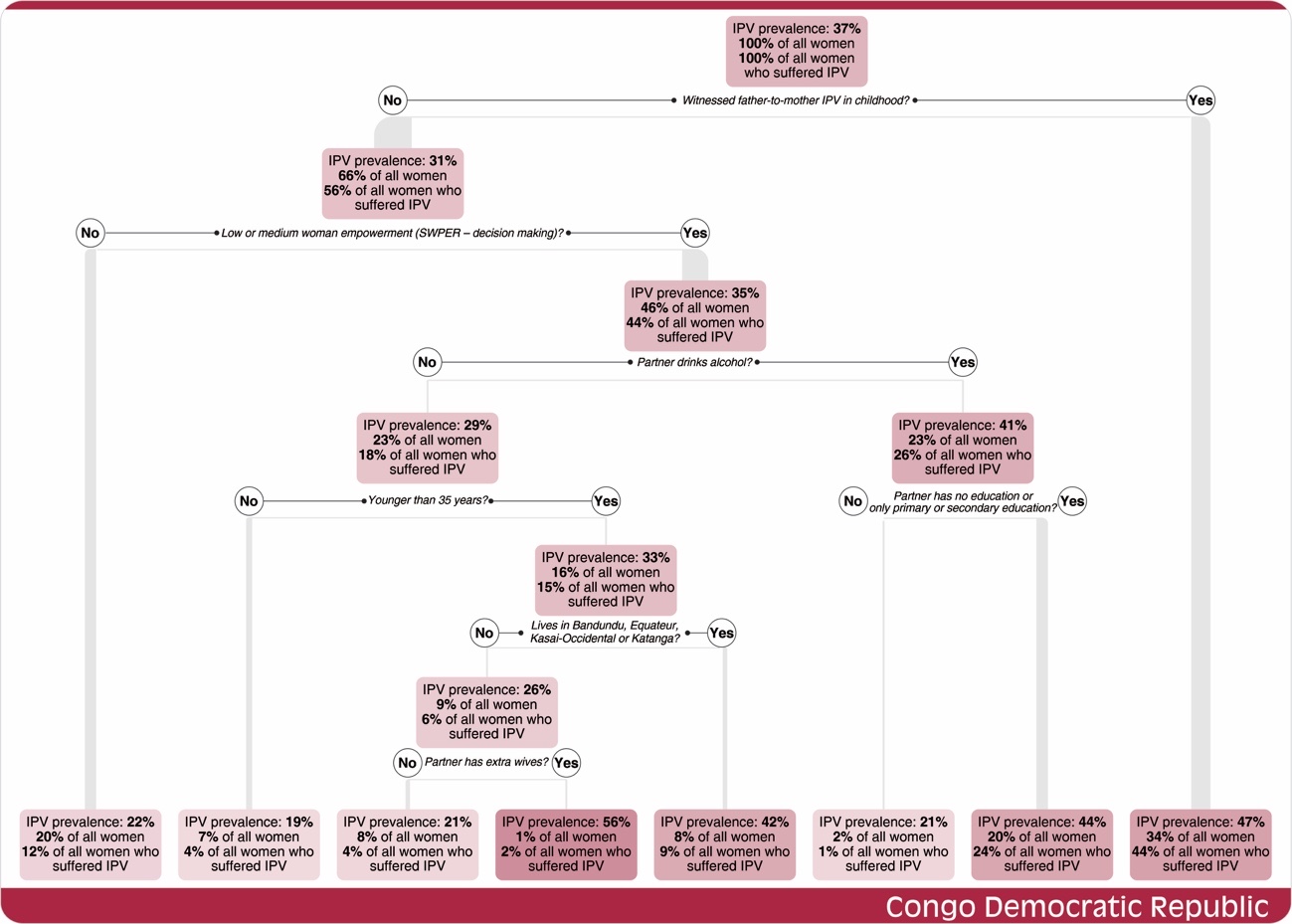


Figure S24 – Congo, DR, 2013: decision tree


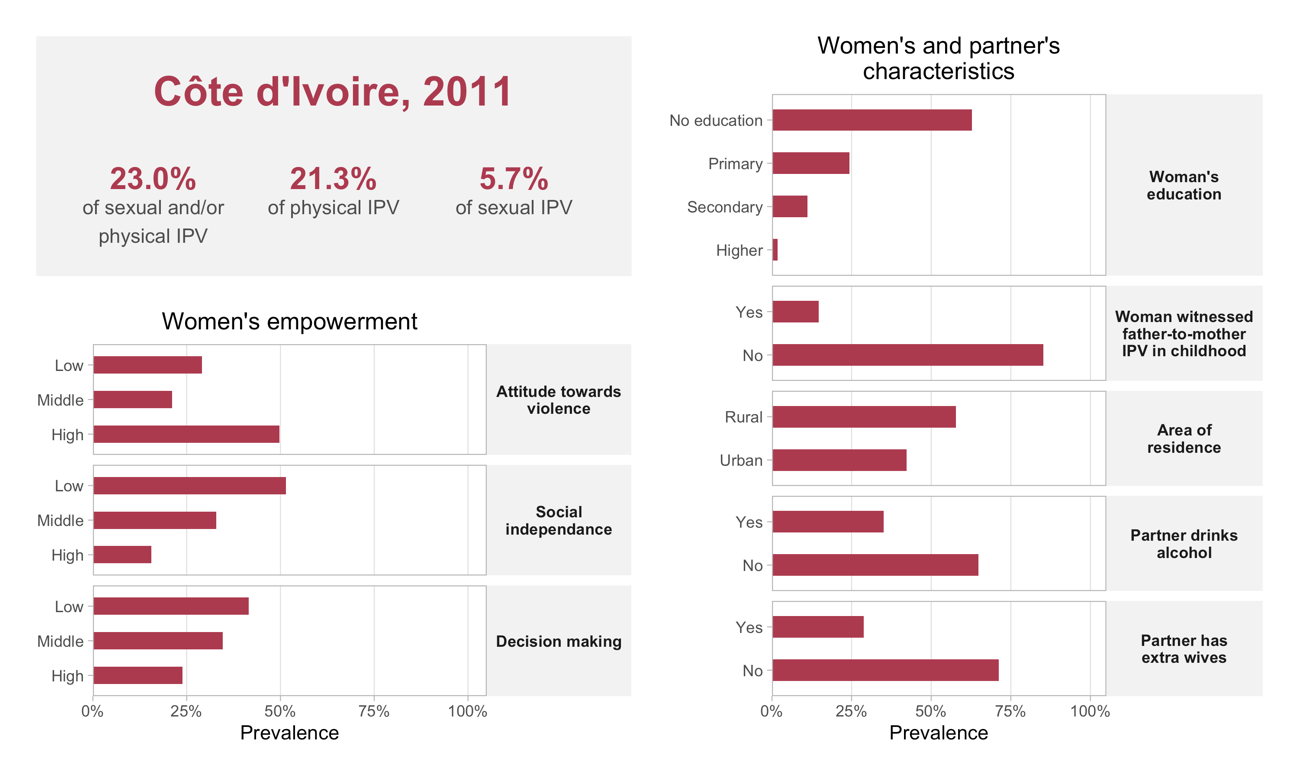
Figure S25 – Côte d’Ivoire, 2011: country profile


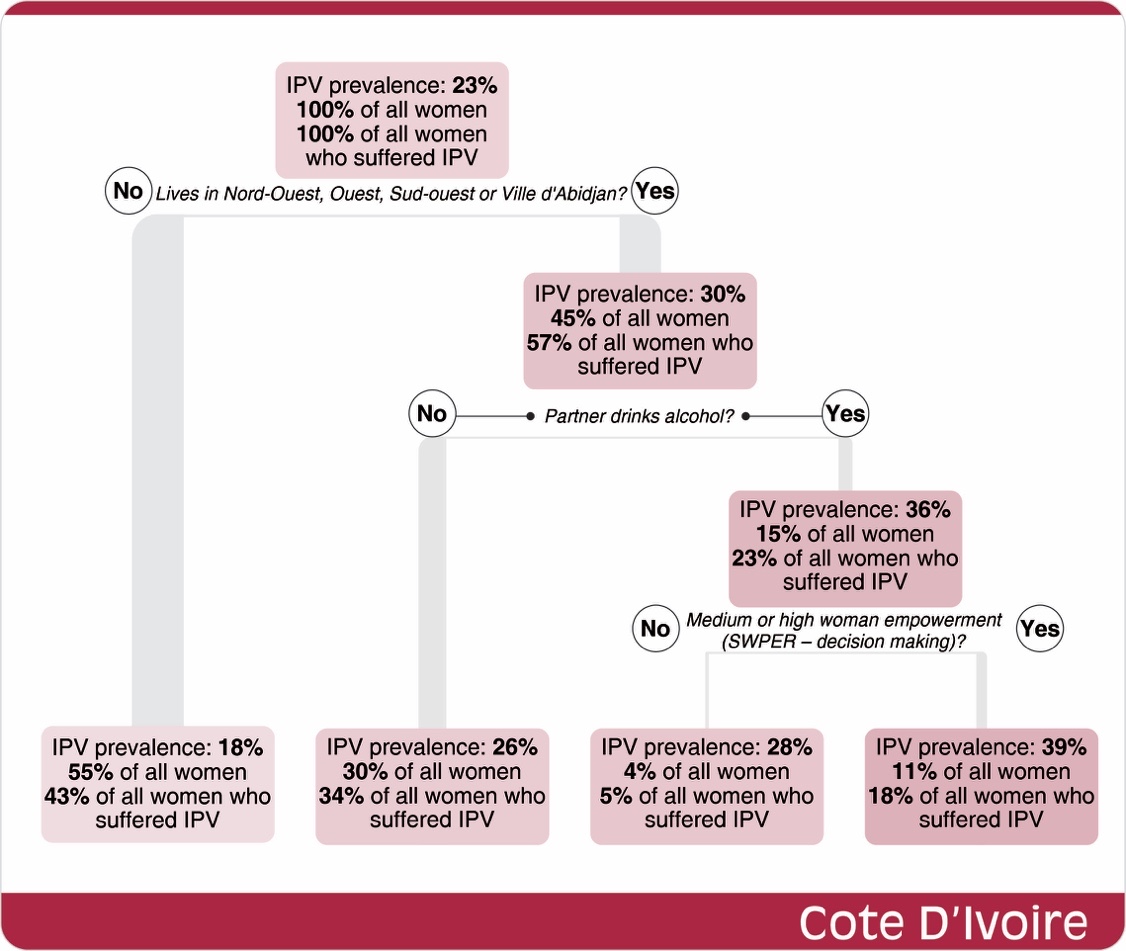


Figure S26 – Côte d’Ivoire, 2011: decision tree


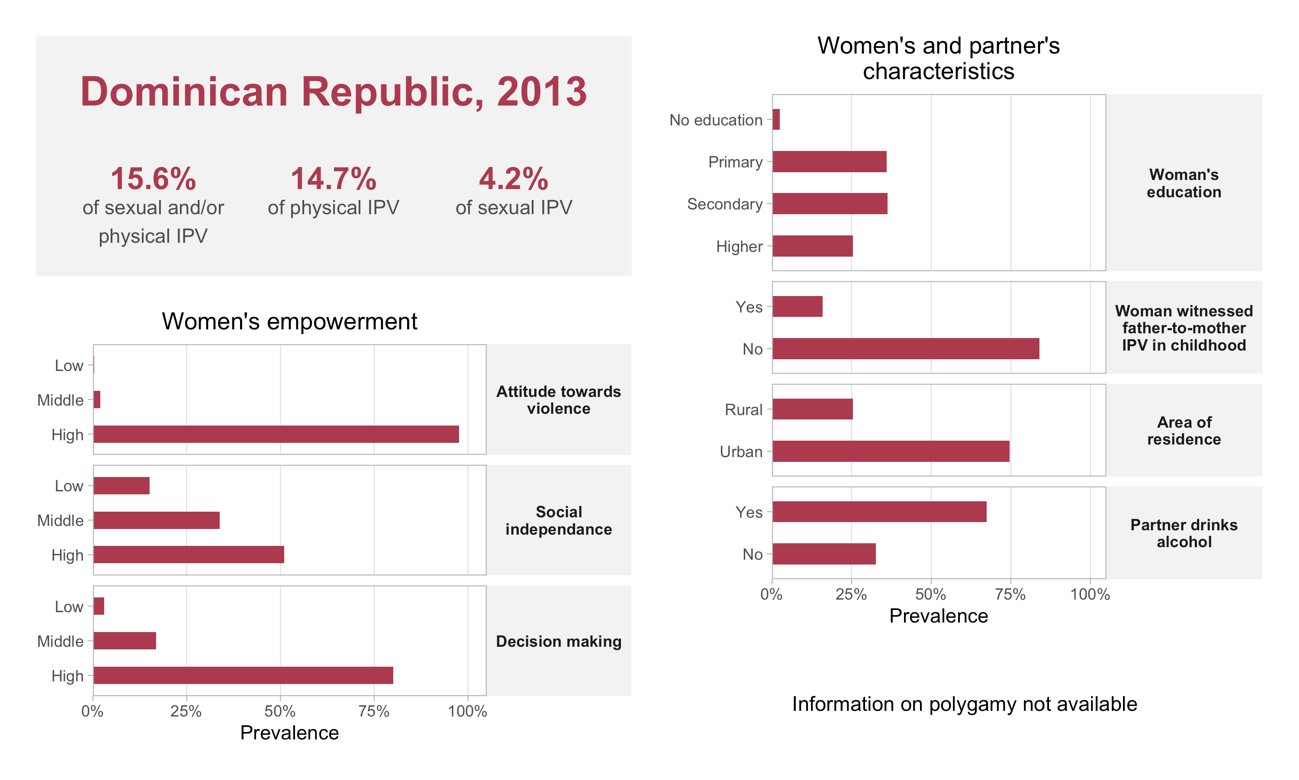
Figure S27 – Dominican Republic, 2013: country profile


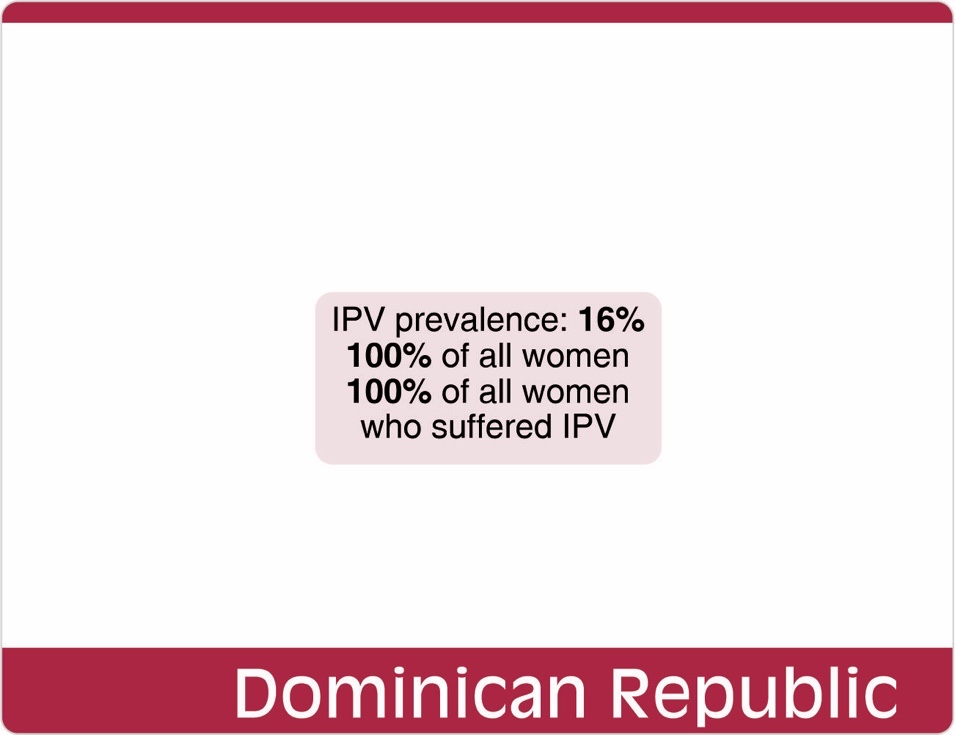


Figure S28 – Dominican Republic, 2013: decision tree


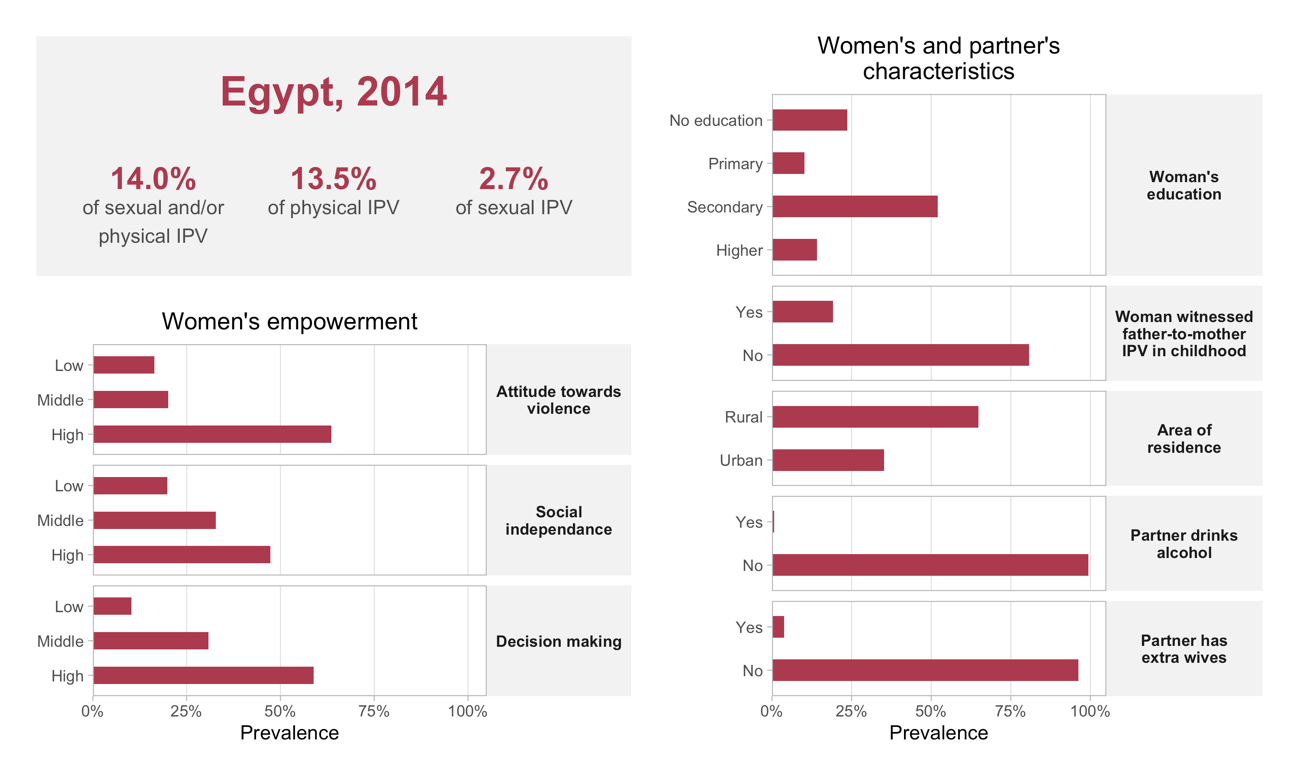
Figure S29 – Egypt, 2014: country profile


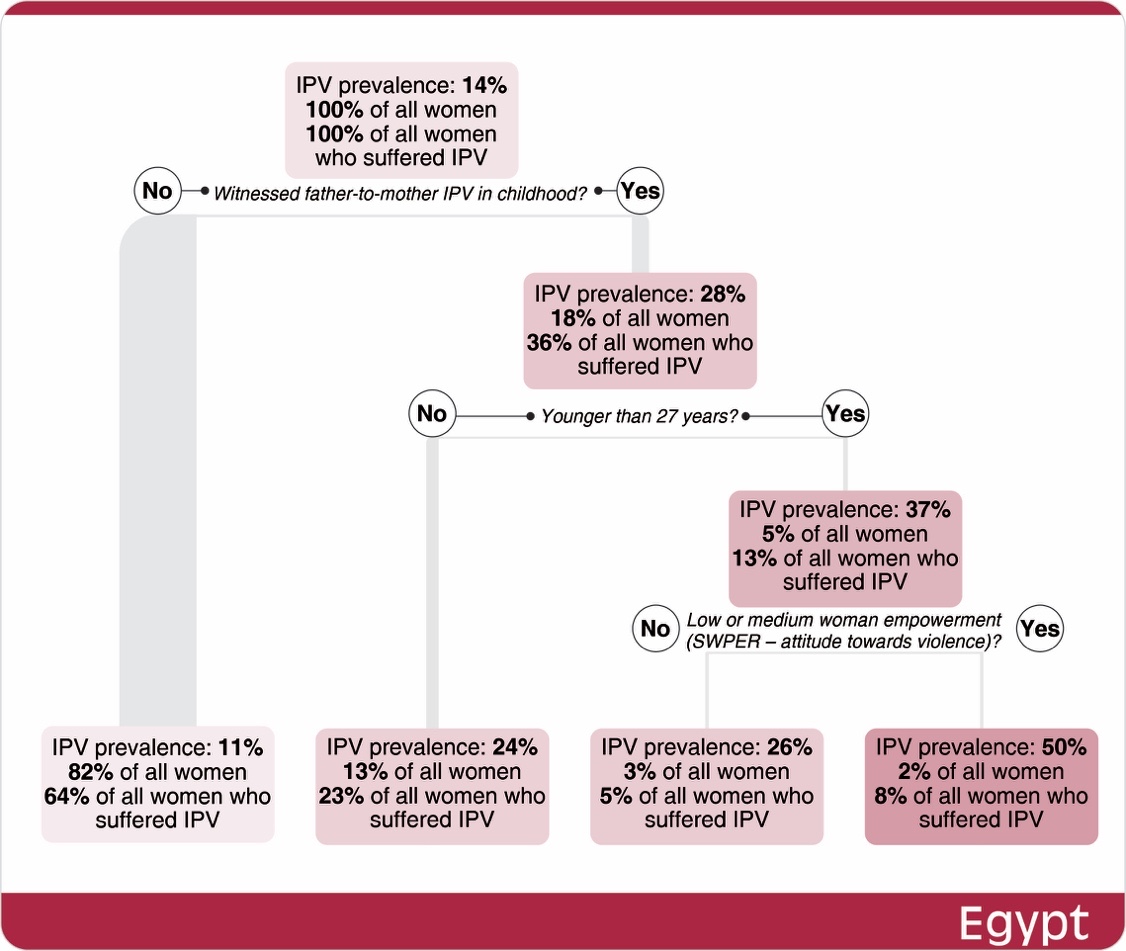


Figure S30 – Egypt, 2014: decision tree


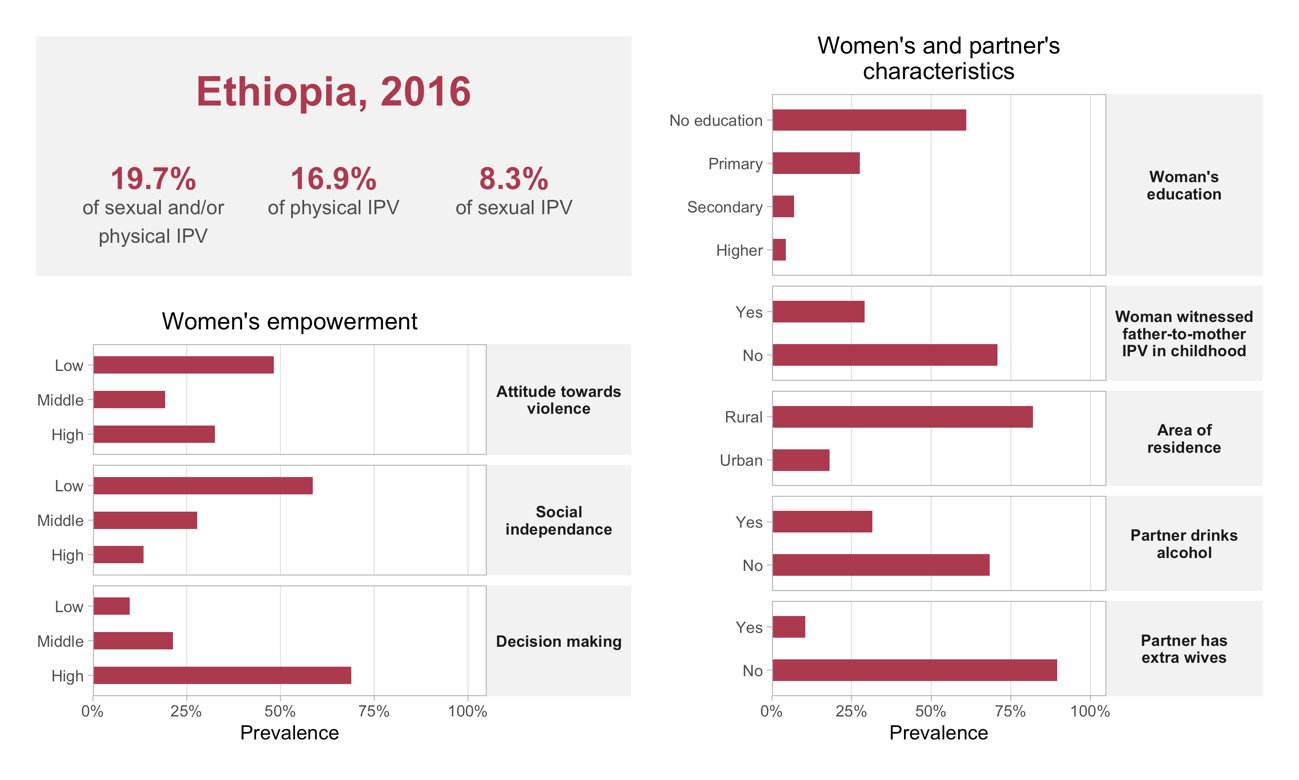
Figure S31 – Ethiopia, 2016: country profile


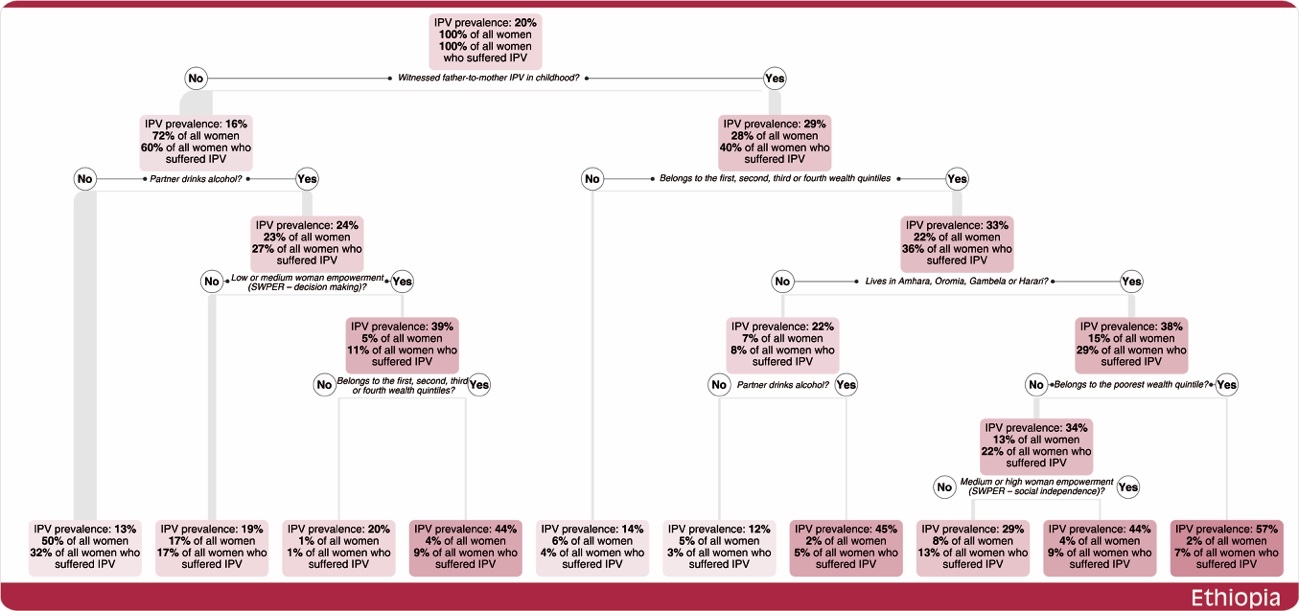


Figure S32 – Ethiopia, 2016: decision tree


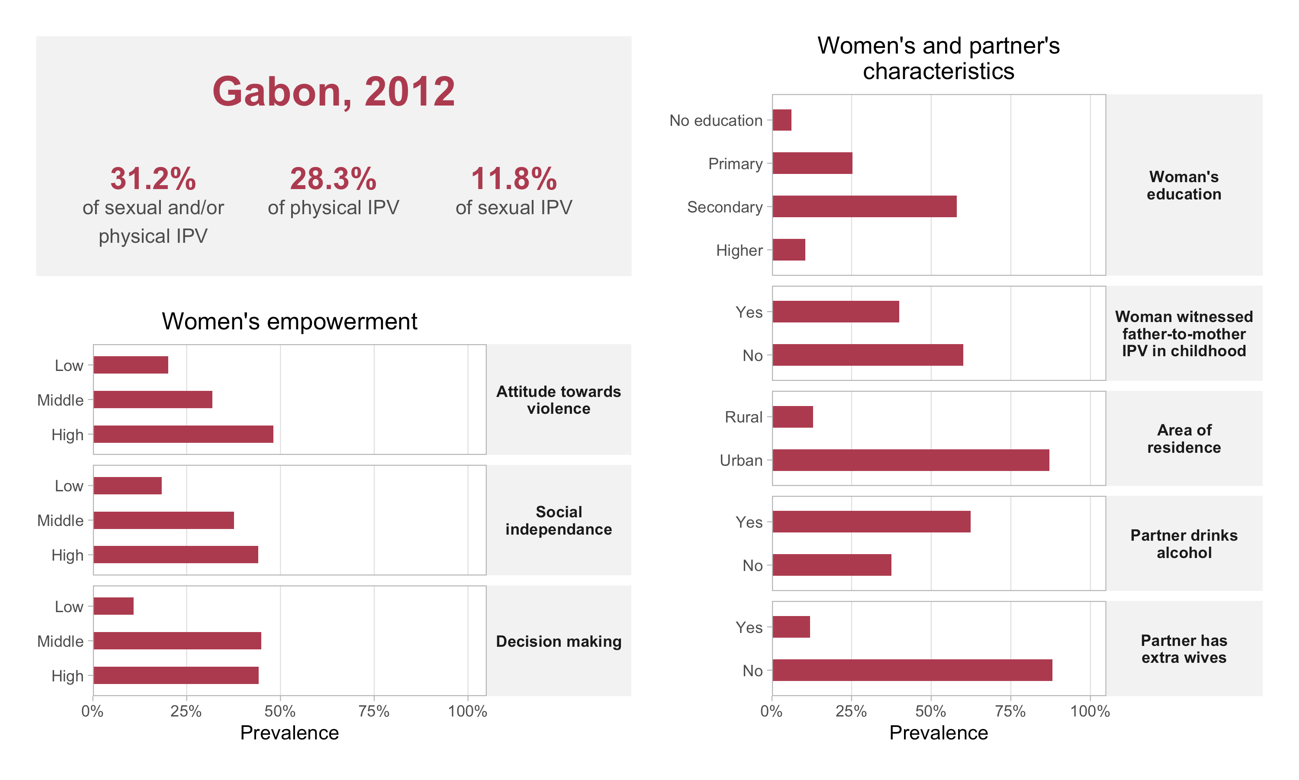
Figure S33 – Gabon, 2012: country profile


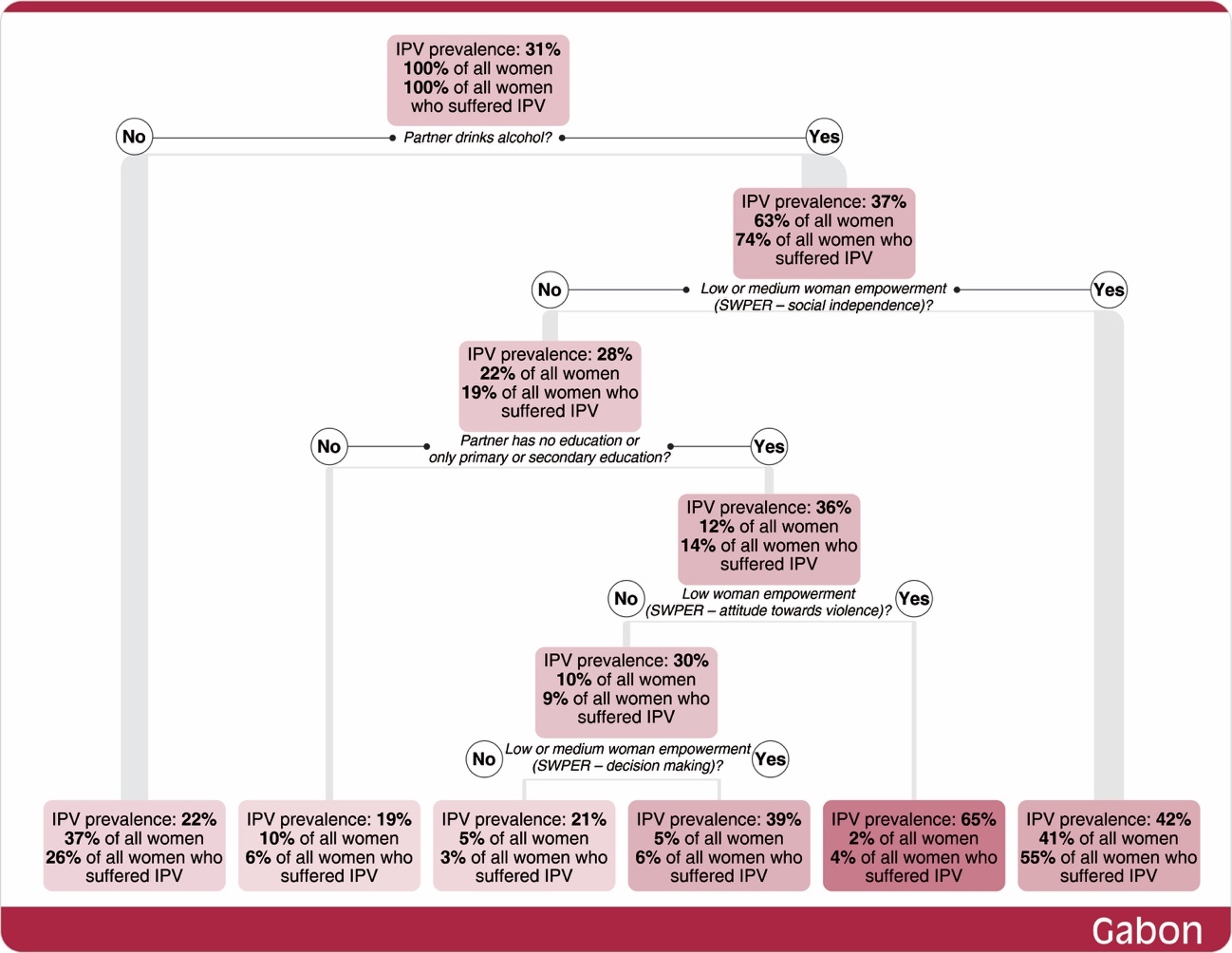


Figure S34 – Gabon, 2012: decision tree


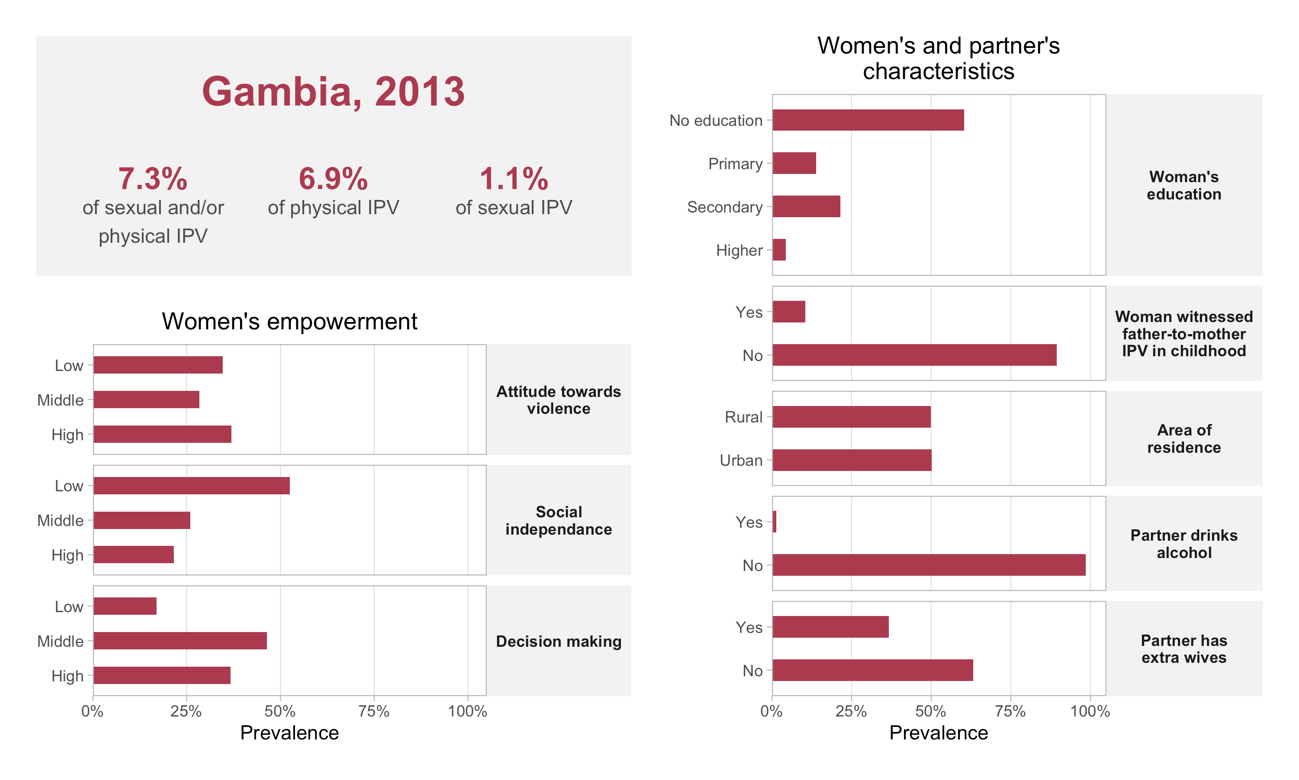
Figure S35 – Gambia, 2013: country profile


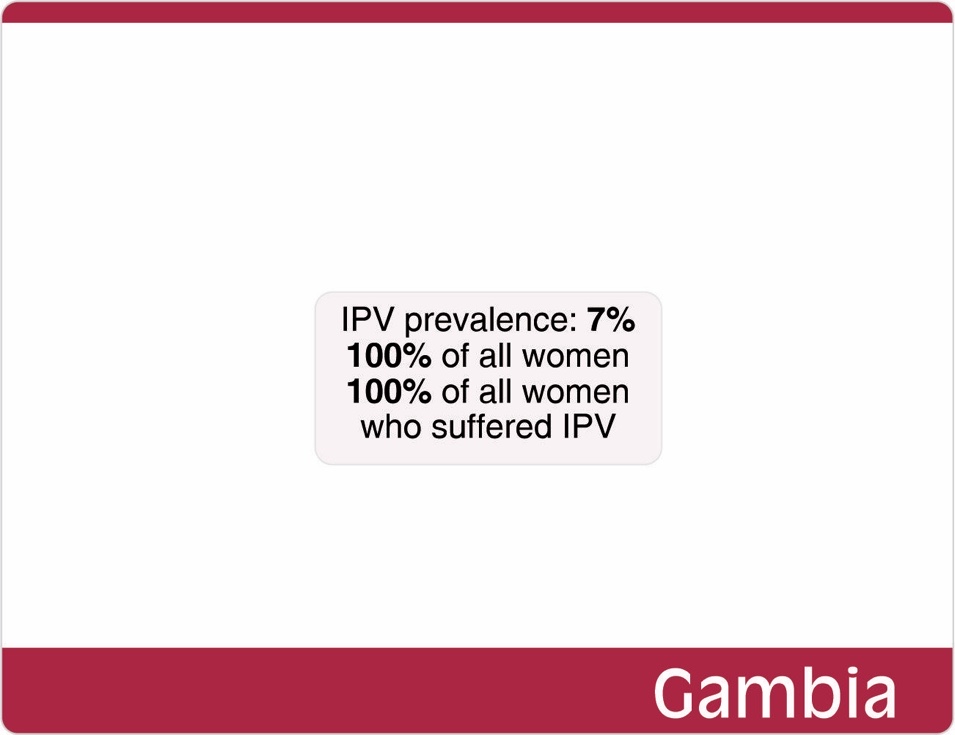


Figure S36 – Gambia, 2013: decision tree


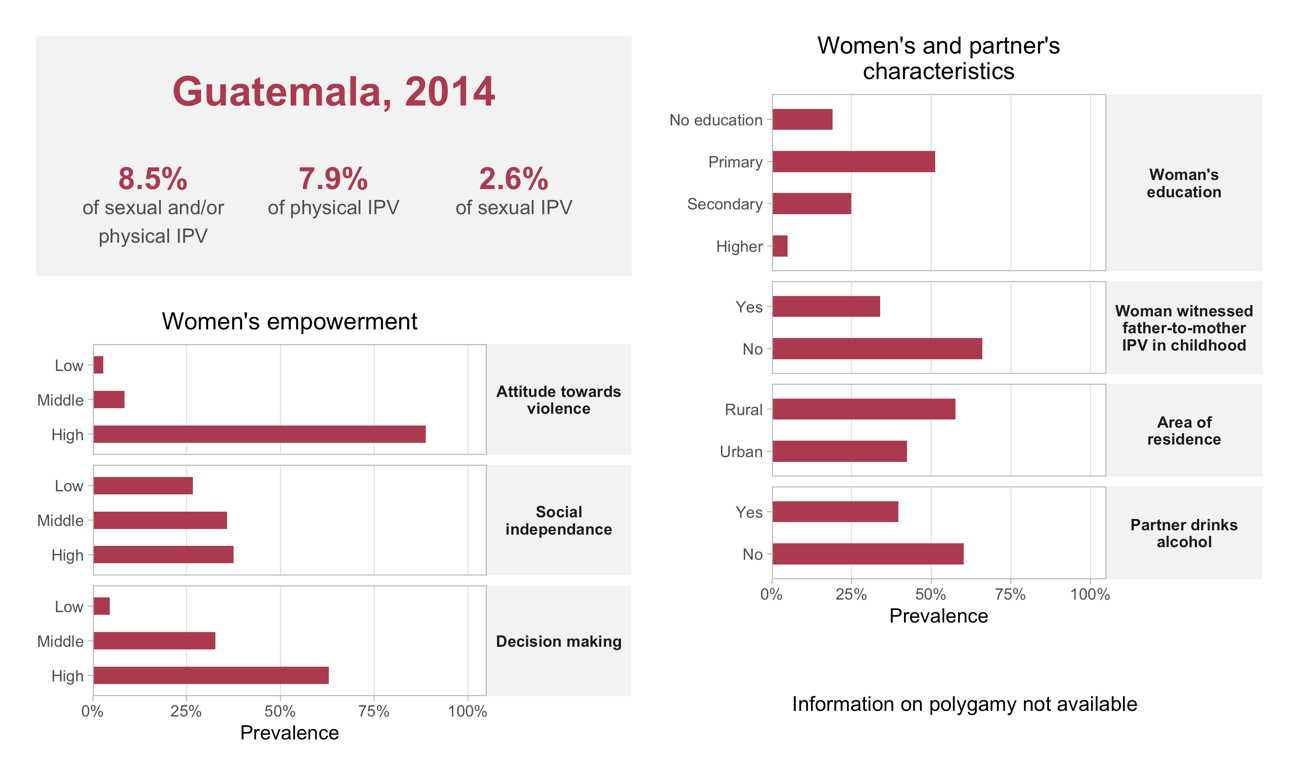
Figure S37 – Guatemala, 2014: country profile


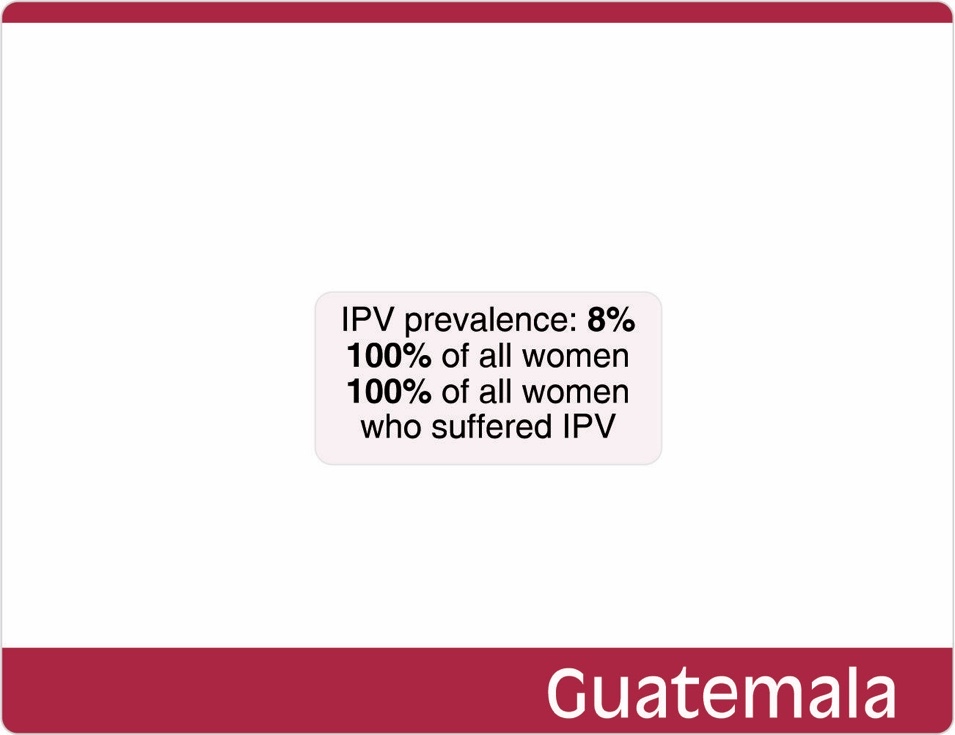


Figure S38 – Guatemala, 2014: decision tree


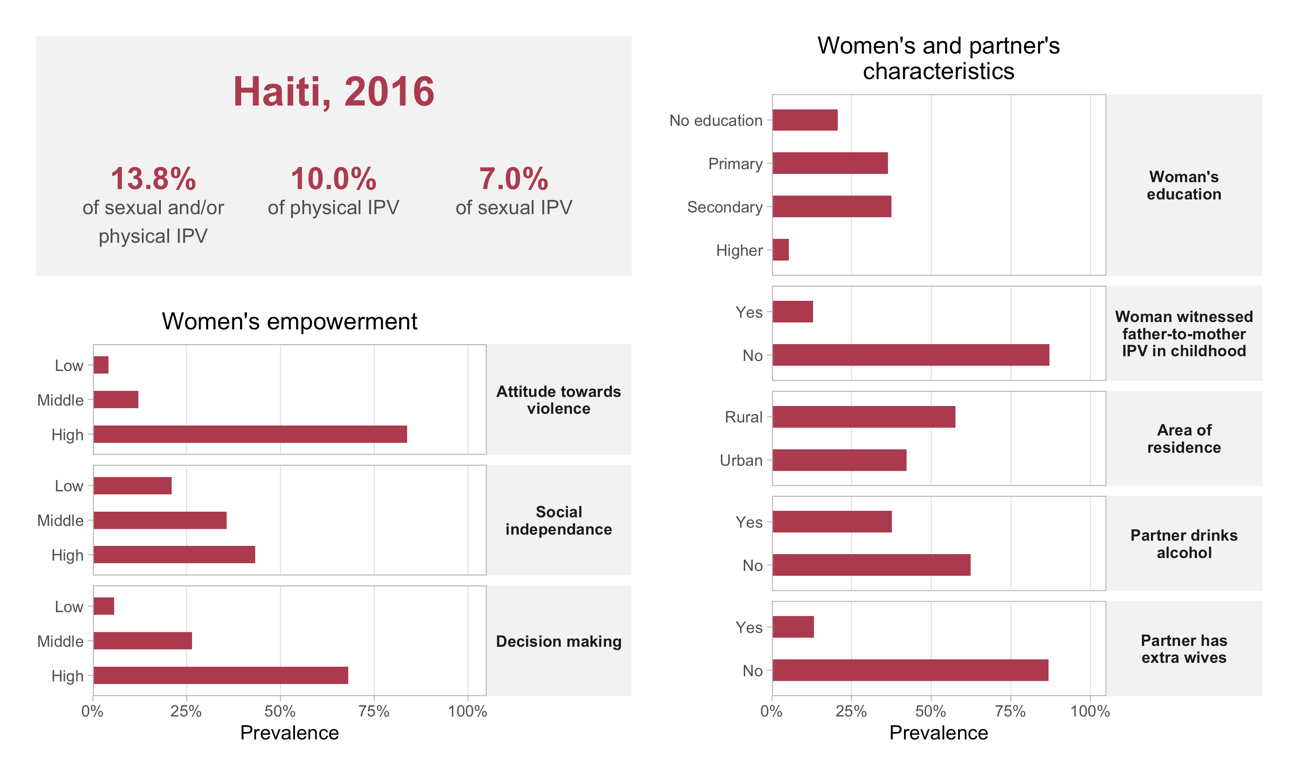
Figure S39 – Haiti, 2016: country profile


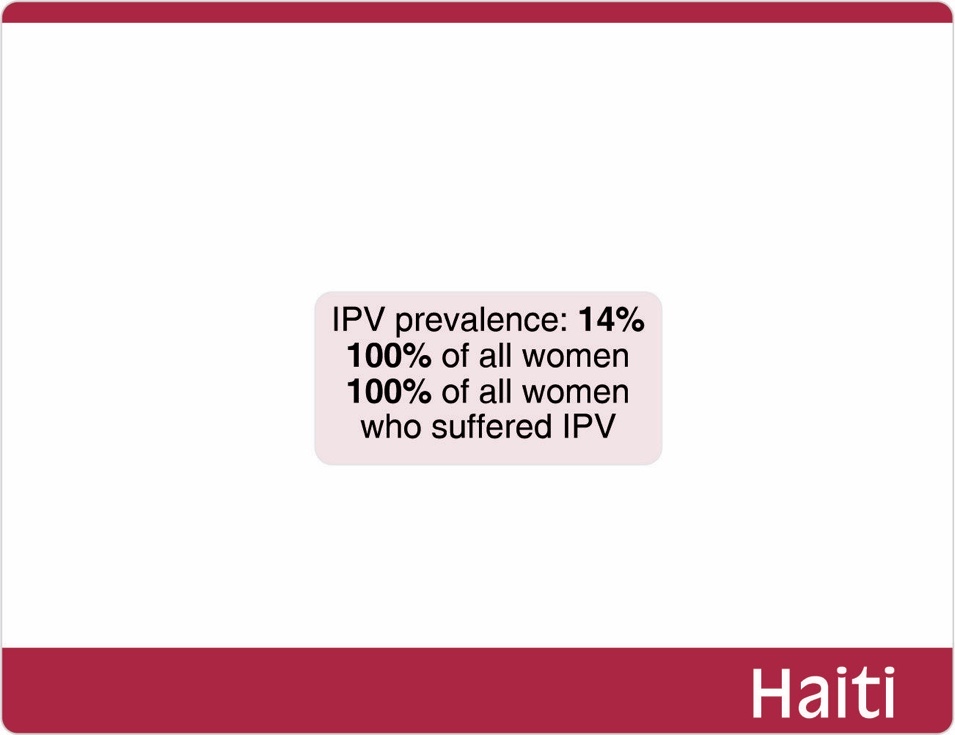


Figure S40 – Haiti, 2016: decision tree


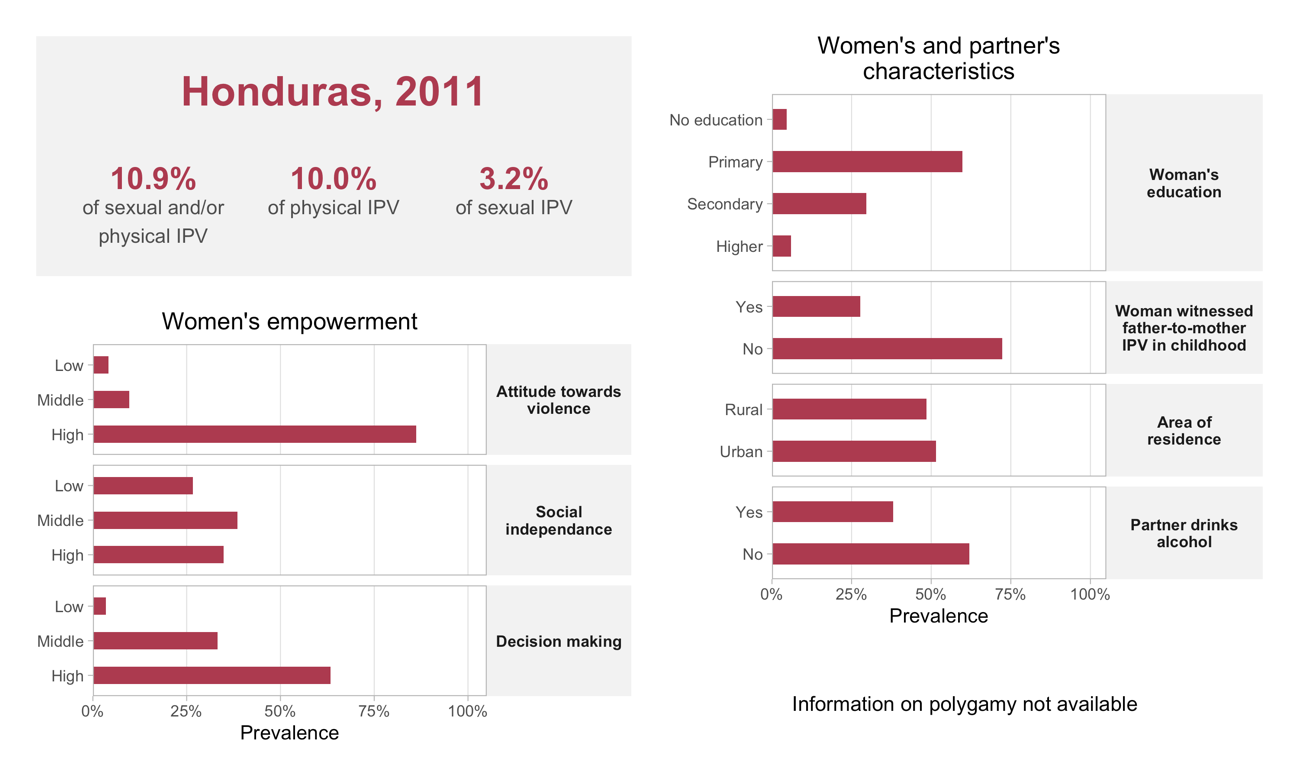
Figure S41 – Honduras, 2011: country profile


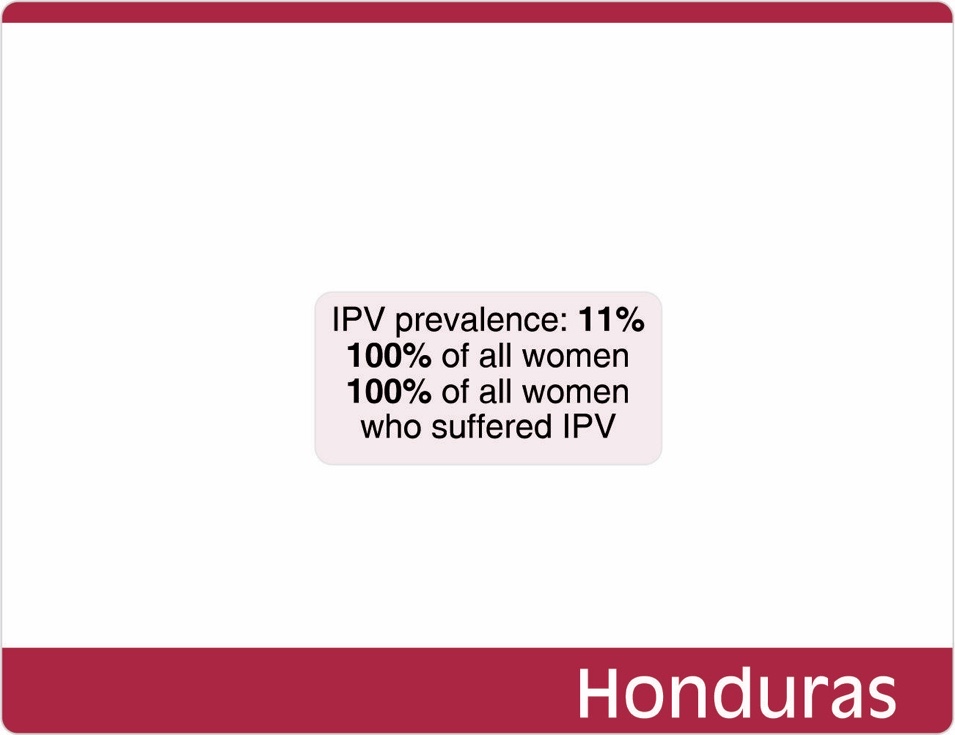


Figure S42 – Honduras, 2011: decision tree


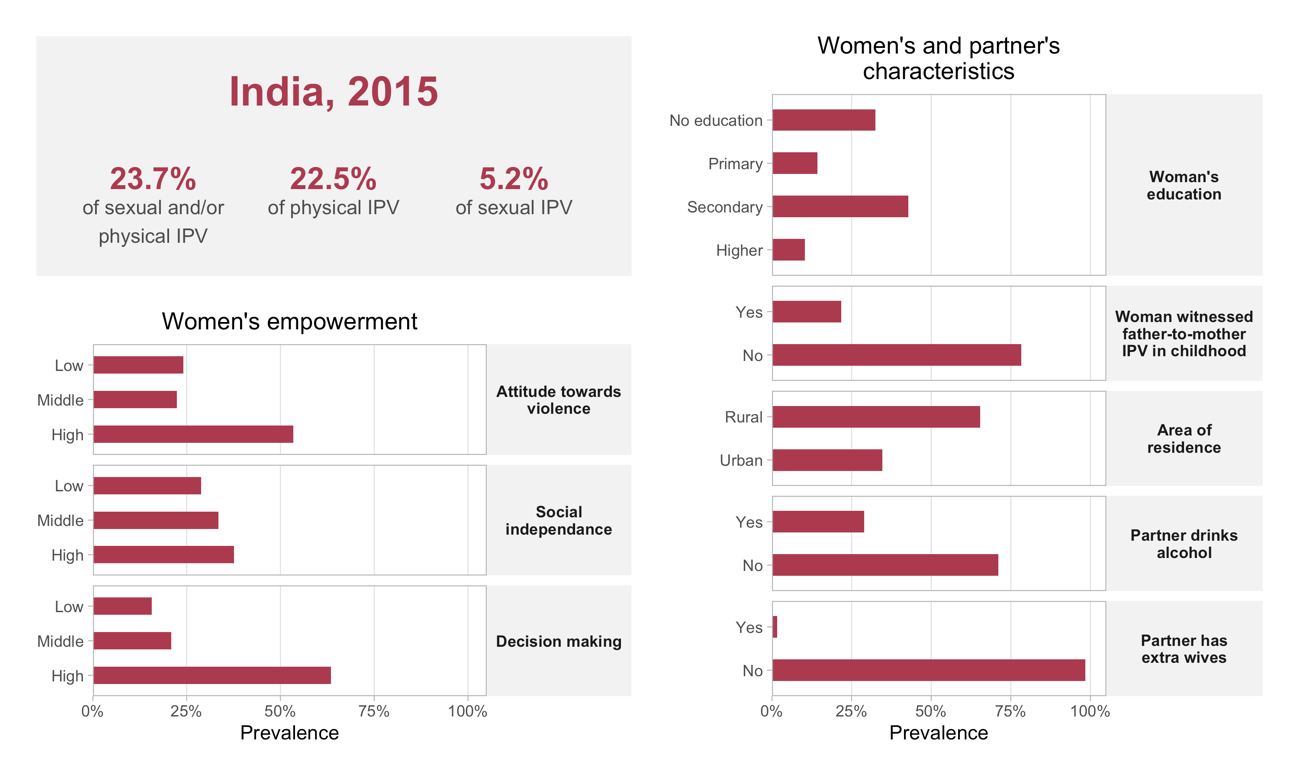
Figure S43 – India, 2015: country profile


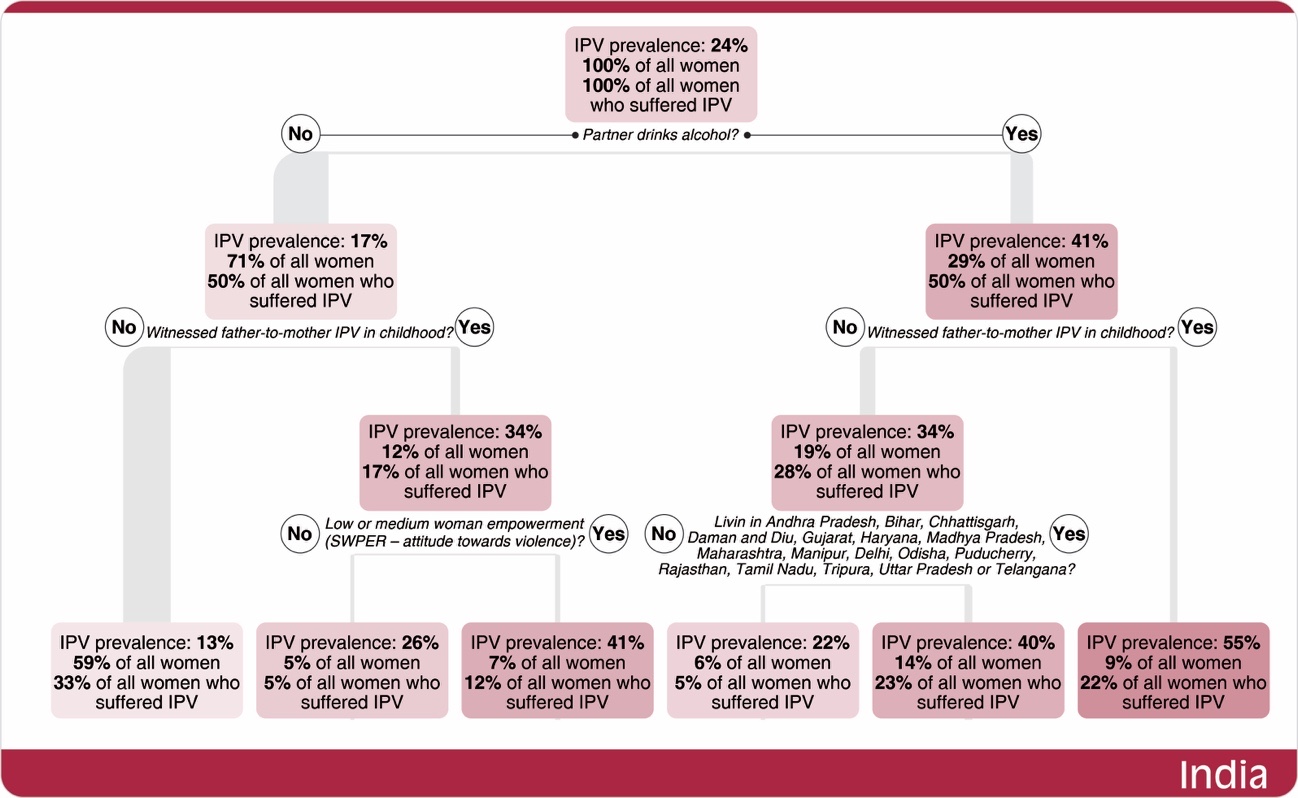


Figure S44 – India, 2015: decision tree


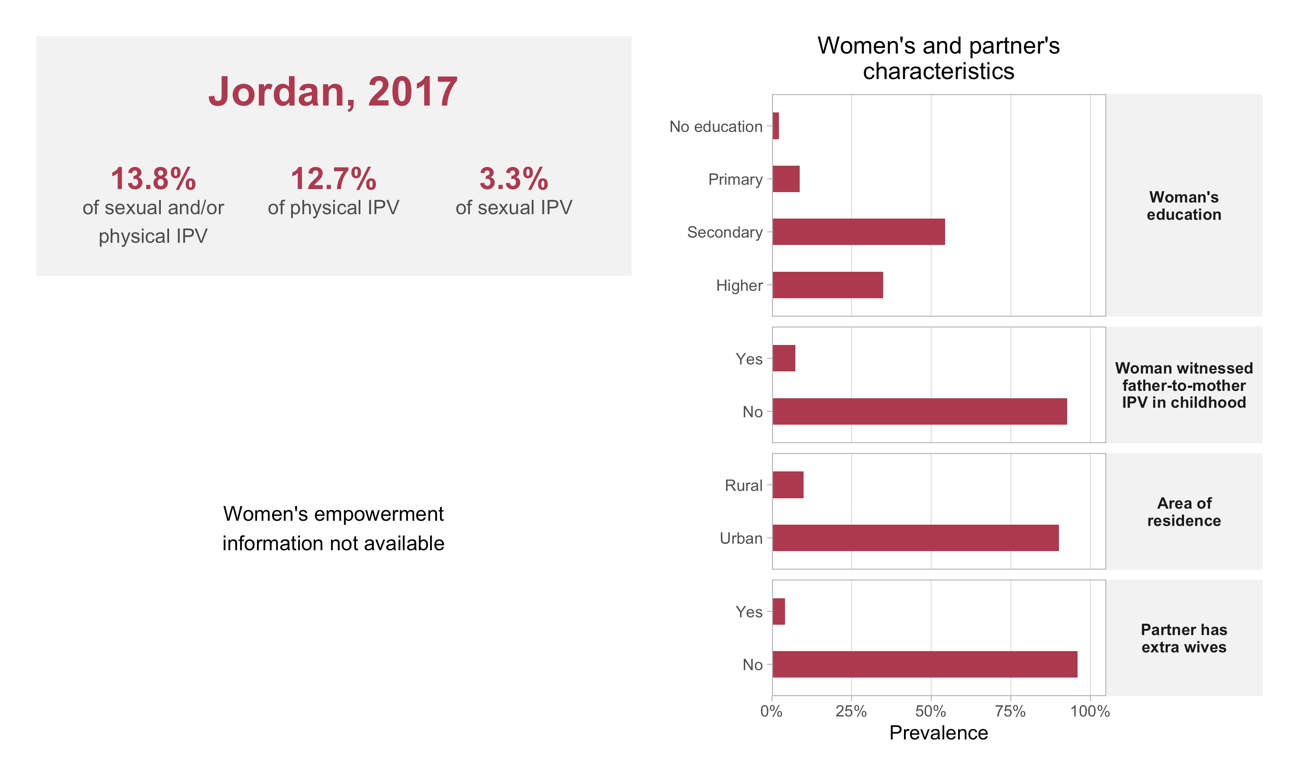
Figure S45 – Jordan, 2017: country profile


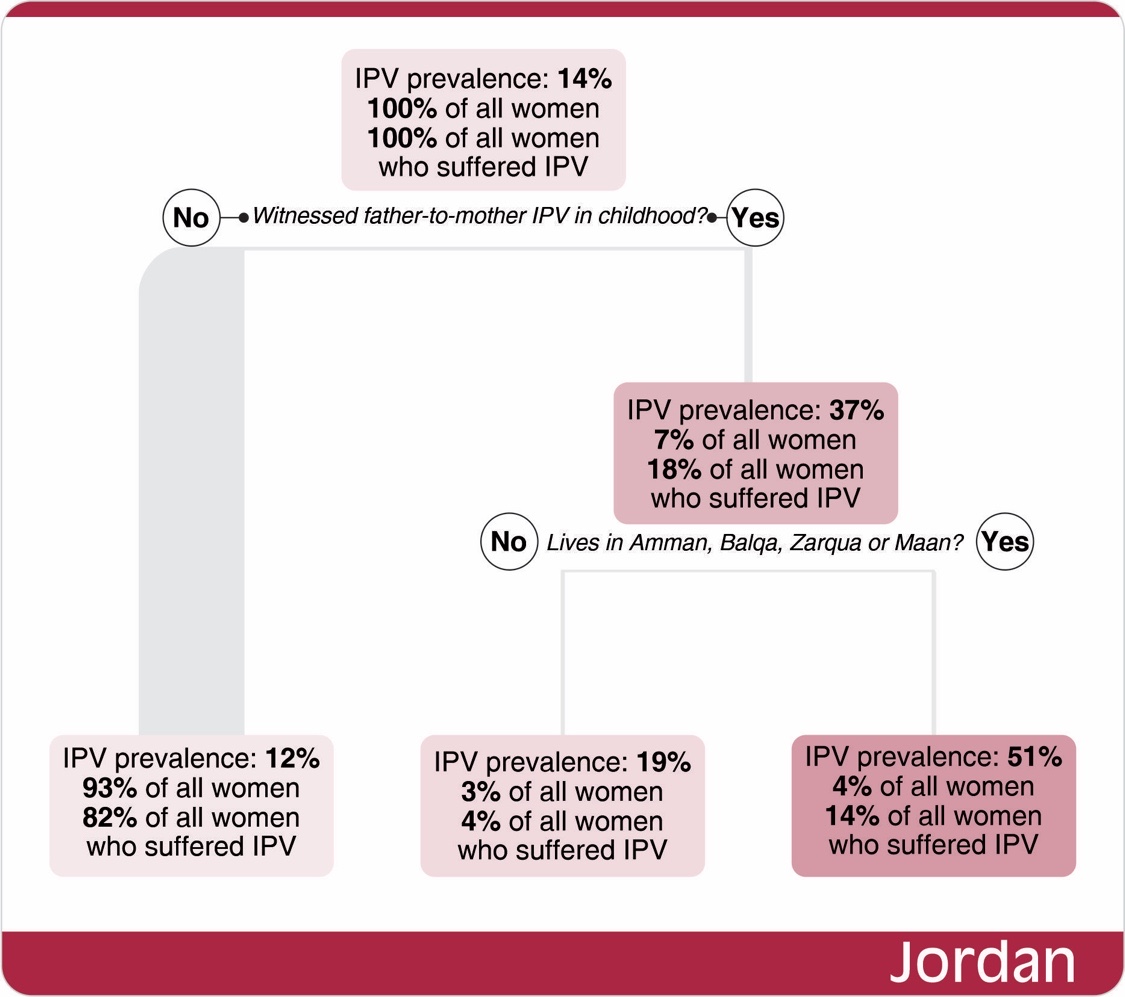


Figure S46 – Jordan, 2017: decision tree


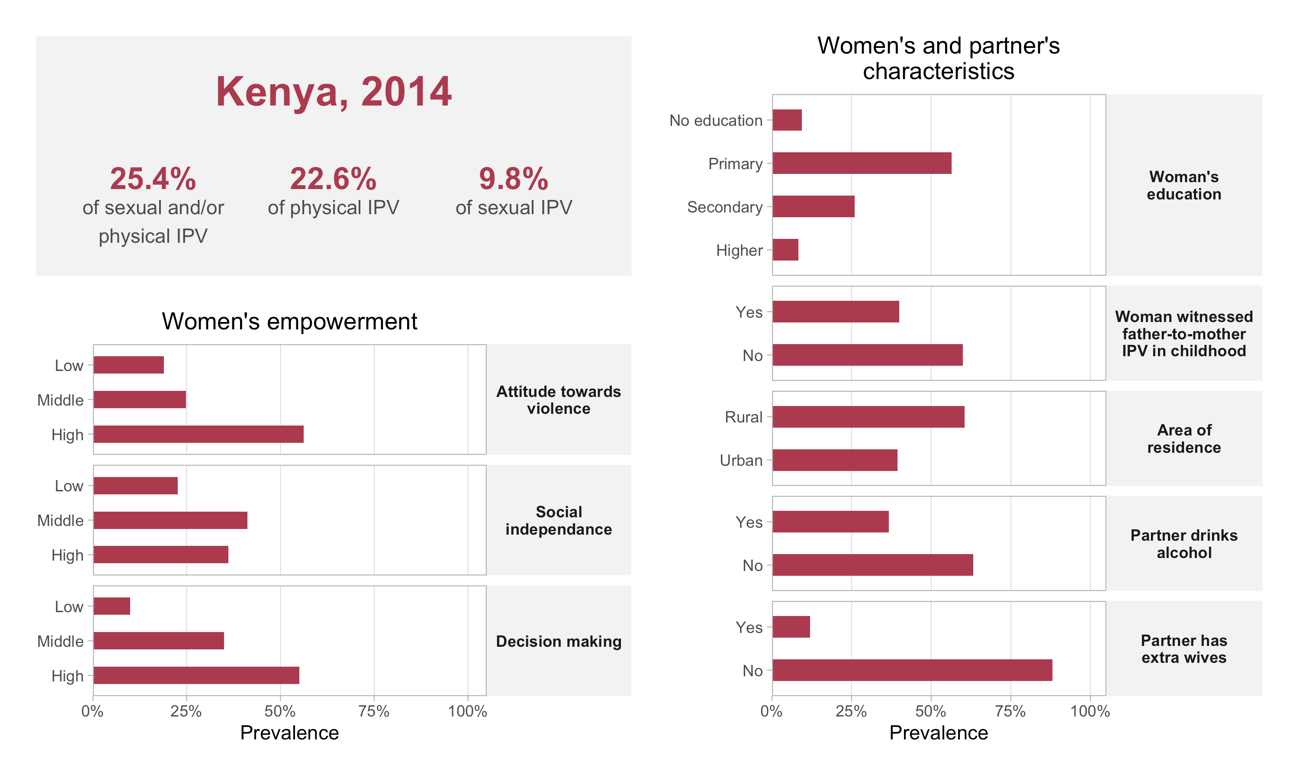
Figure S47 – Kenya, 2014: country profile


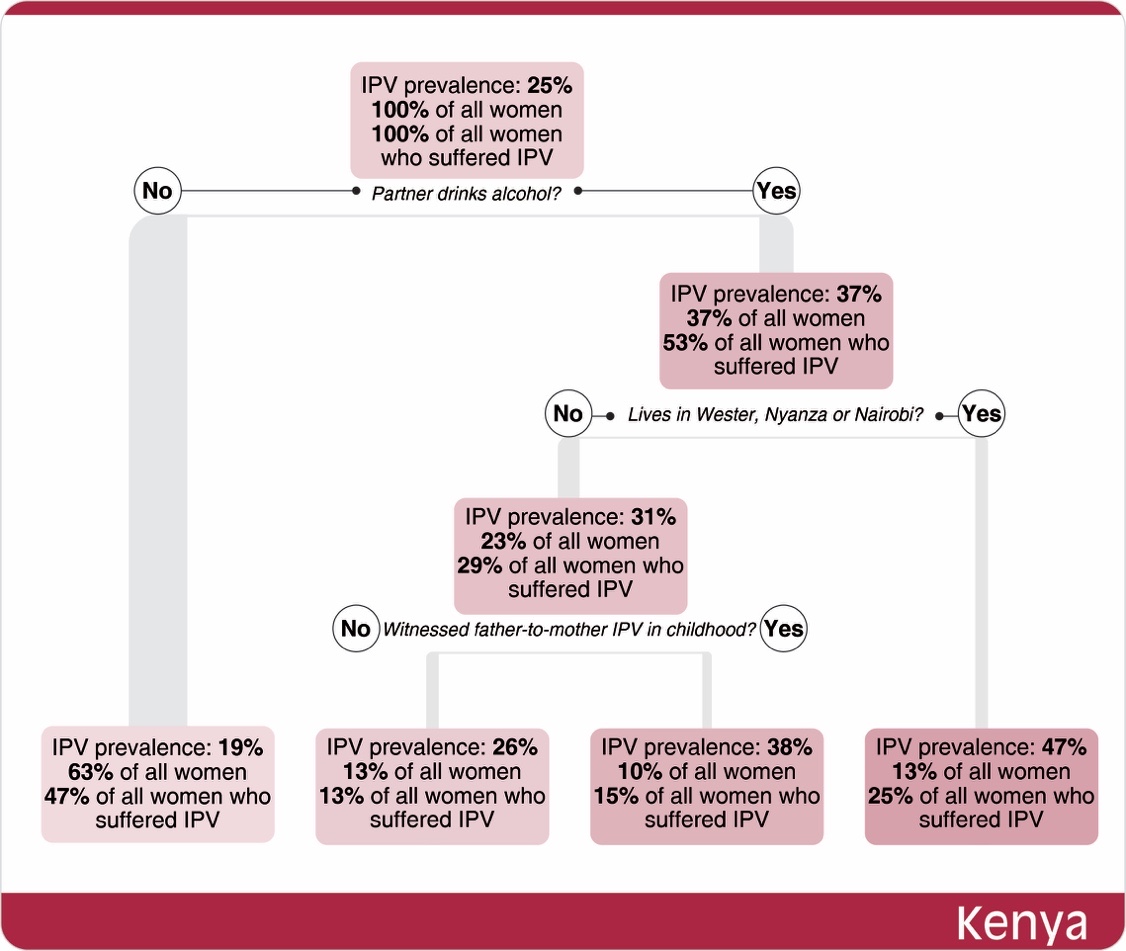


Figure S48 – Kenya, 2014: decision tree


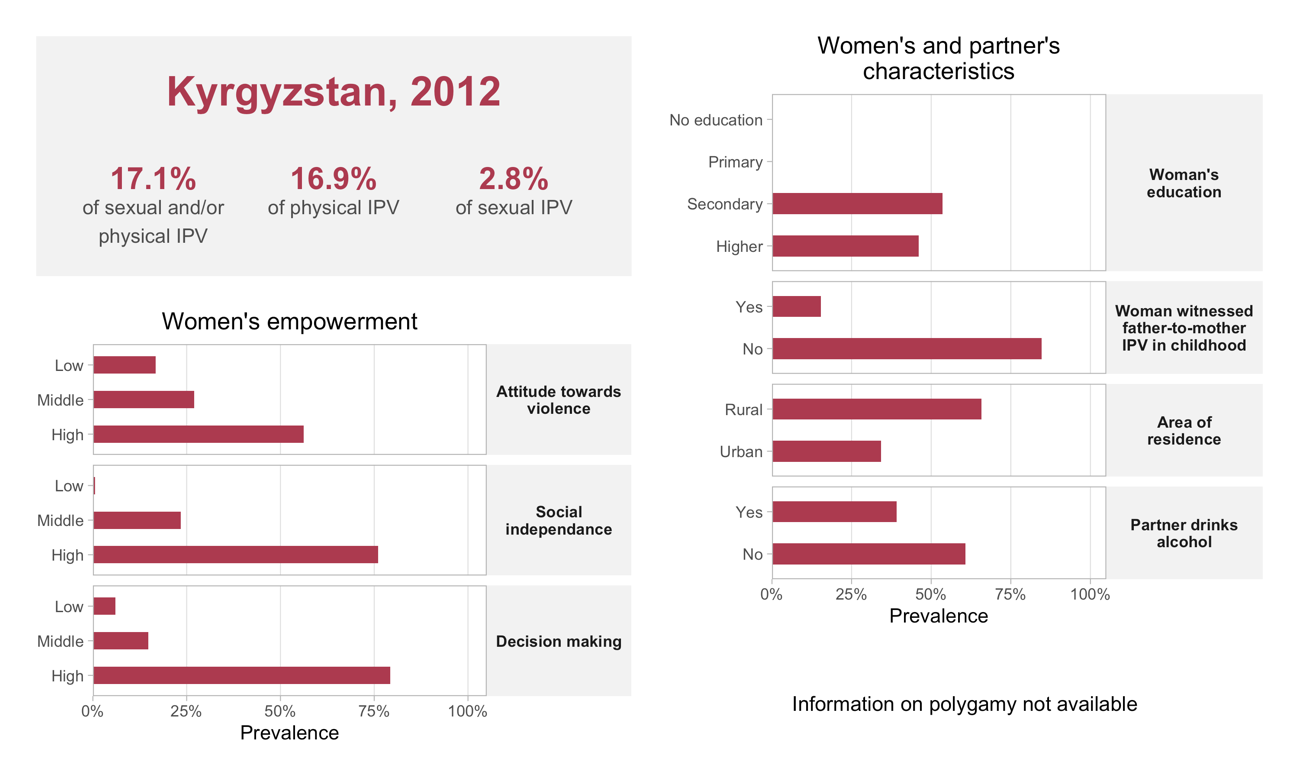
Figure S49 – Kyrgyzstan, 2012: country profile


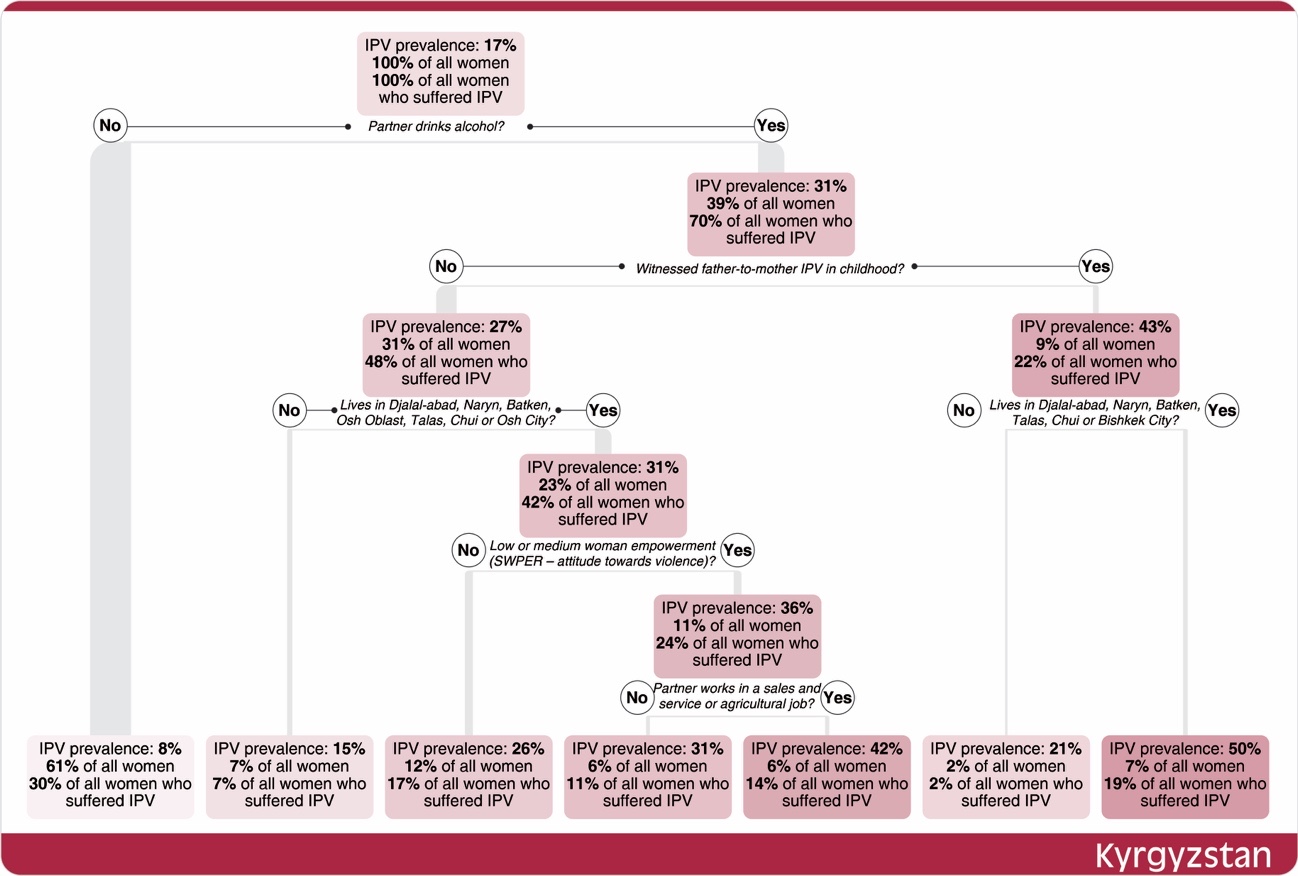


Figure S50 – Kyrgyzstan, 2012: decision tree


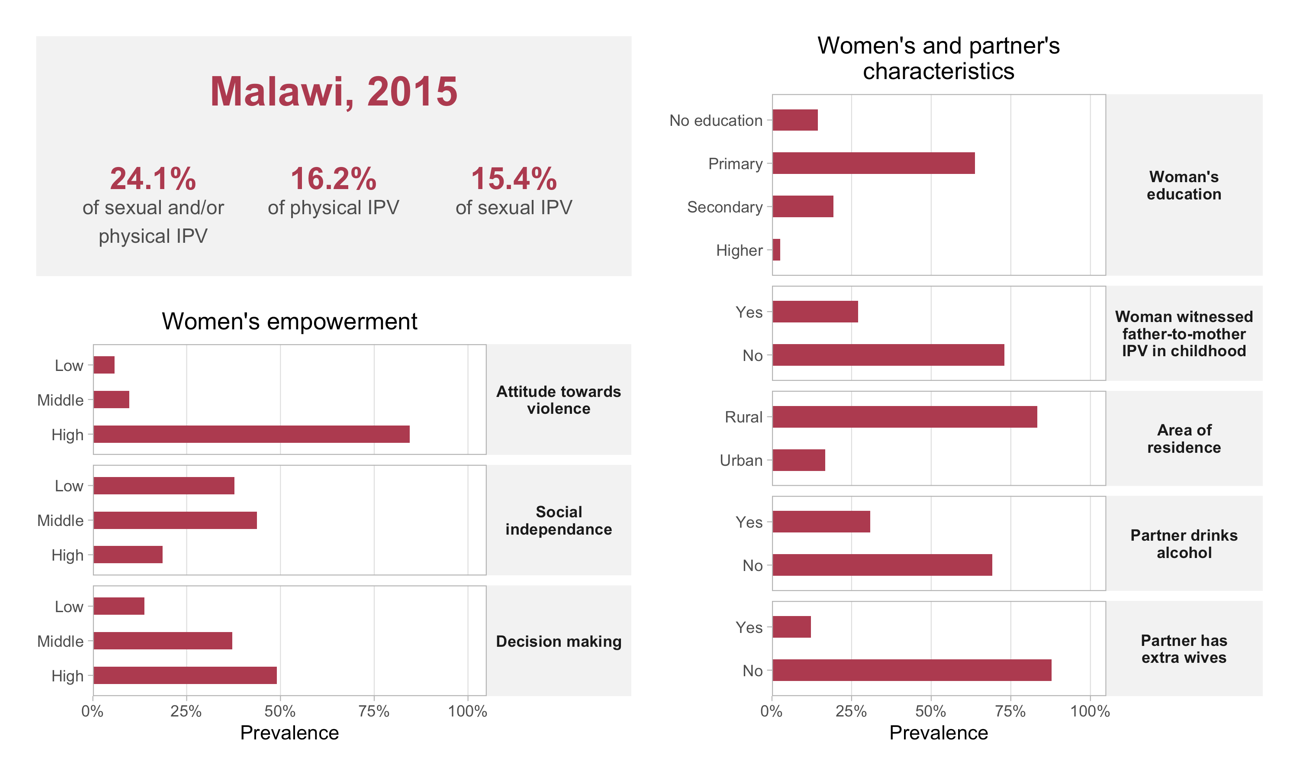
Figure S51 – Malawi, 2015: country profile


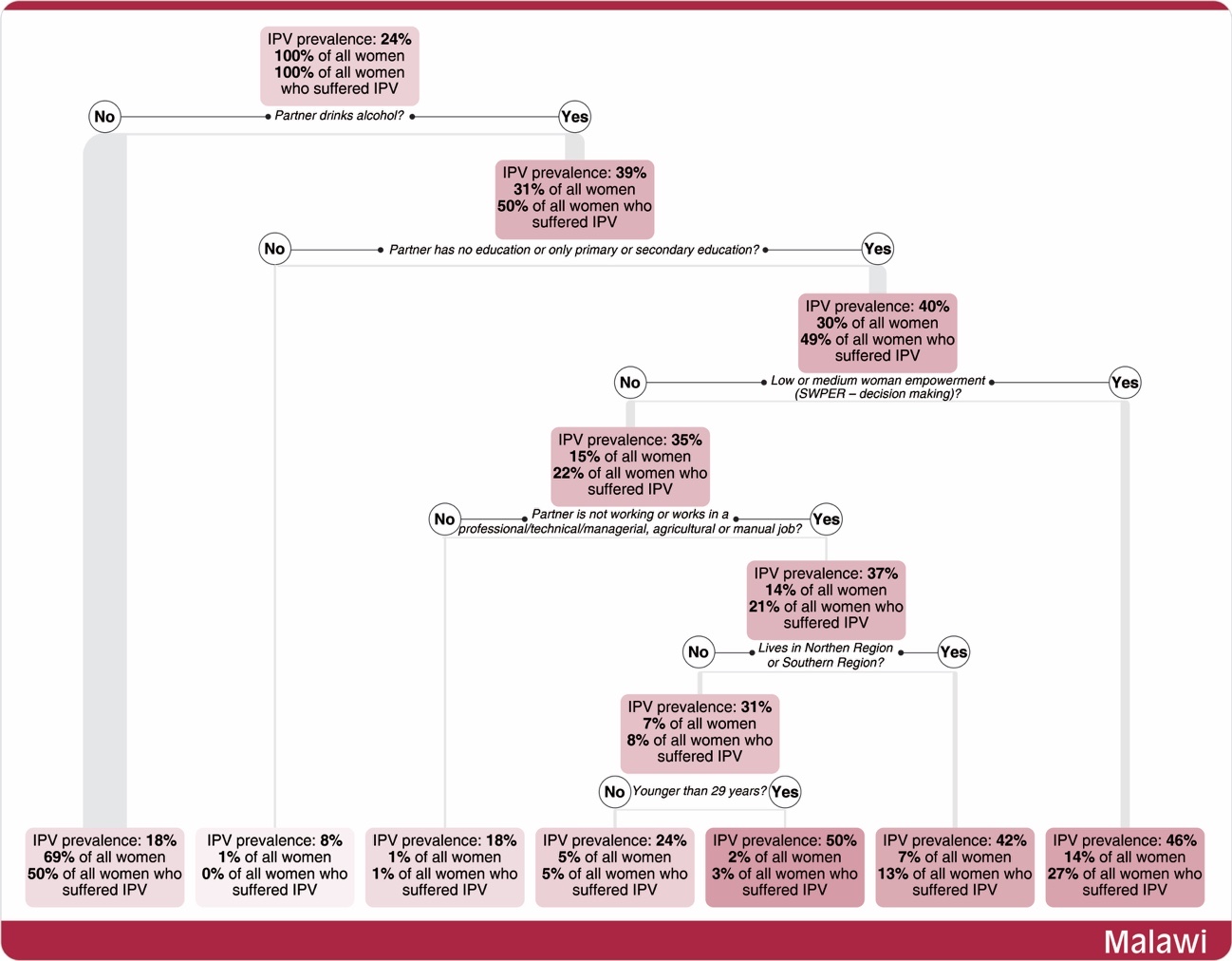


Figure S52 – Malawi, 2015: decision tree


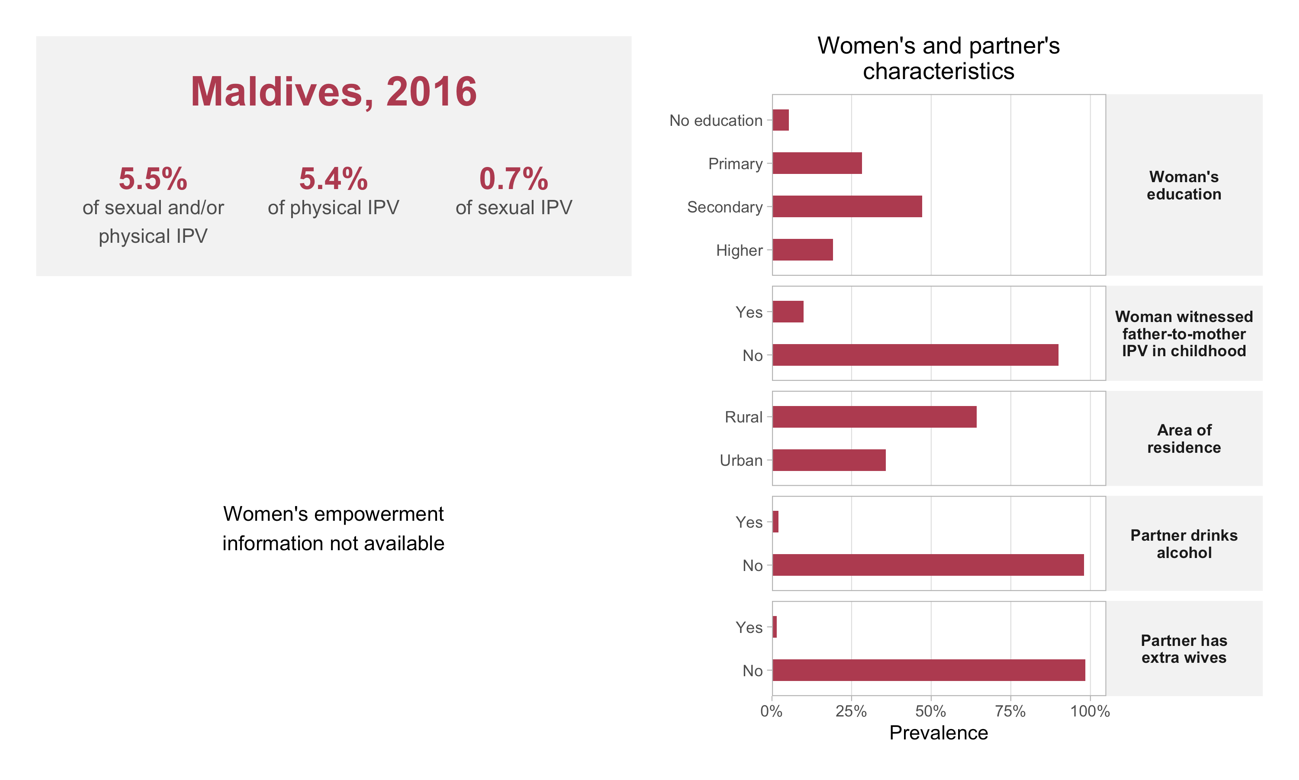
Figure S53 – Maldives, 2016: country profile


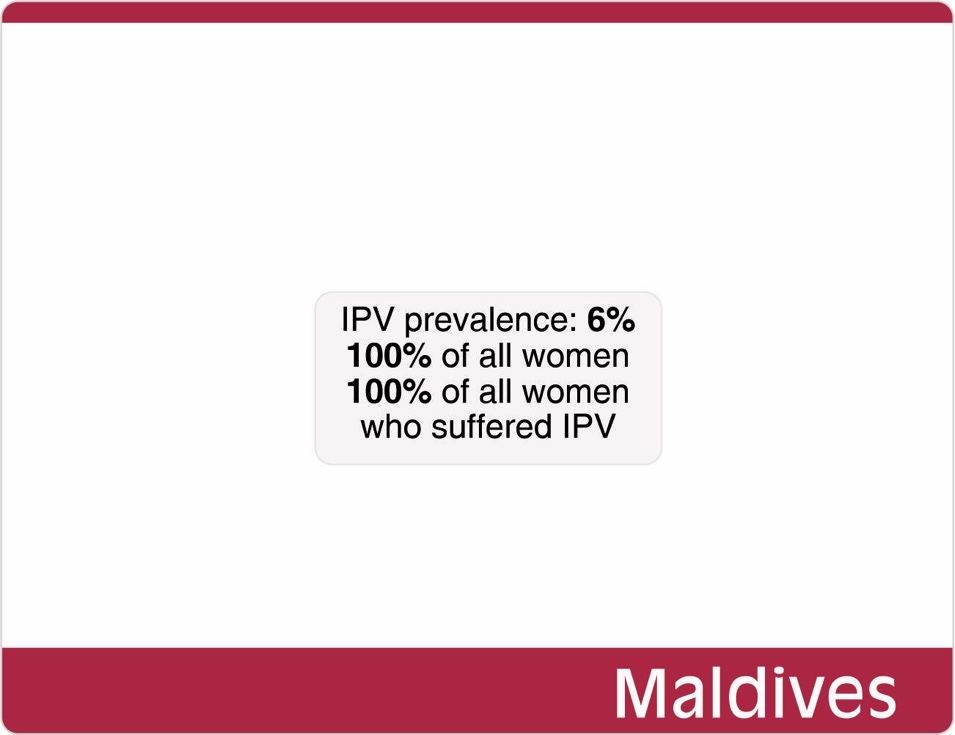


Figure S54 – Maldives, 2016: decision tree


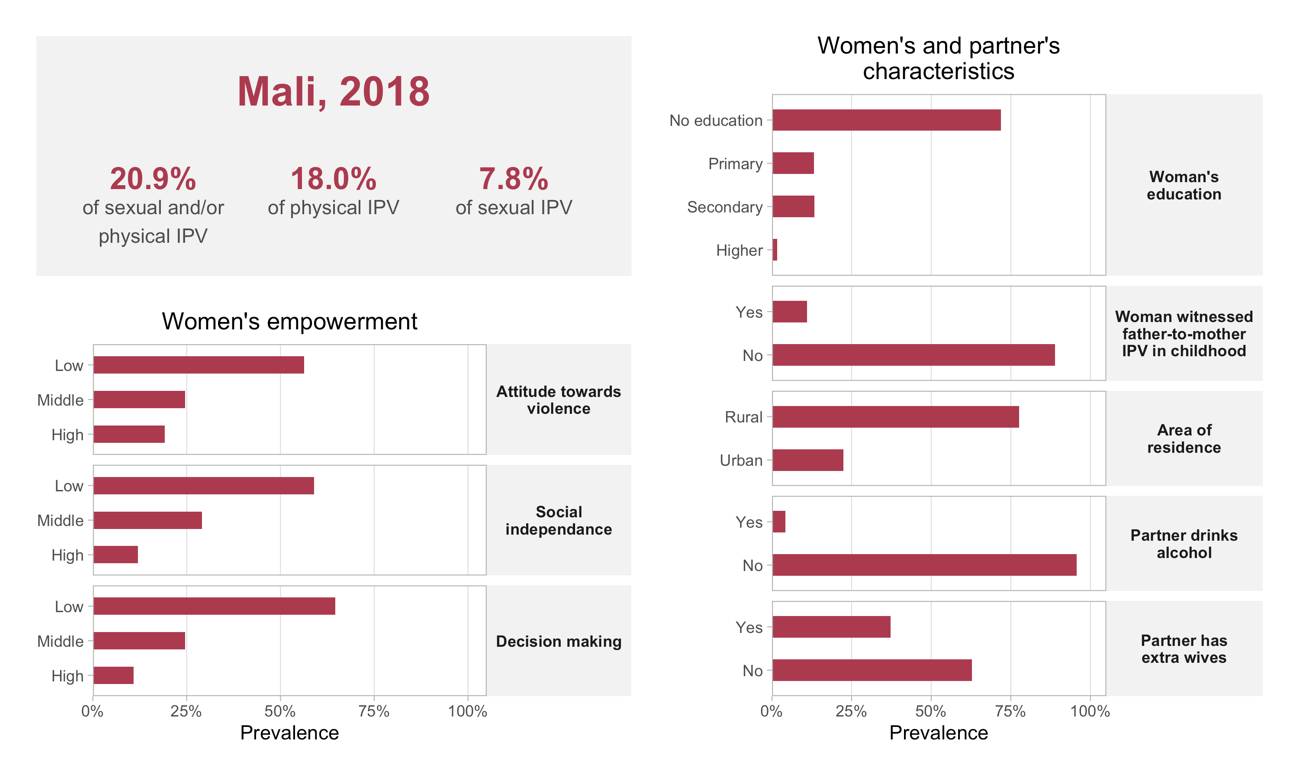
Figure S55 – Mali, 2018: country profile


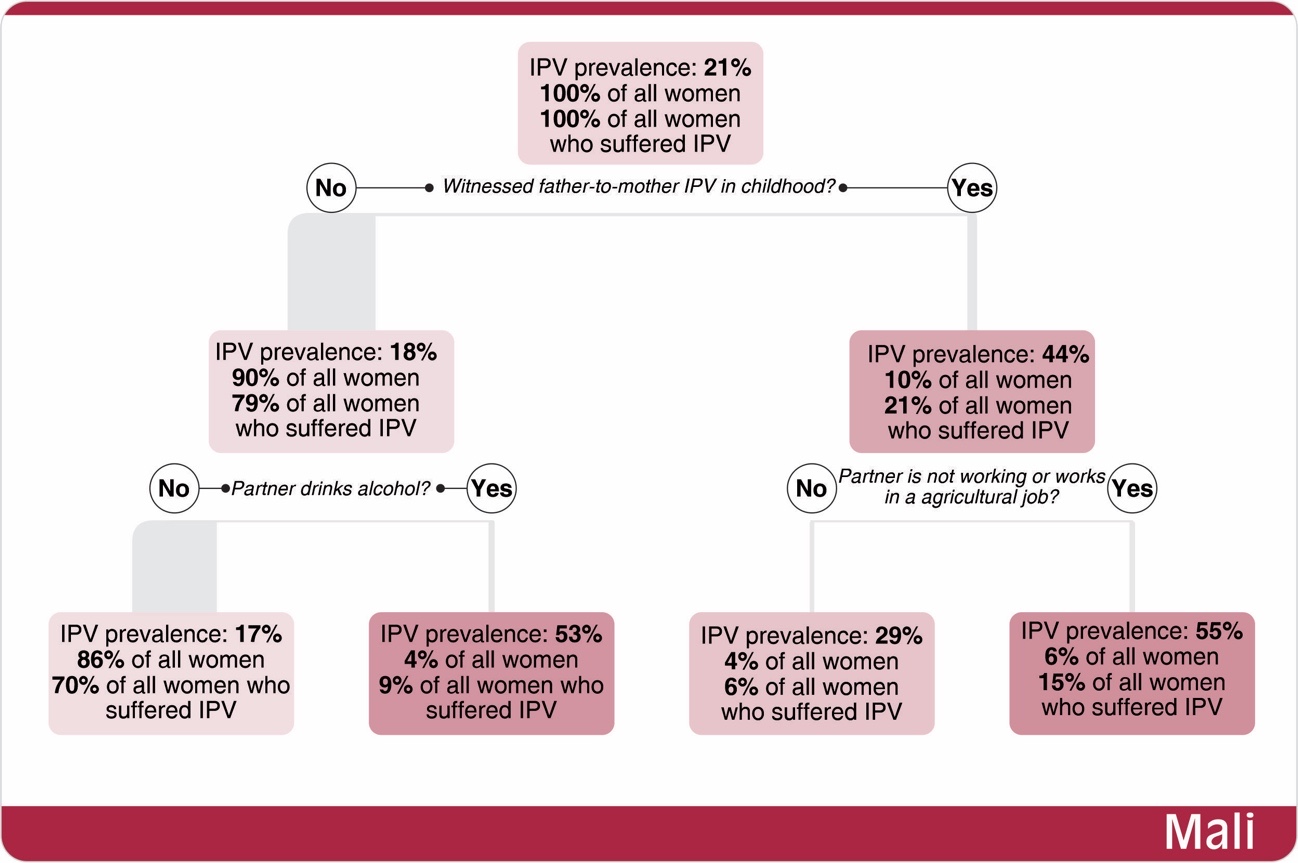


Figure S56 – Mali, 2018: decision tree


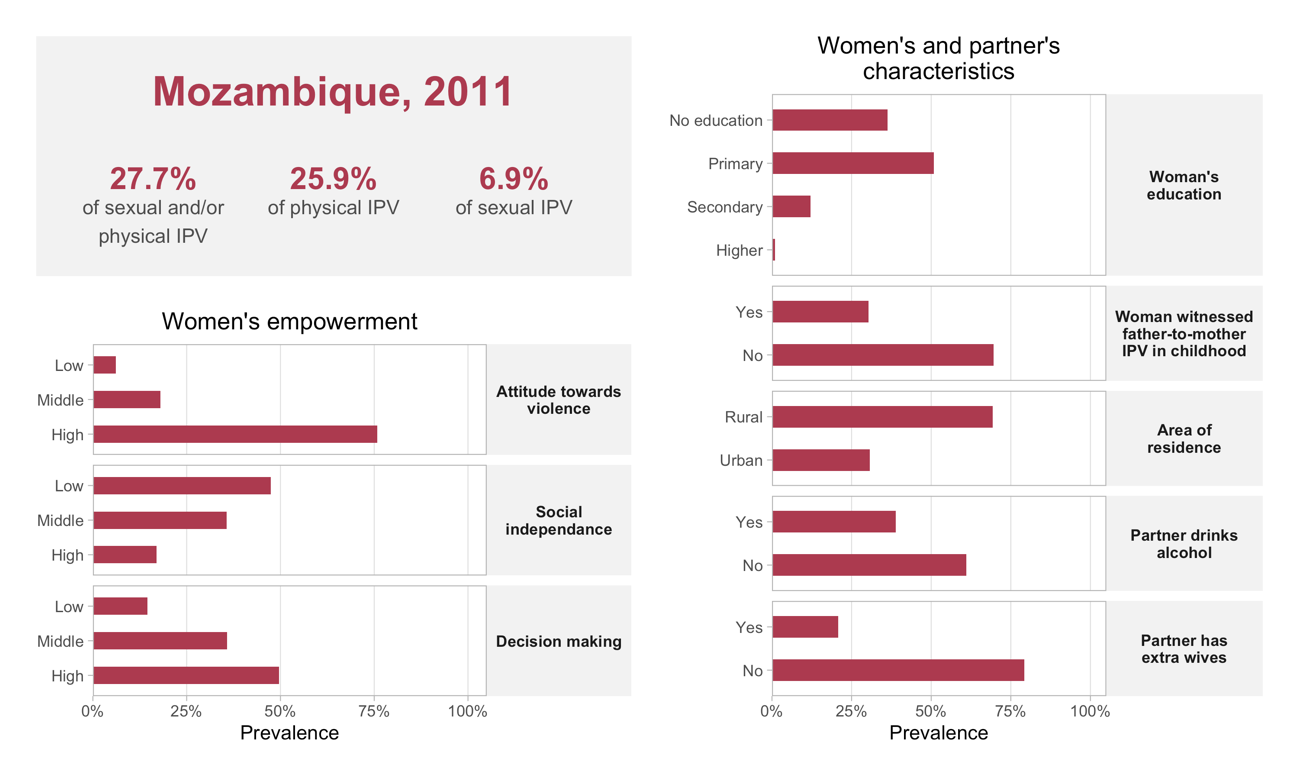
Figure S57 – Mozambique, 2011: country profile


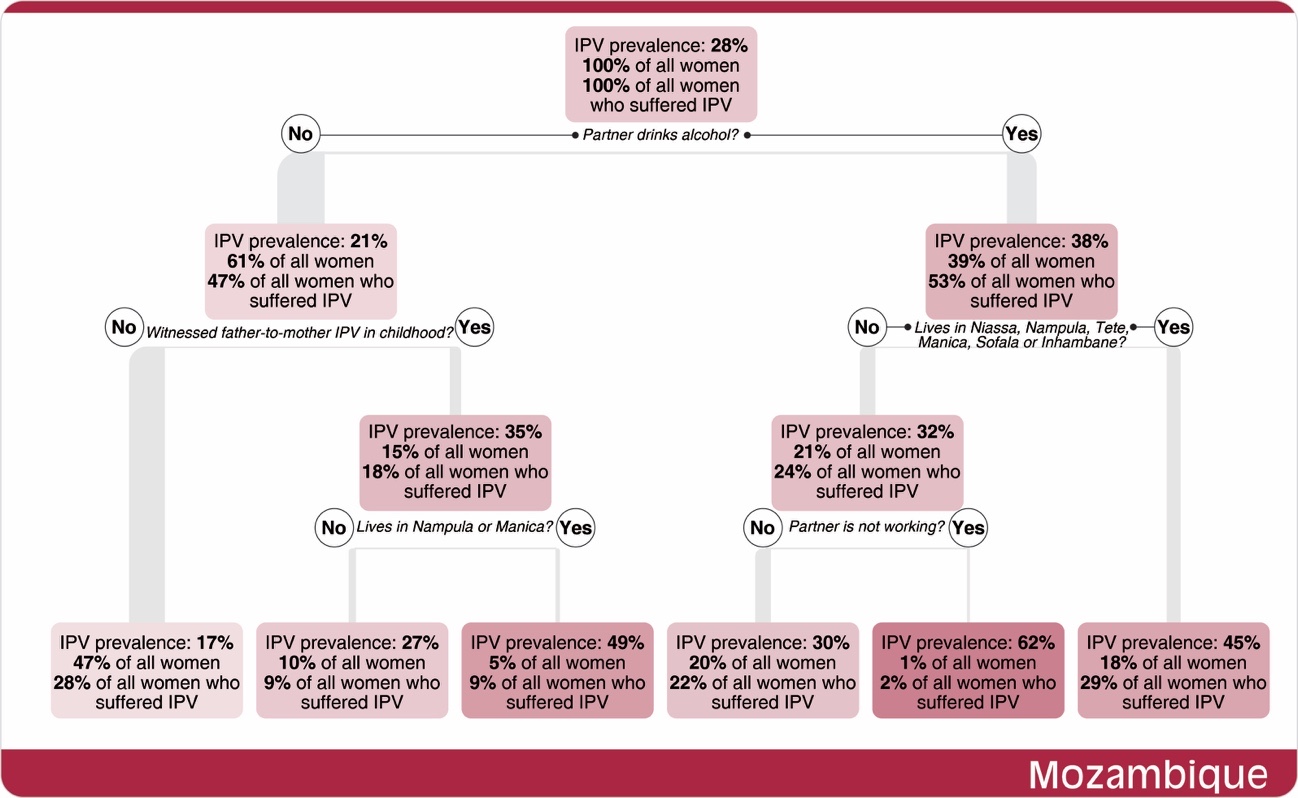


Figure S58 – Mozambique, 2011: decision tree


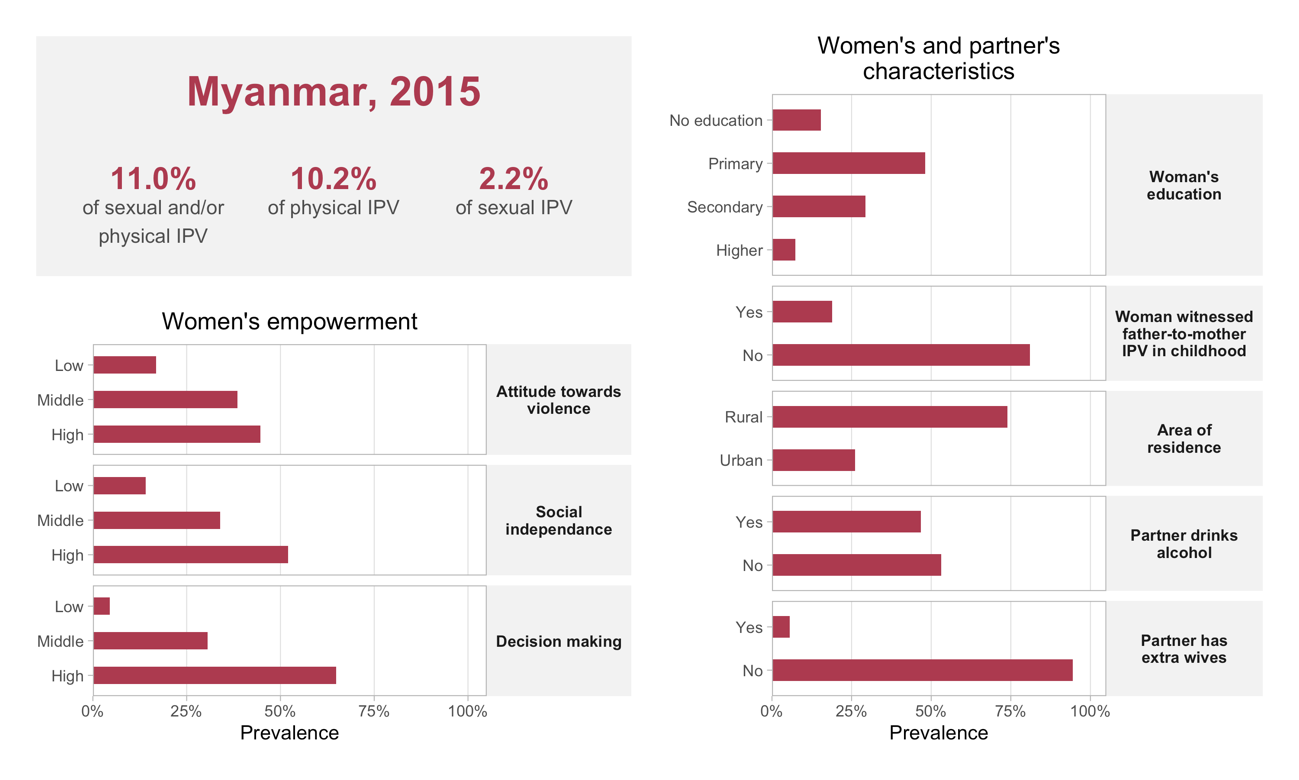
Figure S59 – Myanmar, 2015: country profile


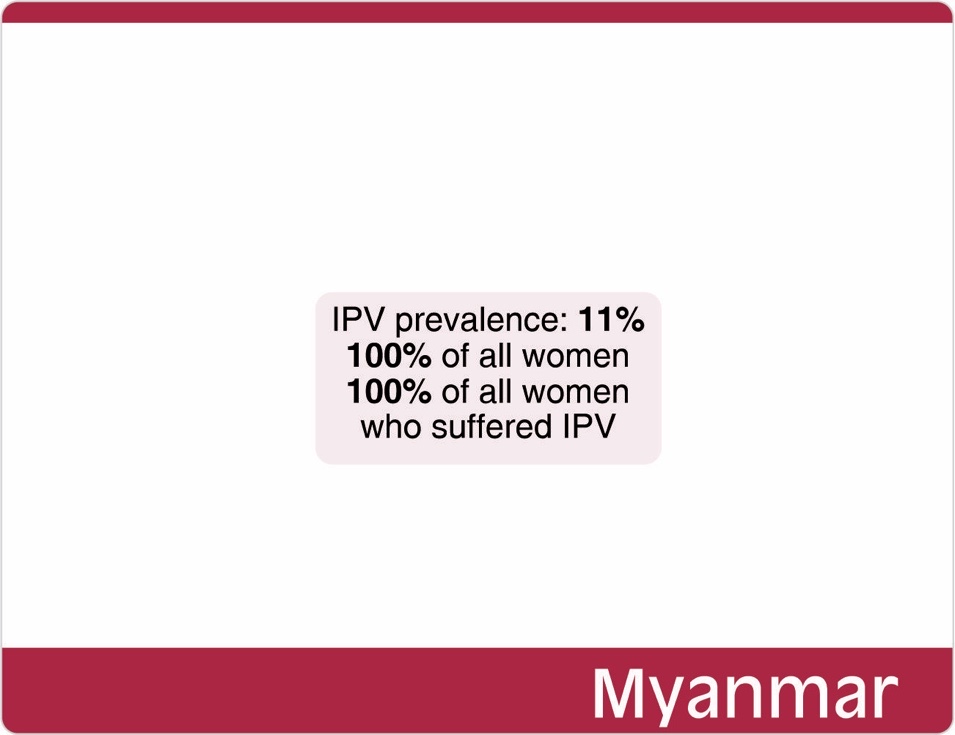


Figure S60 – Myanmar, 2015: decision tree


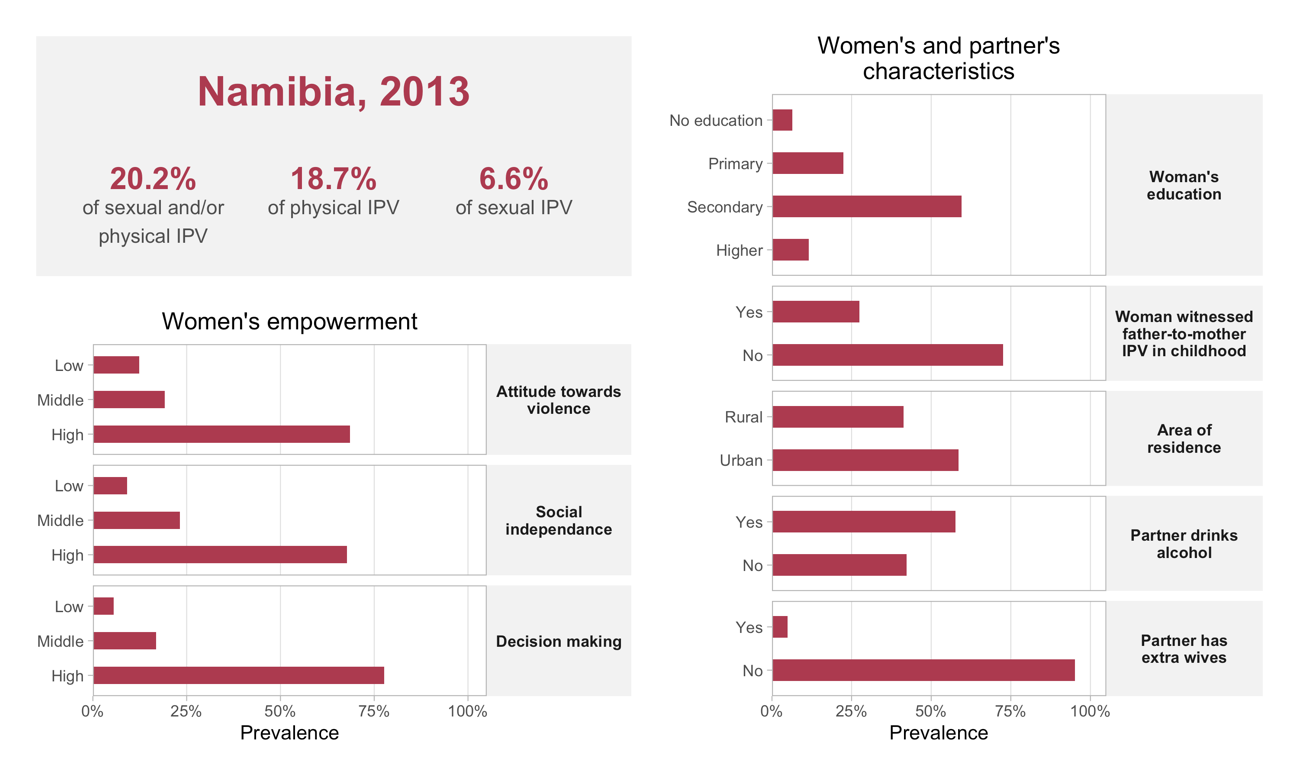
Figure S61 – Namibia, 2013: country profile


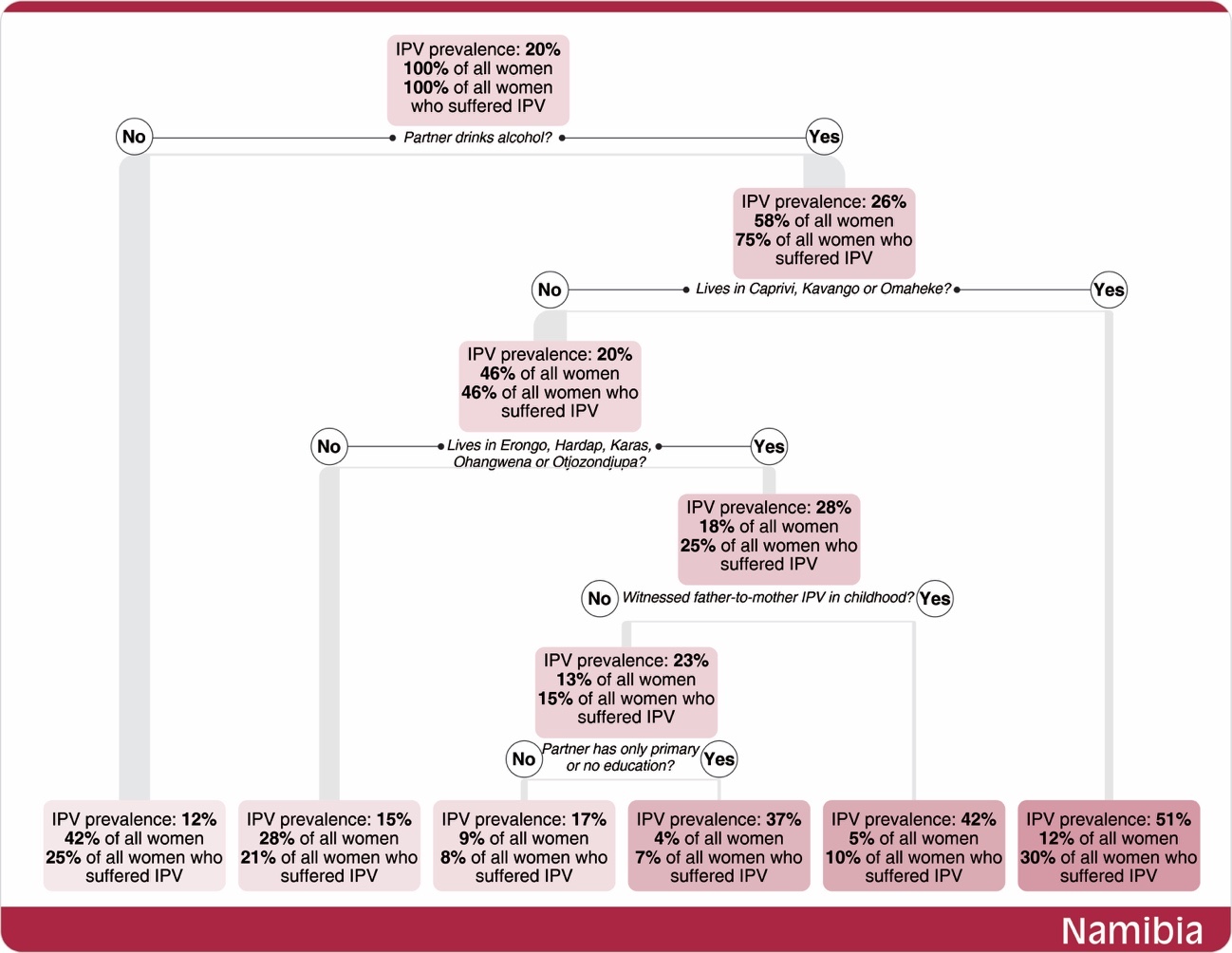


Figure S62 – Namibia, 2013: decision tree


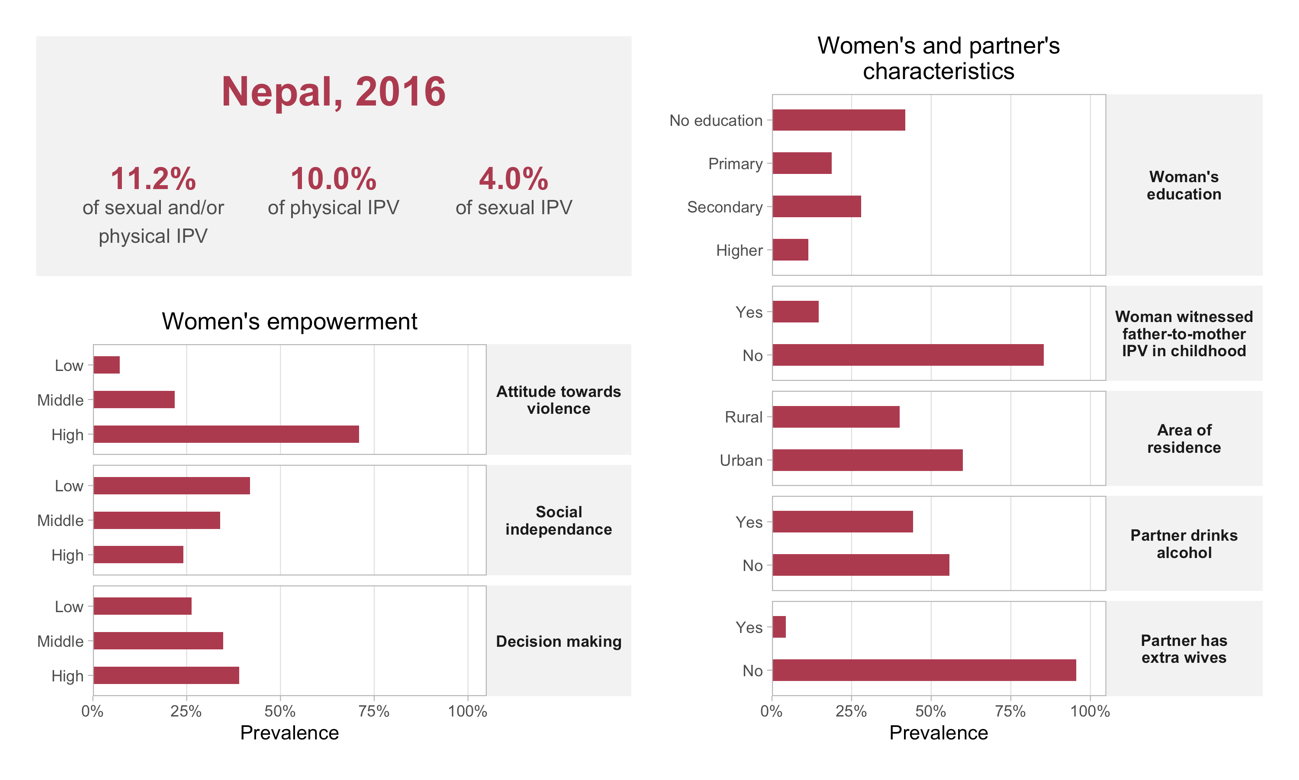
Figure S63 – Nepal, 2016: country profile


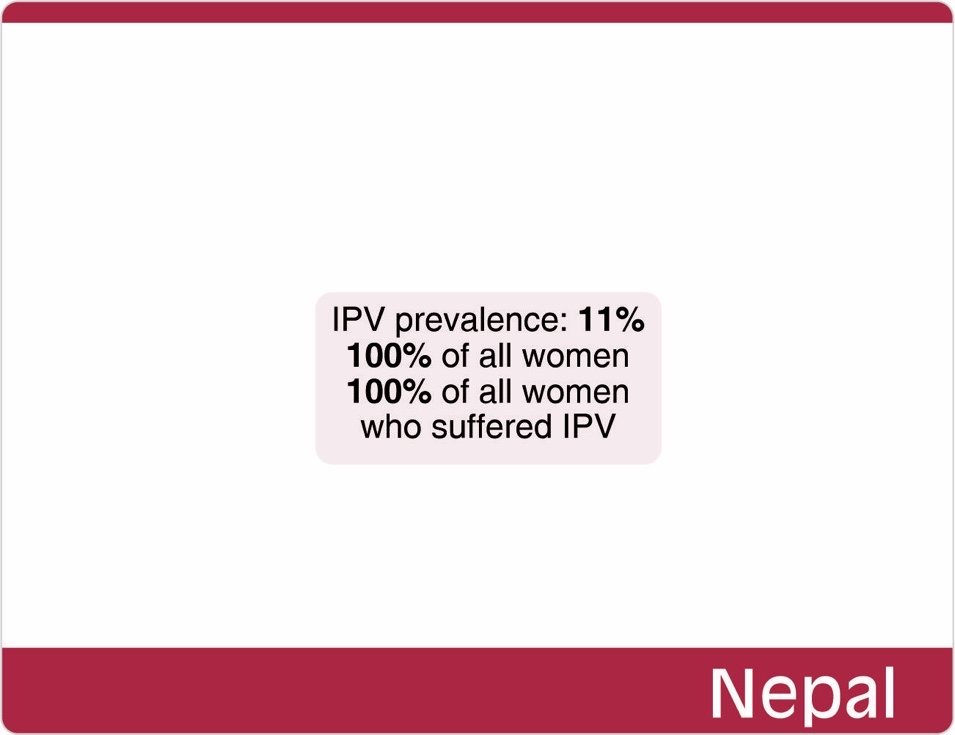


Figure S64 – Nepal, 2016: decision tree


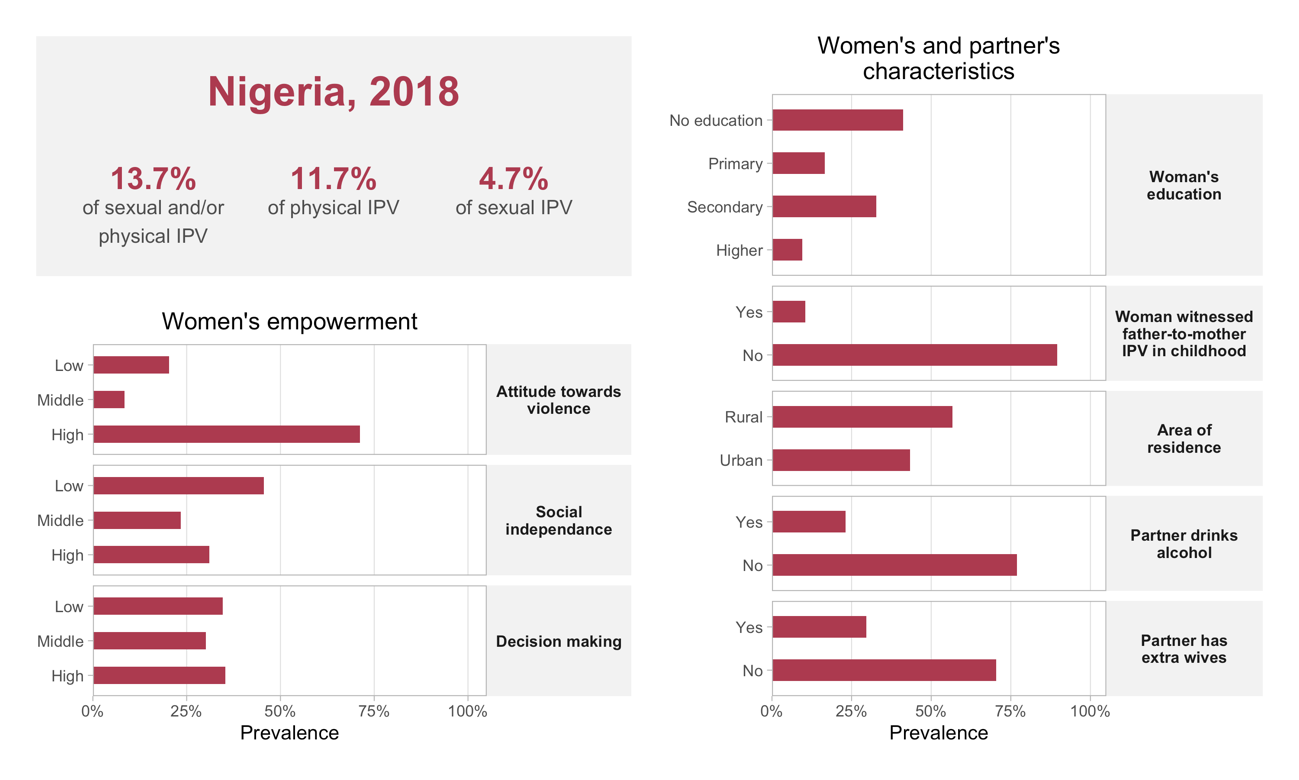
Figure S65 – Nigeria, 2018: country profile


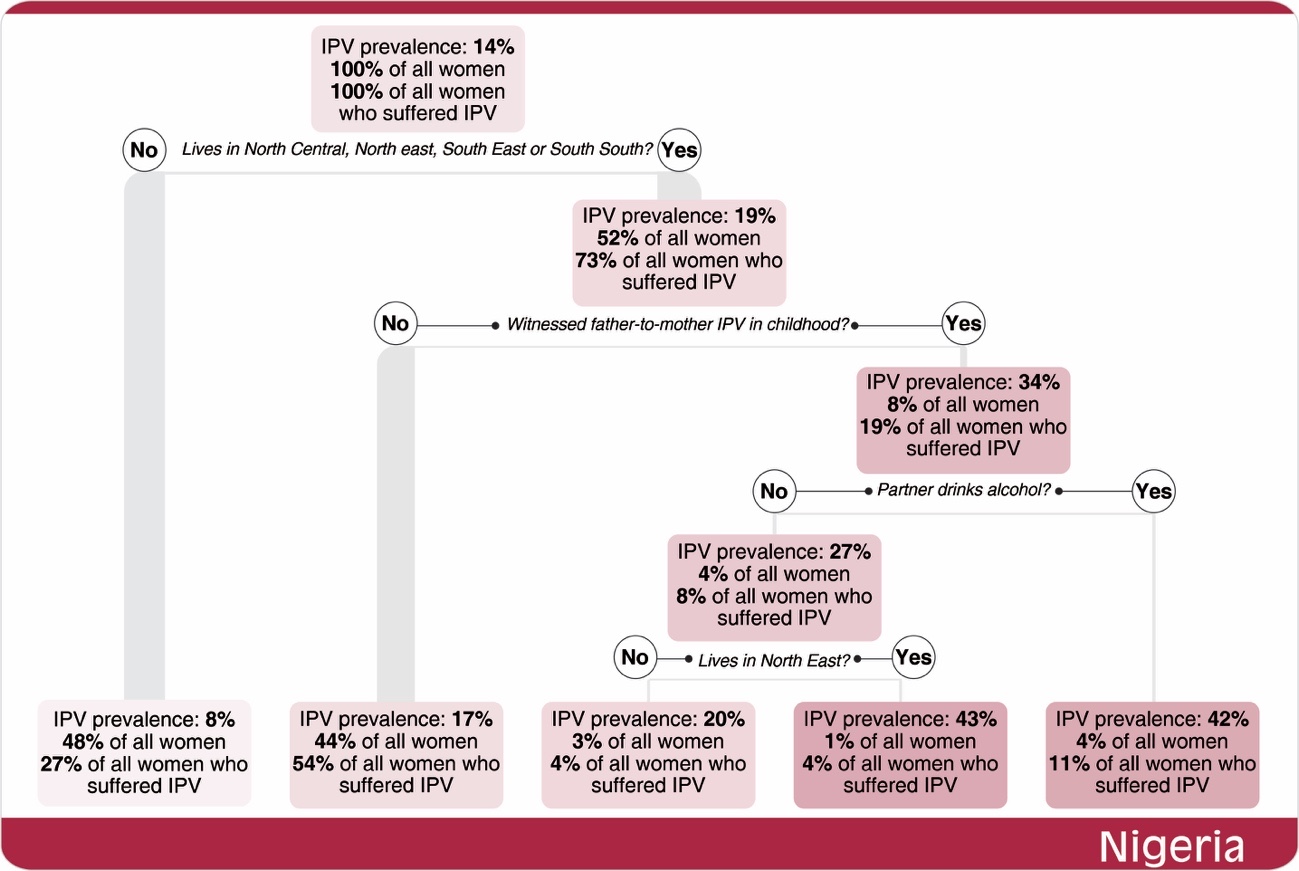


Figure S66 – Nigeria, 2018: decision tree


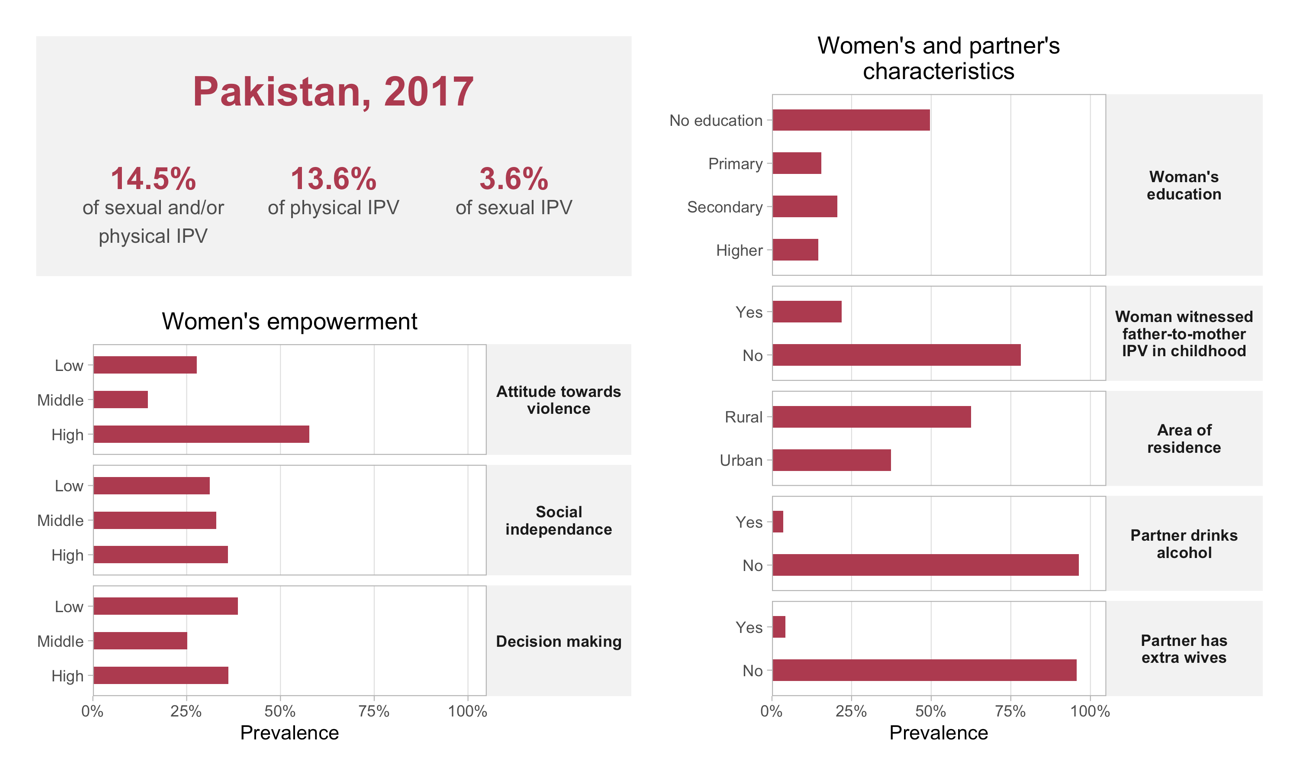
Figure S67 – Pakistan, 2017: country profile


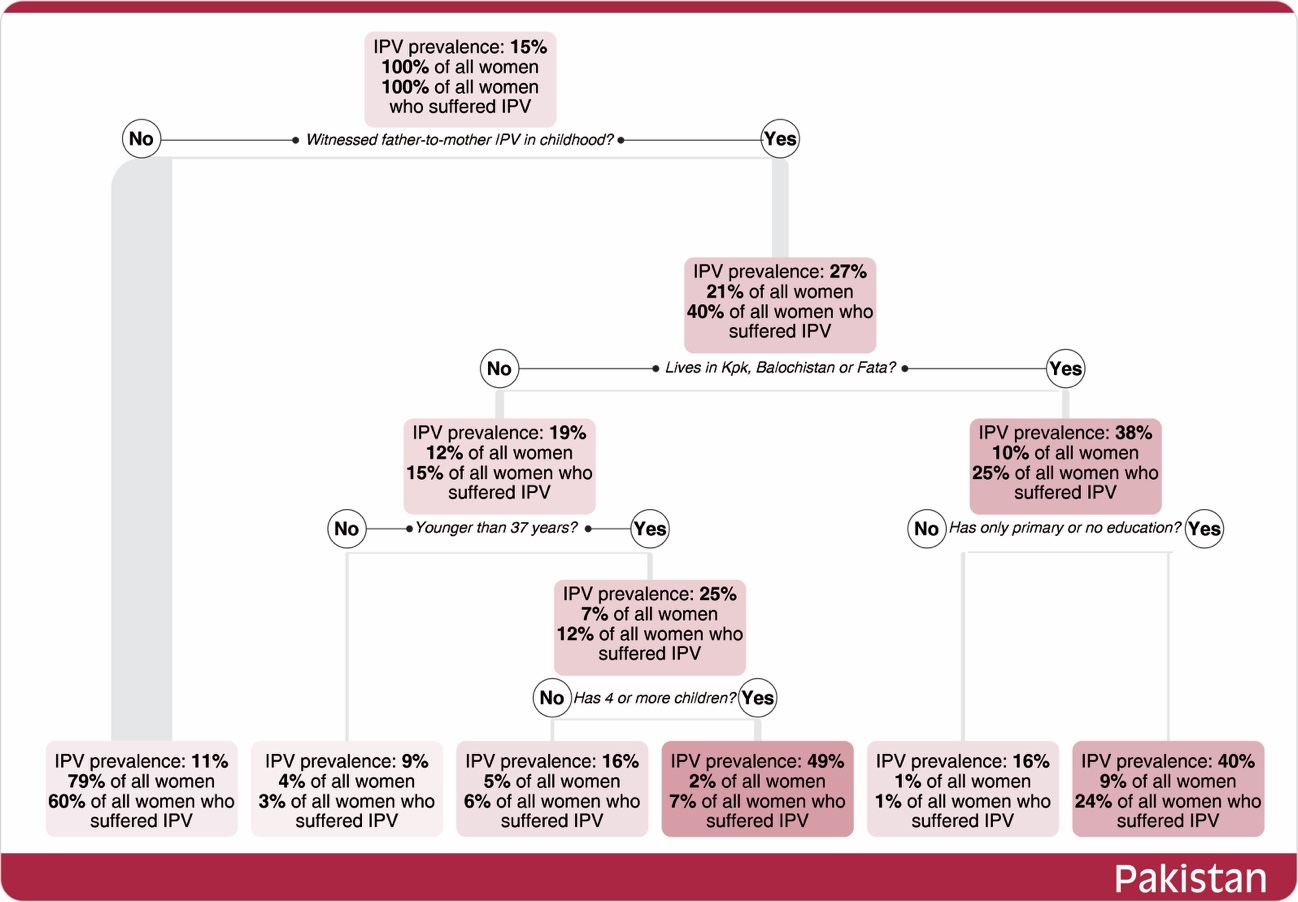


Figure S68 – Pakistan, 2017: decision tree


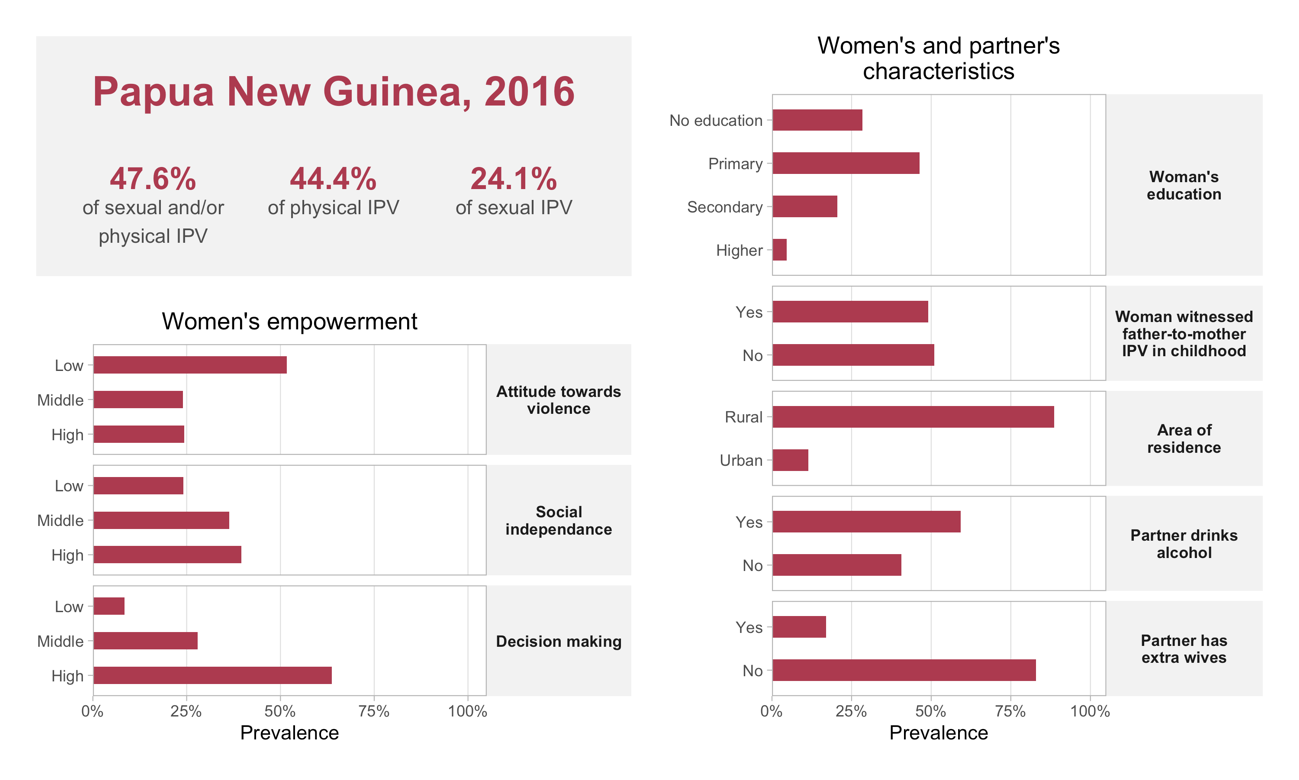
Figure S69 – Papua New Guinea, 2016: country profile


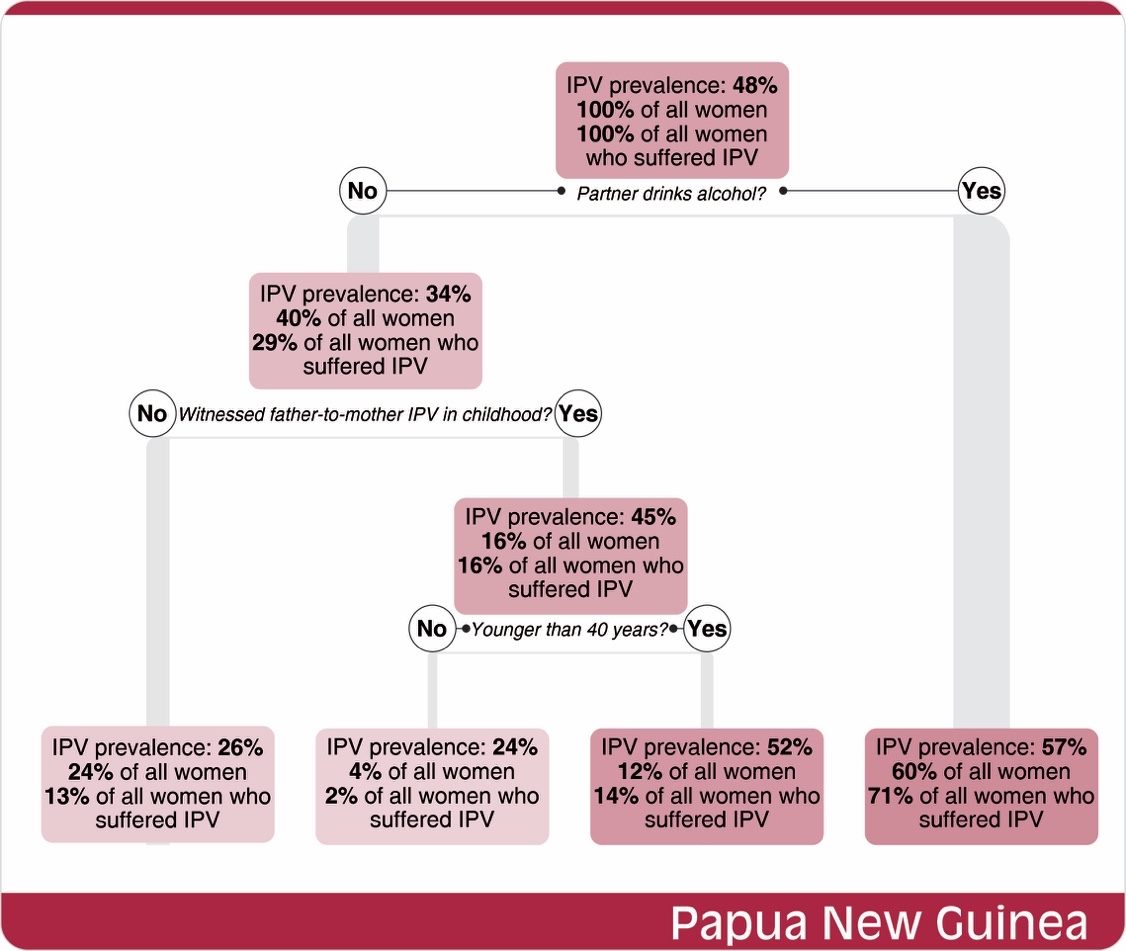


Figure S70 – Papua New Guinea, 2016: decision tree


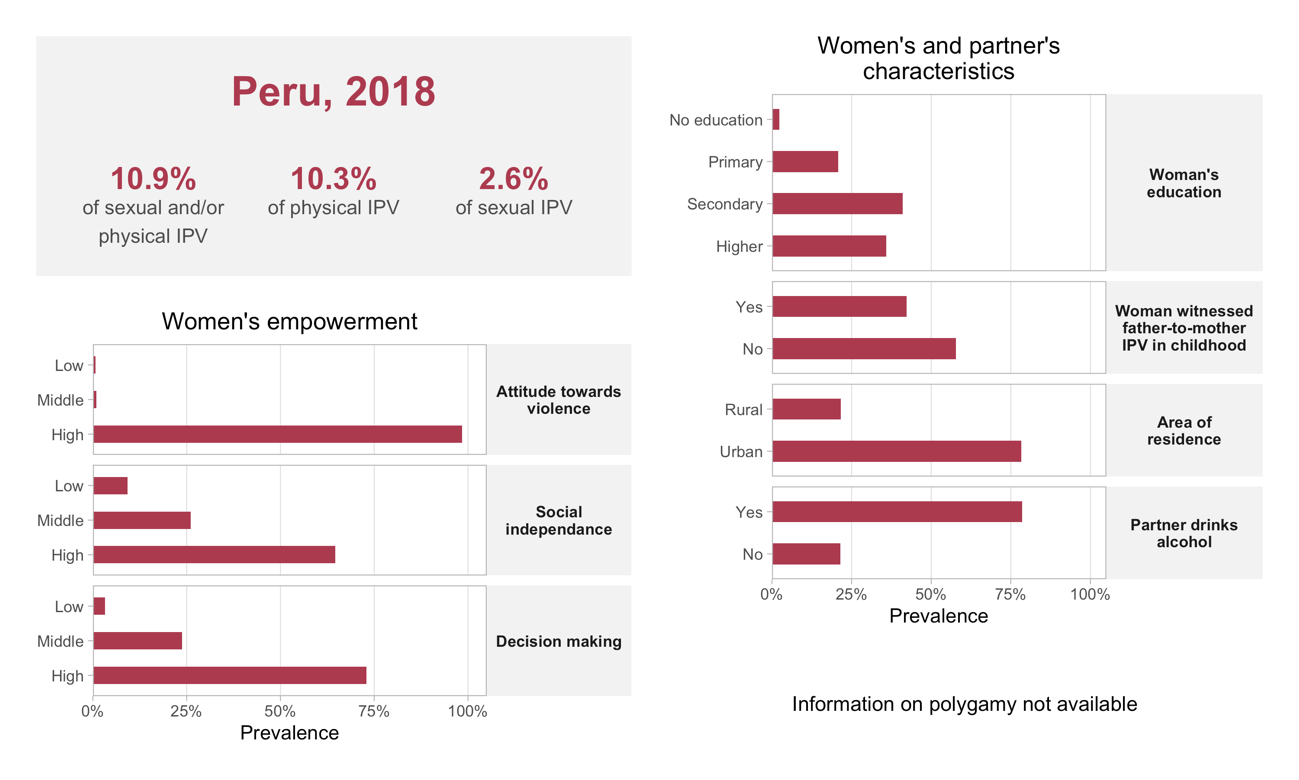
Figure S71 – Peru, 2018: country profile


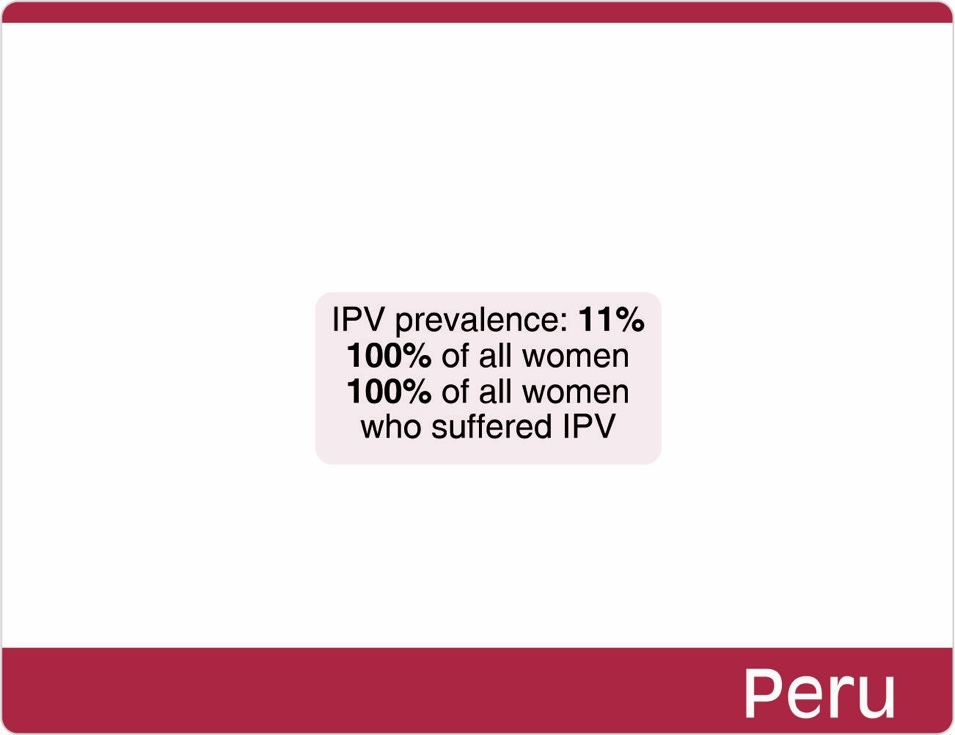


Figure S72 – Peru, 2018: decision tree


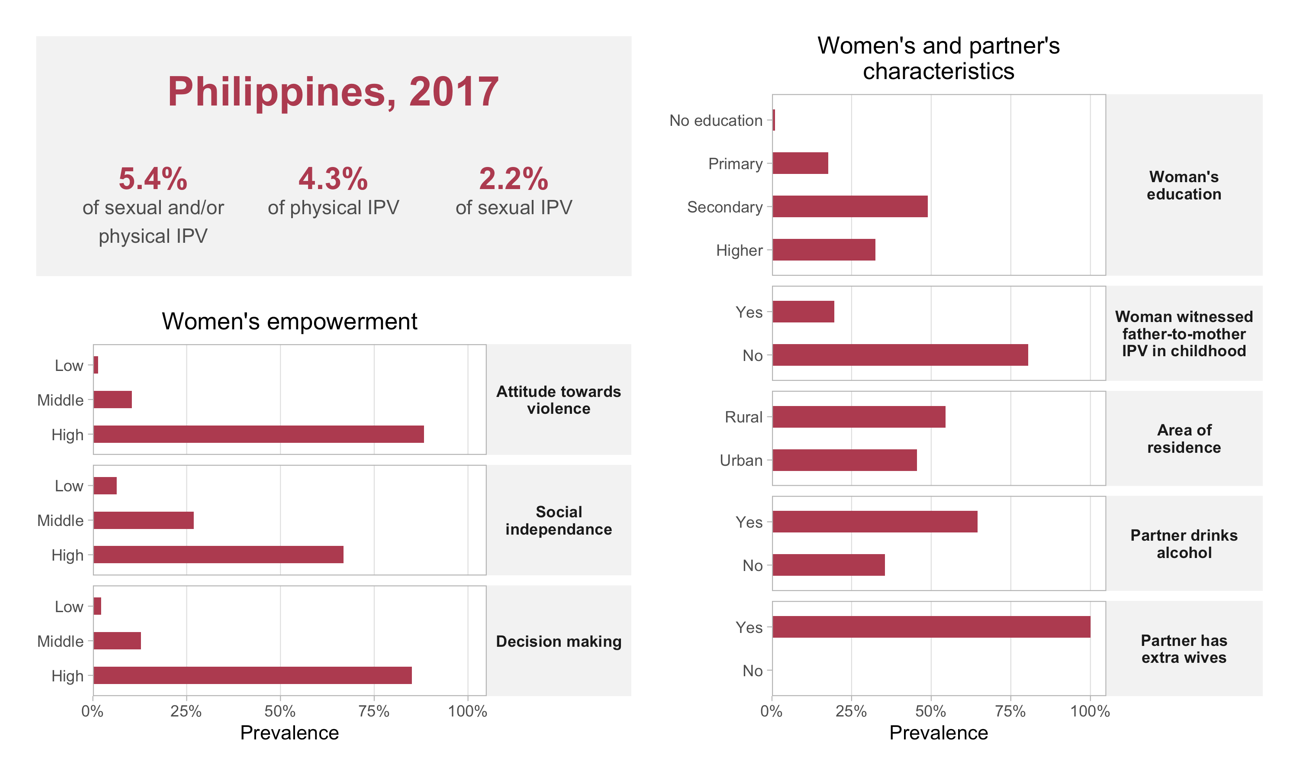
Figure S73 – Philippines, 2017: country profile


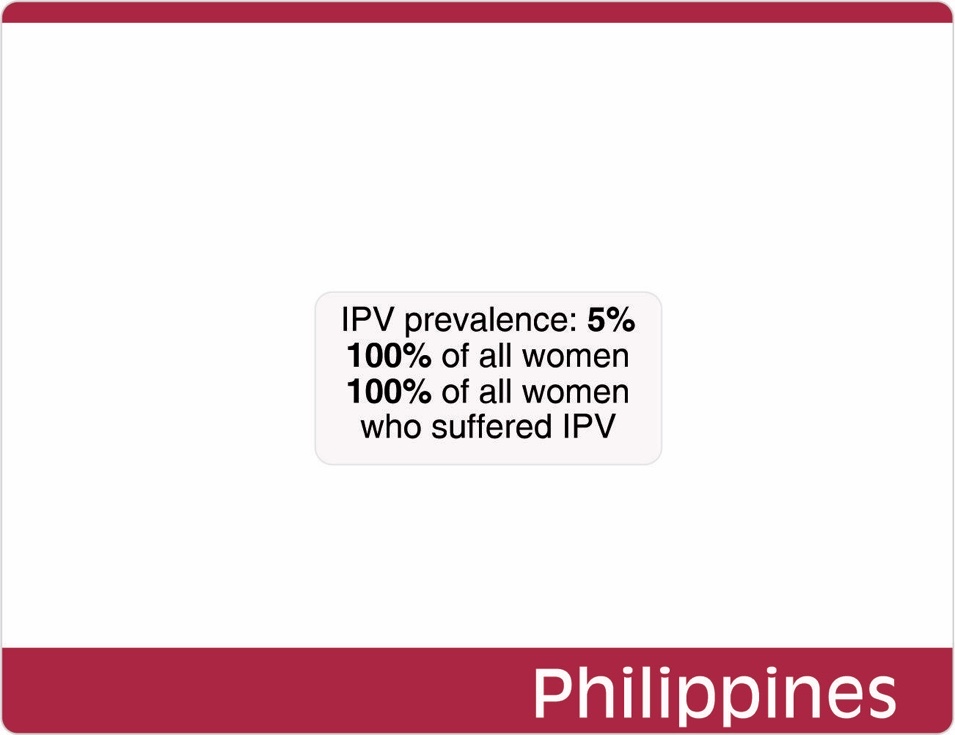


Figure S74 – Philippines, 2017: decision tree


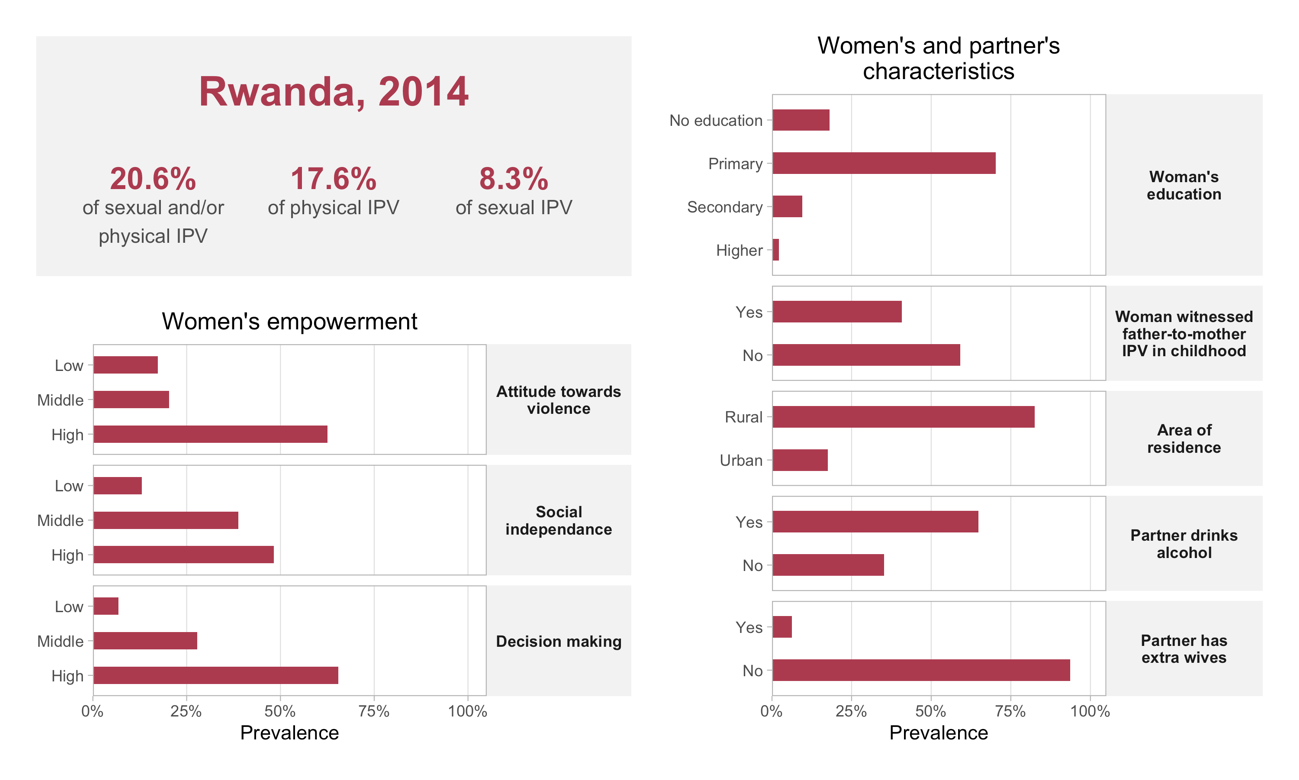
Figure S75 – Rwanda, 2014: country profile


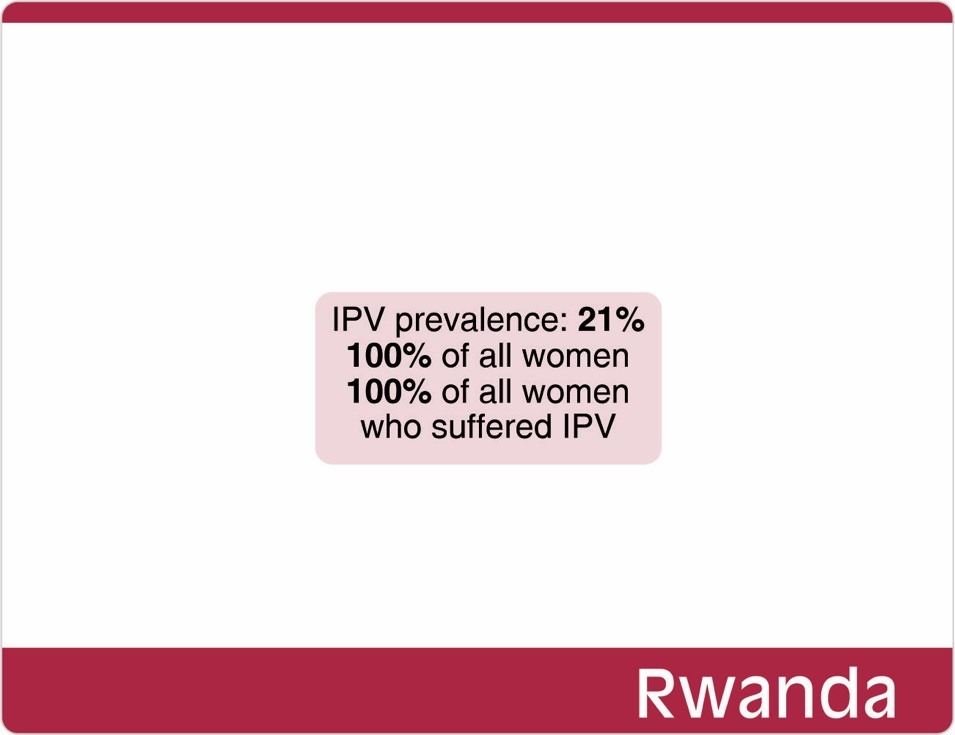


Figure S76 – Rwanda, 2014: decision tree


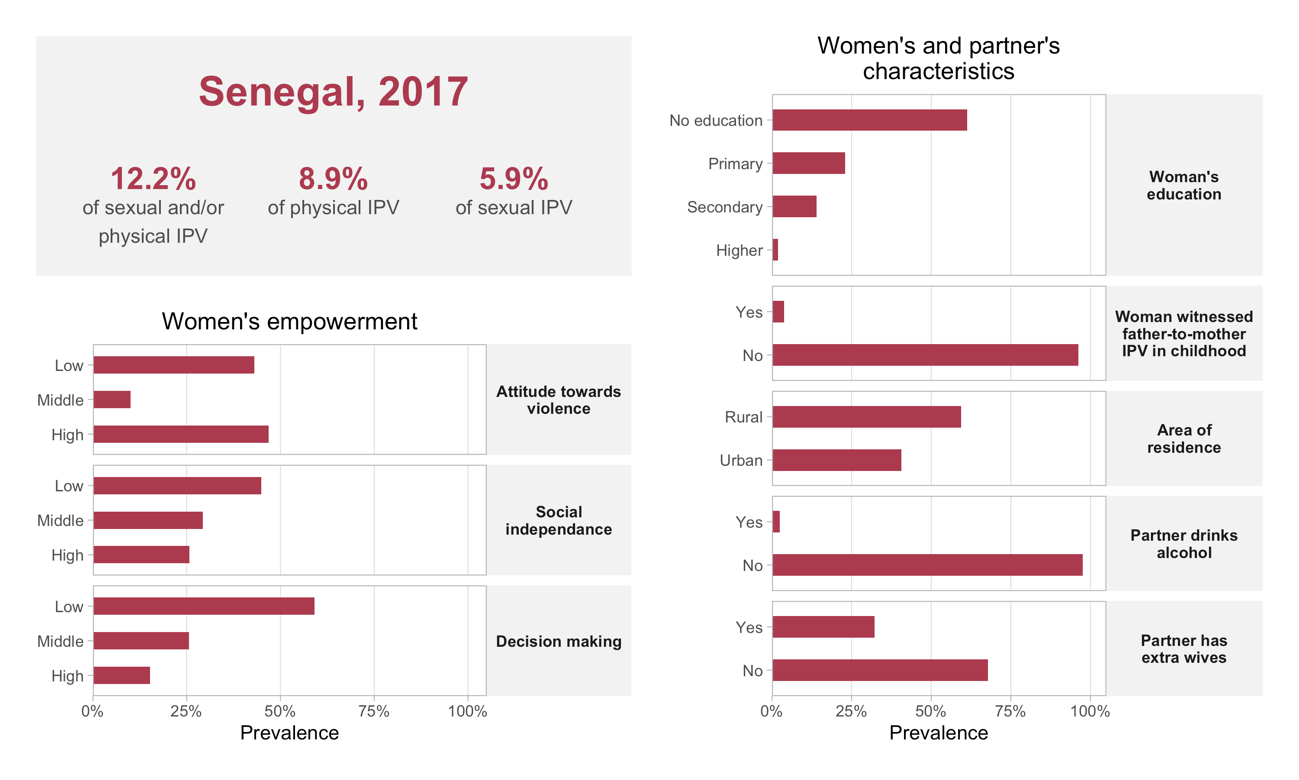
Figure S77 – Senegal, 2017: country profile


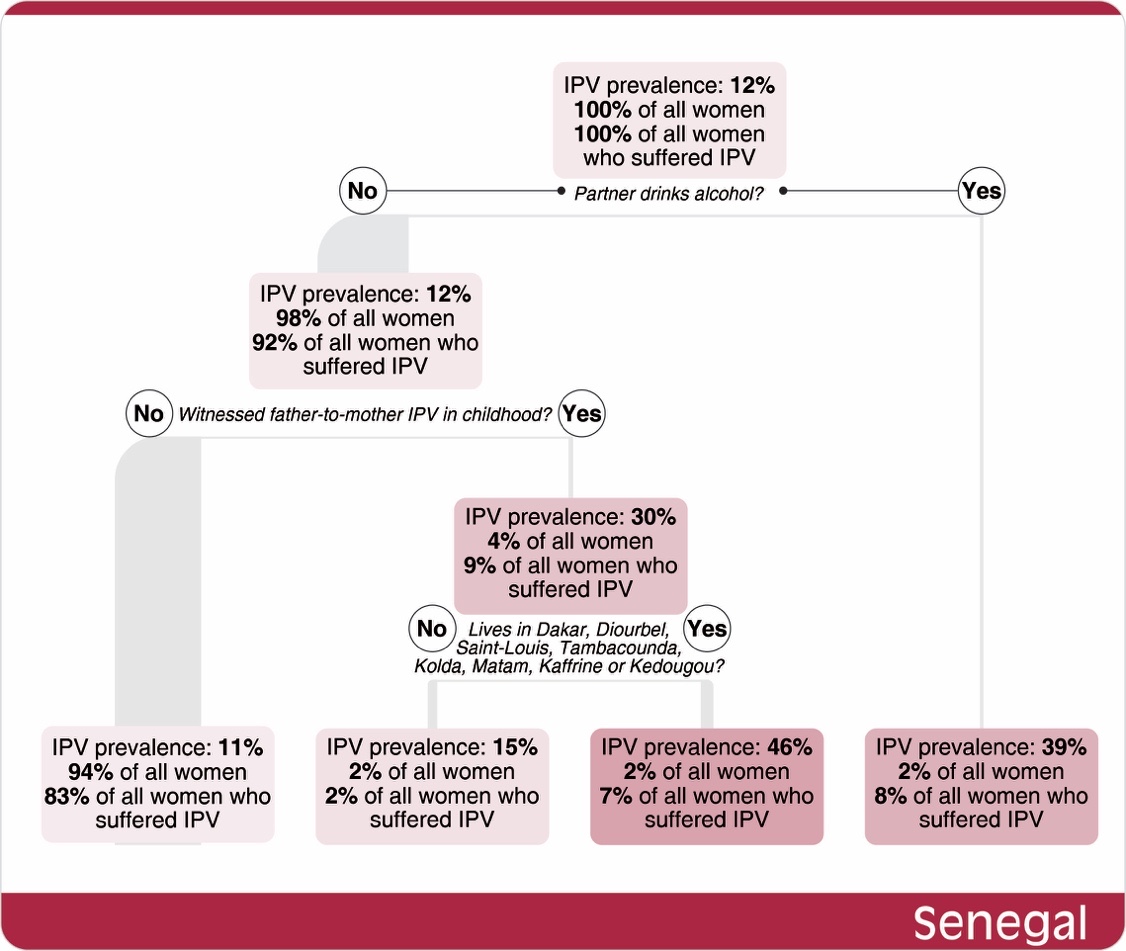


Figure S78 – Senegal, 2017: decision tree


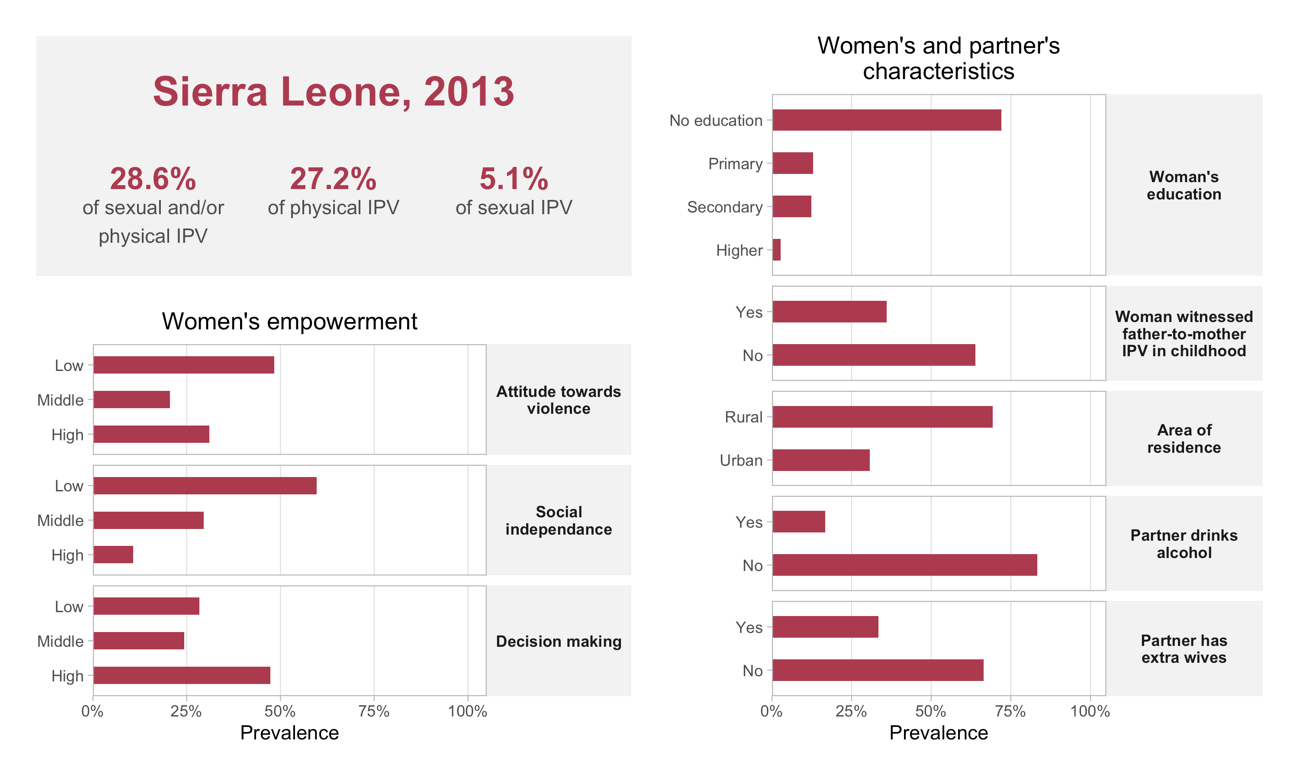
Figure S79 – Sierra Leone, 2013: country profile


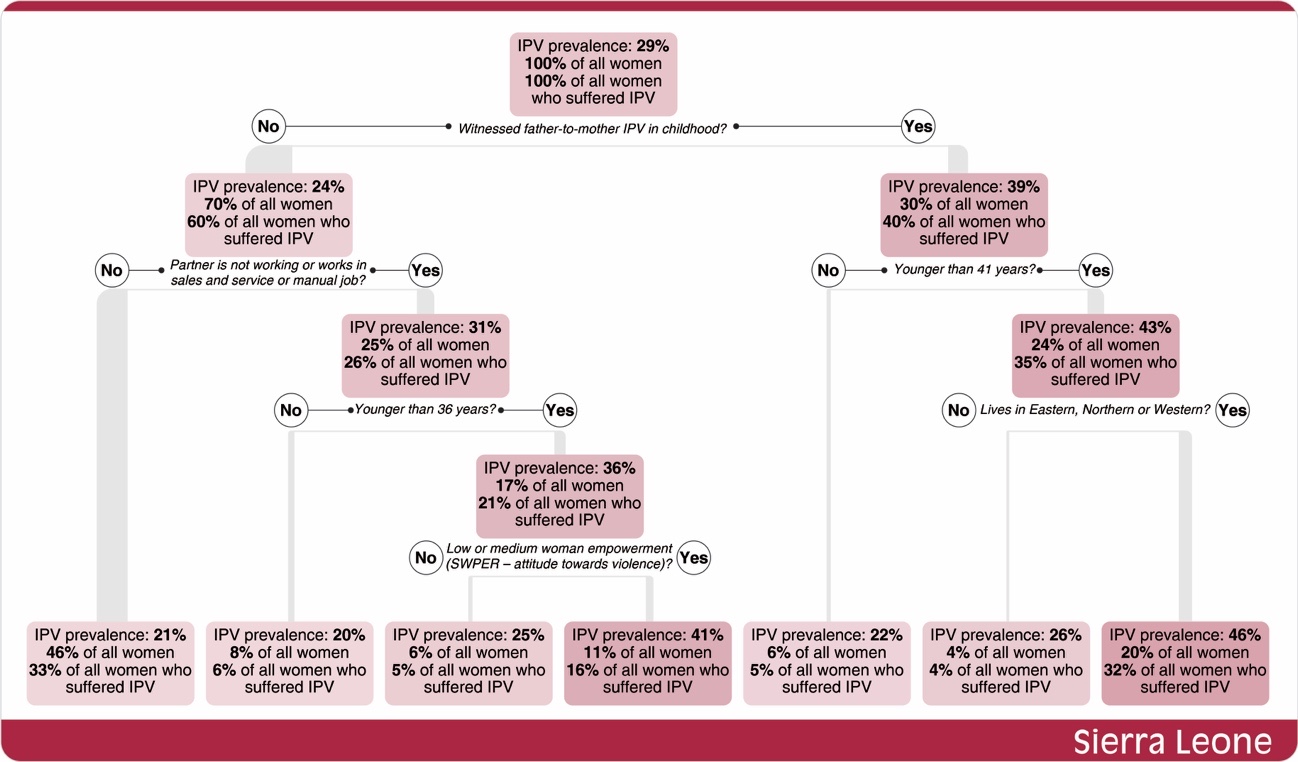


Figure S80 – Sierra Leone, 2013: decision tree


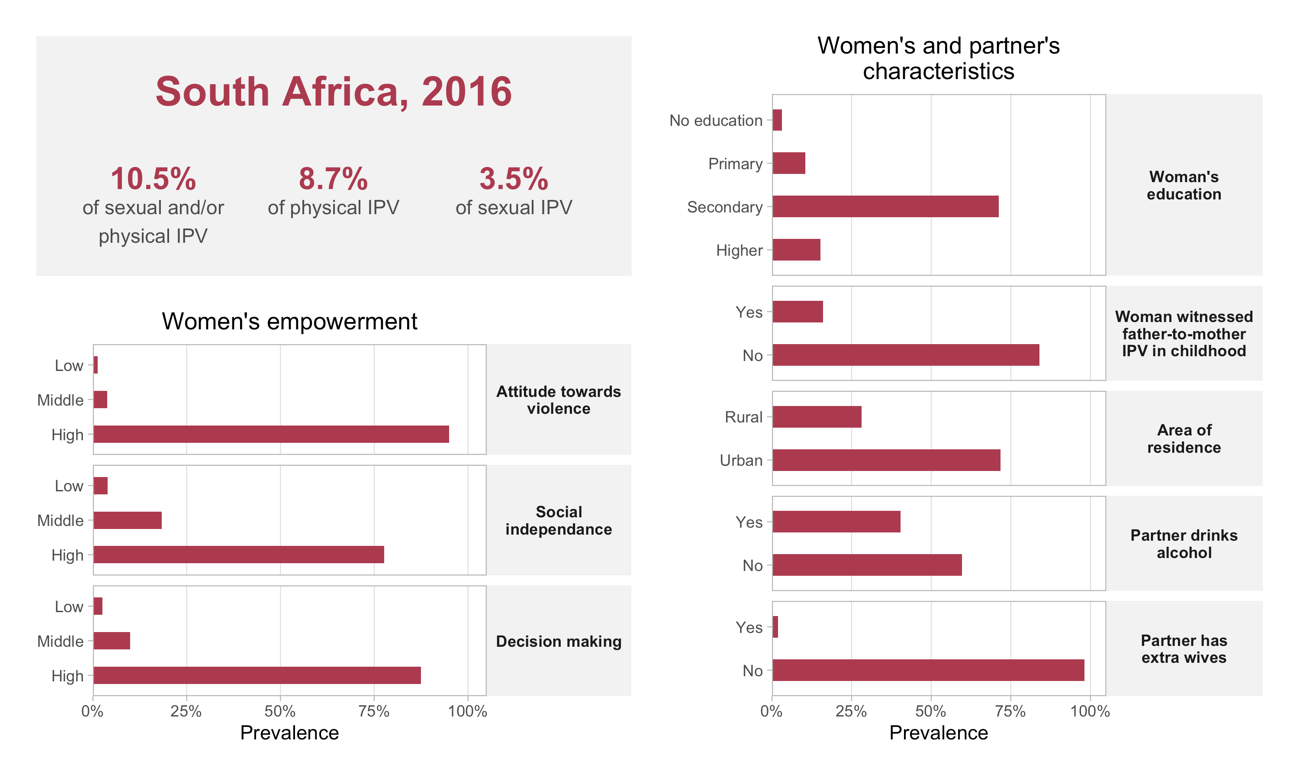
Figure S81 – South Africa, 2016: country profile


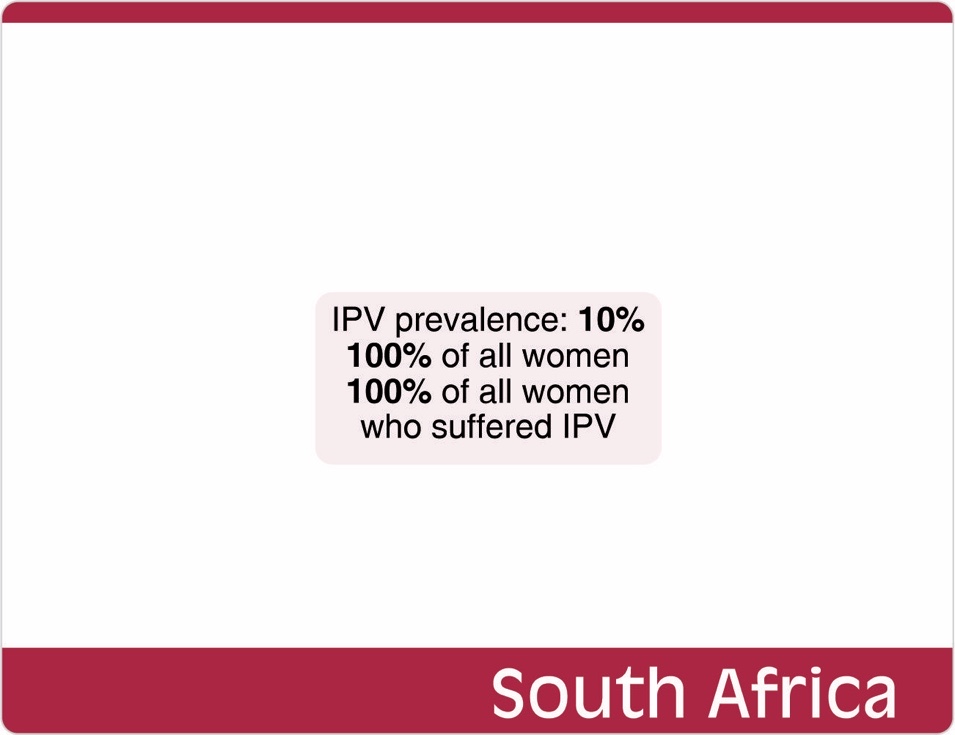


Figure S82 – South Africa, 2016: decision tree


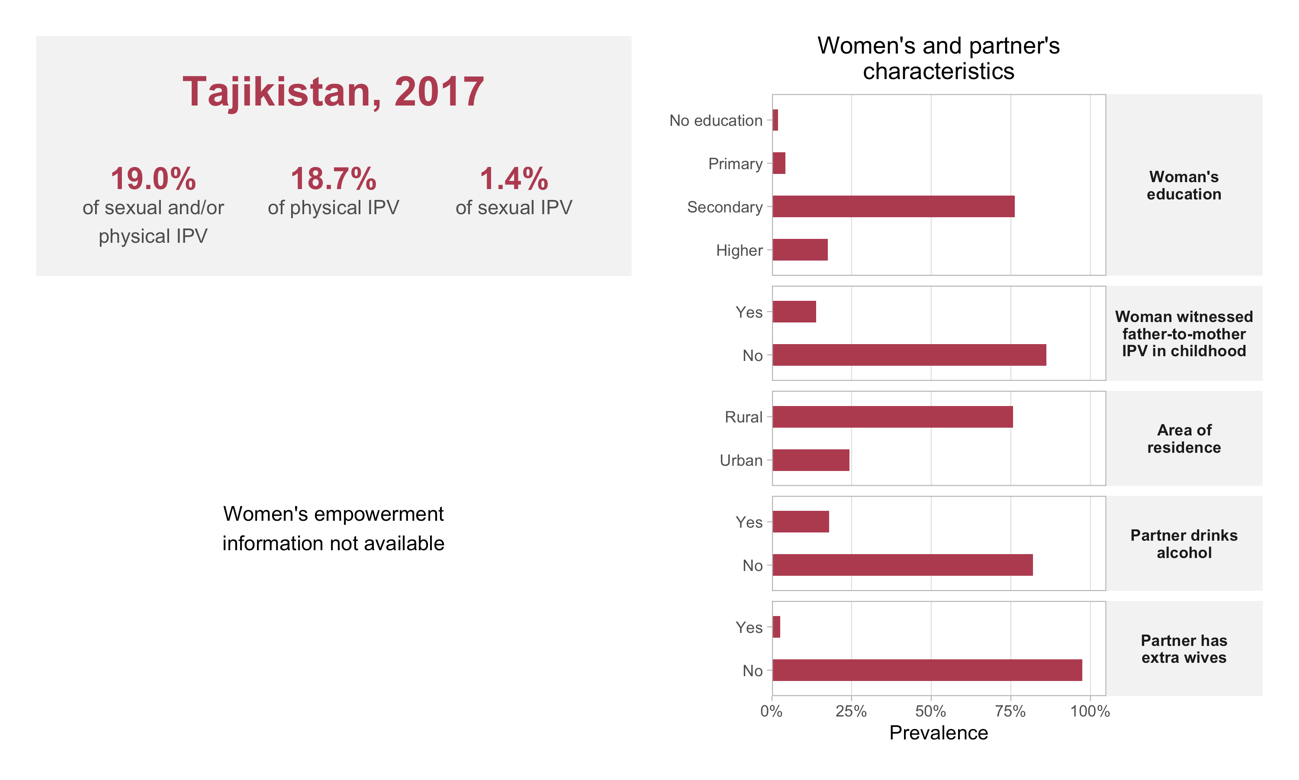
Figure S83 – Tajikistan, 2017: country profile


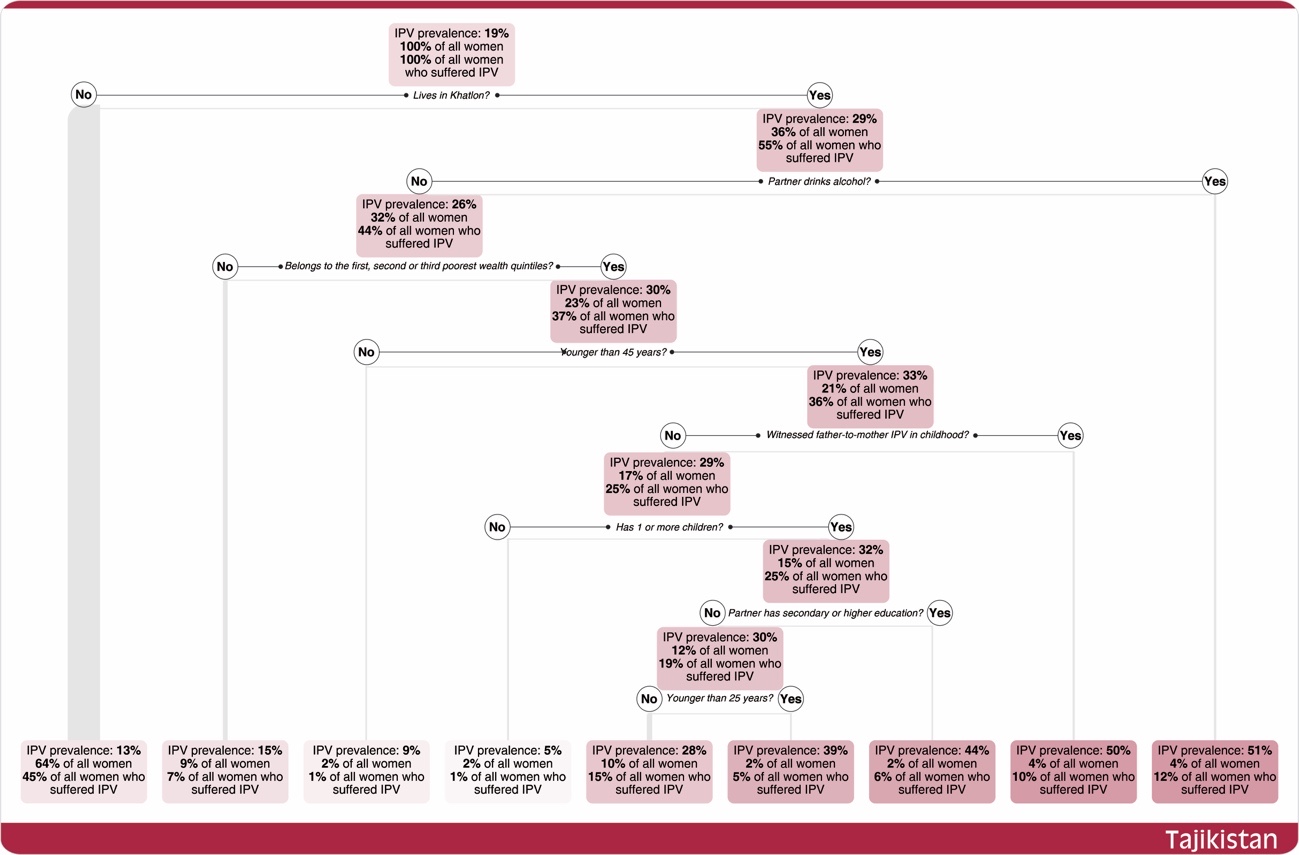


Figure S84 – Tajikistan, 2017: decision tree


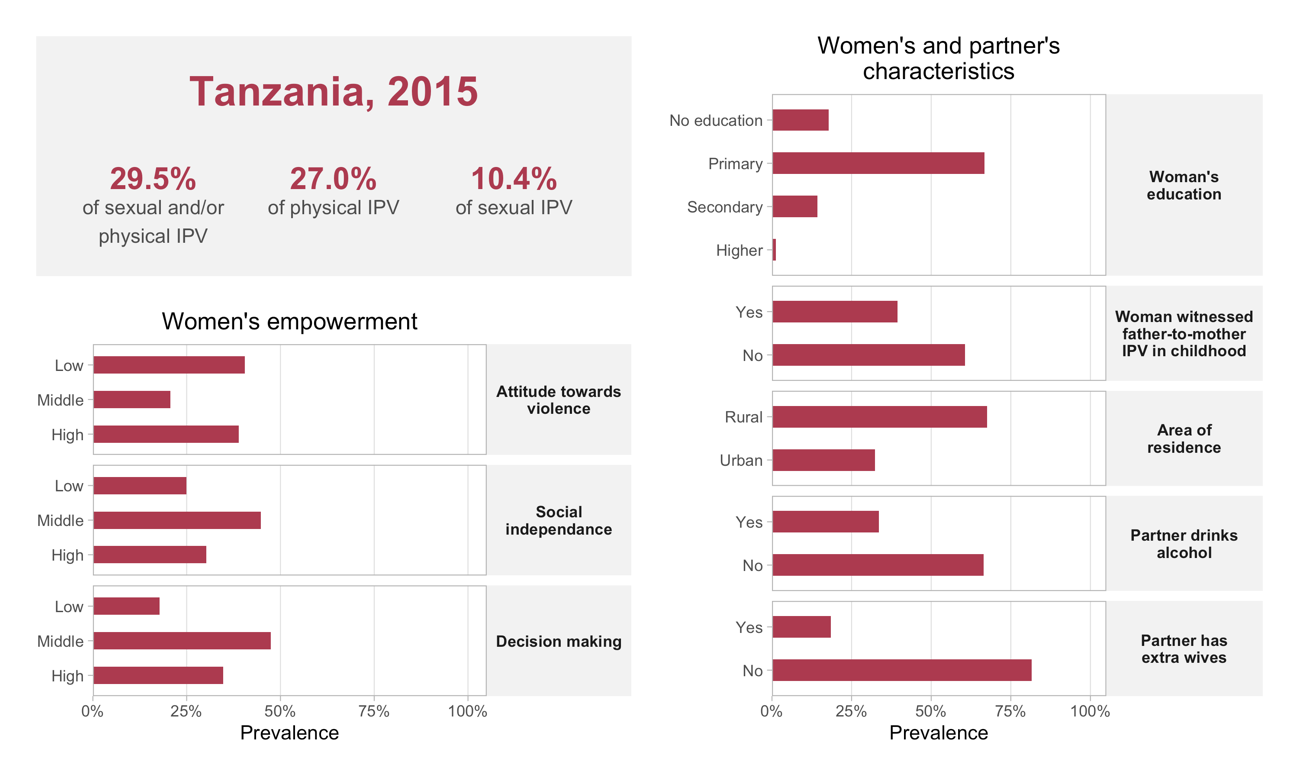
Figure S85 – Tanzania, 2015: country profile


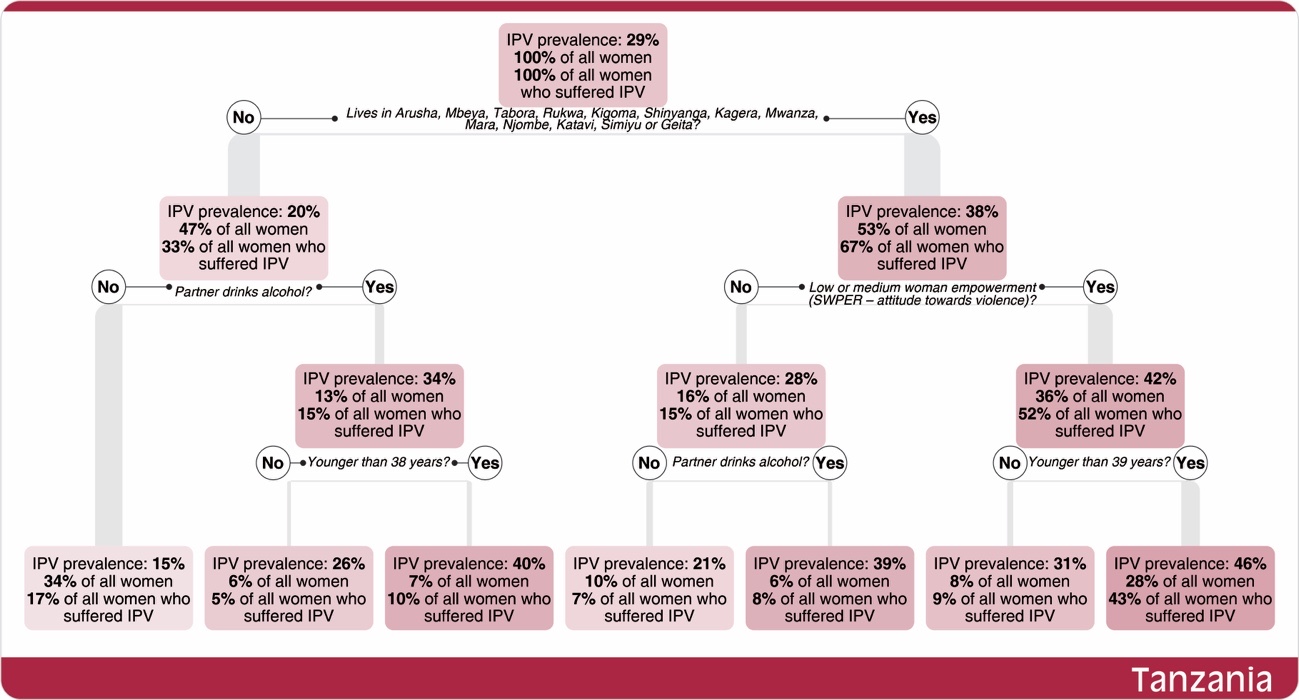


Figure S86 – Tanzania, 2015: decision tree


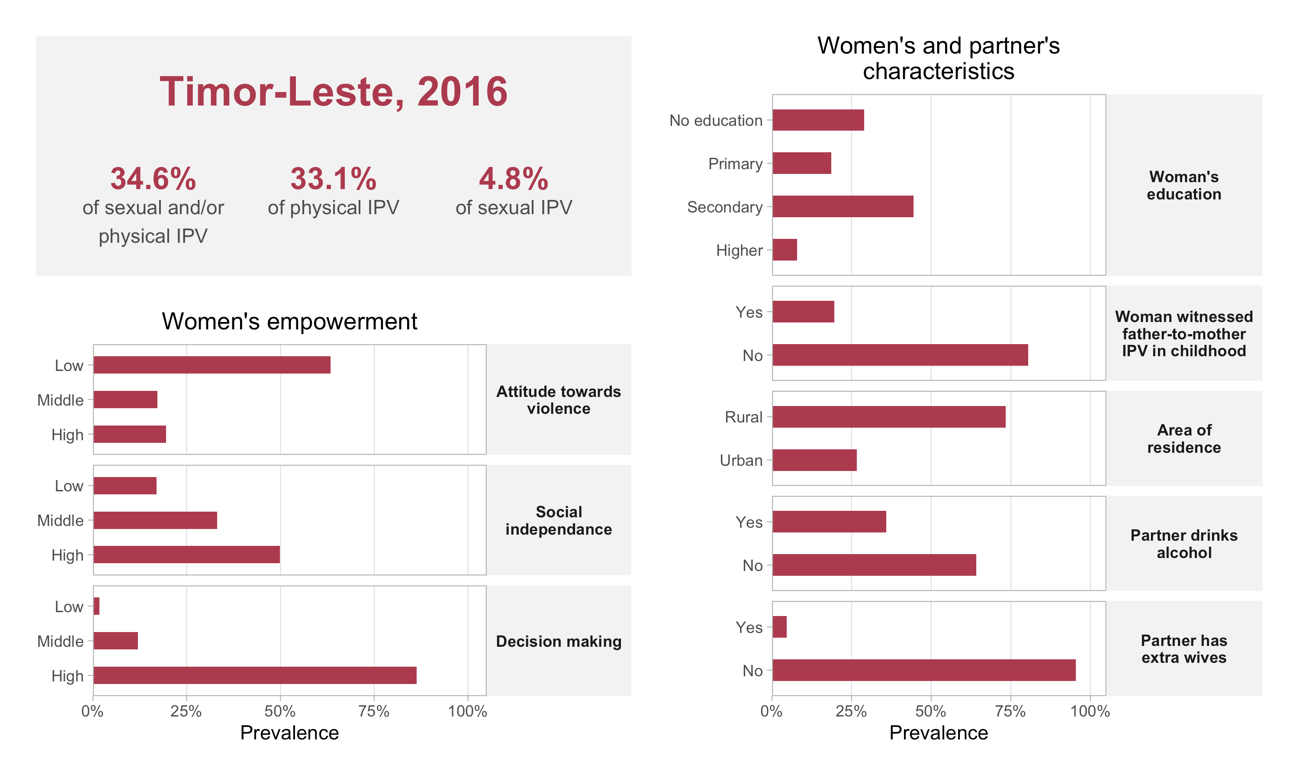
Figure S87 – Timor-Leste, 2016: country profile


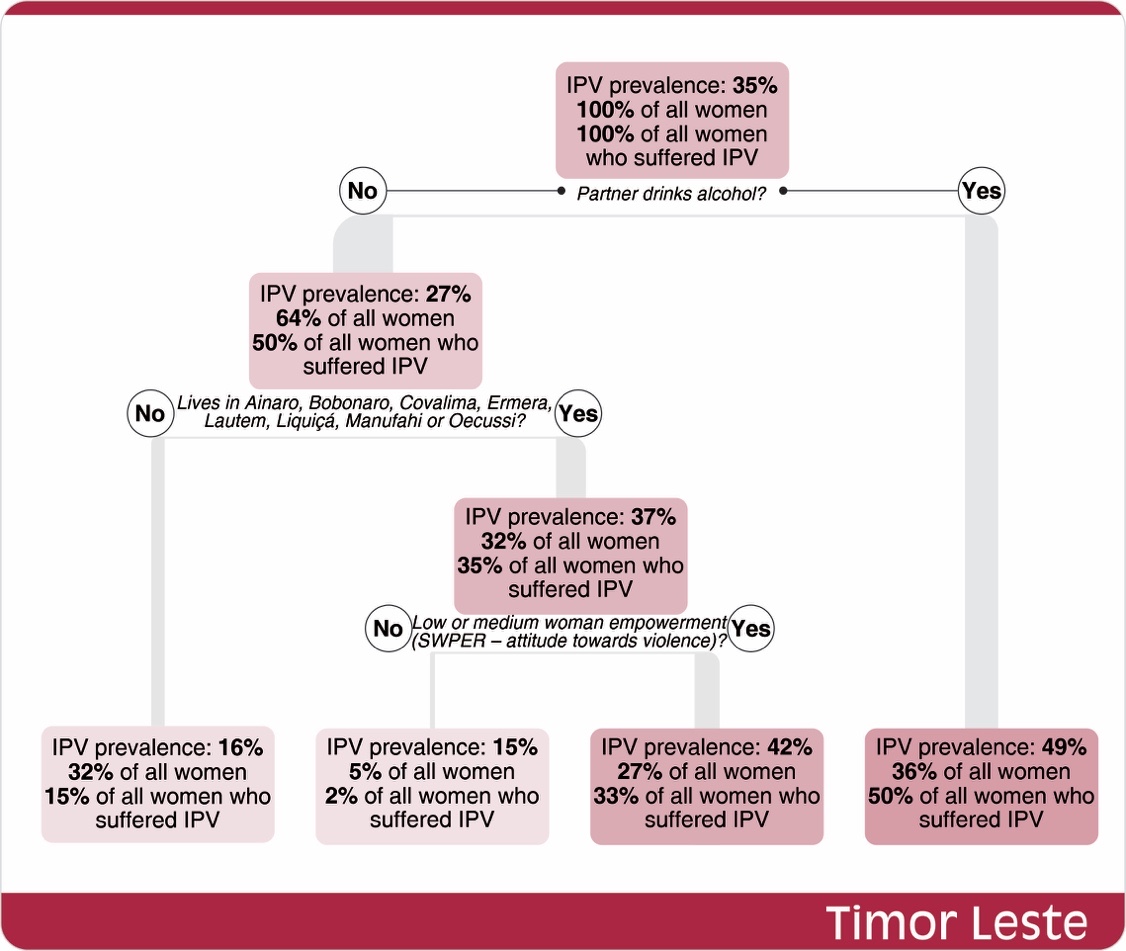


Figure S88 – Timor-Leste, 2016: decision tree


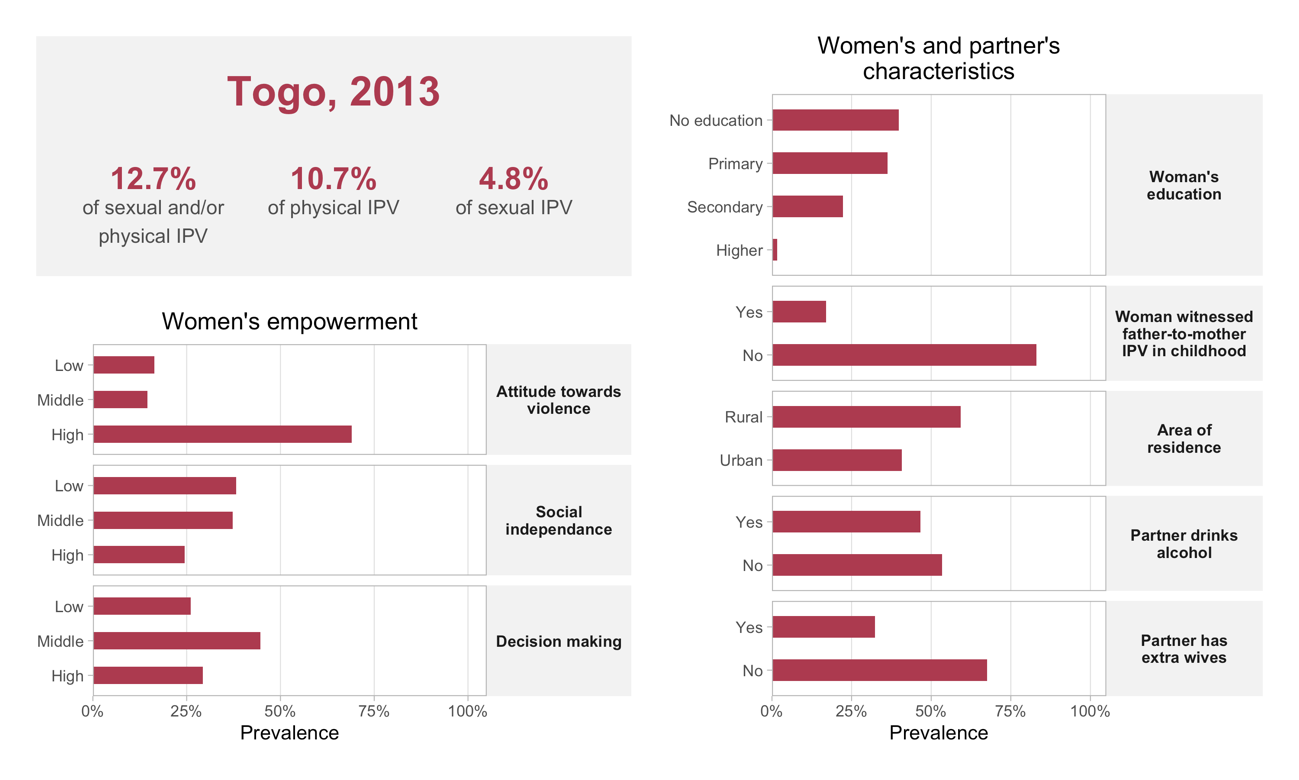
Figure S89 – Togo, 2013: country profile


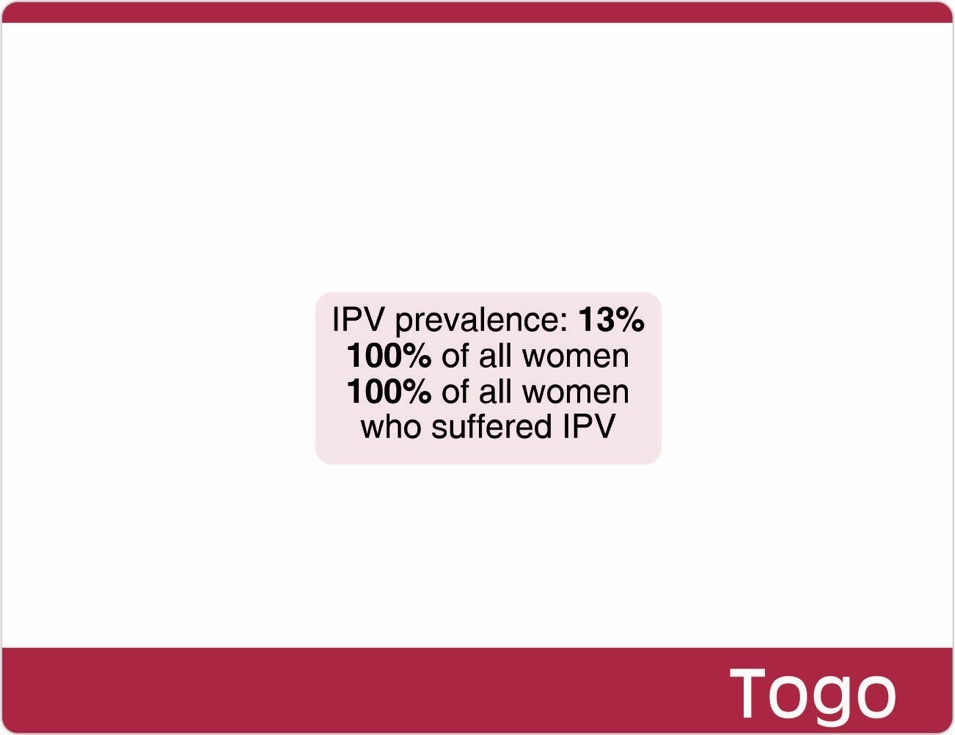


Figure S90 – Togo, 2013: decision tree


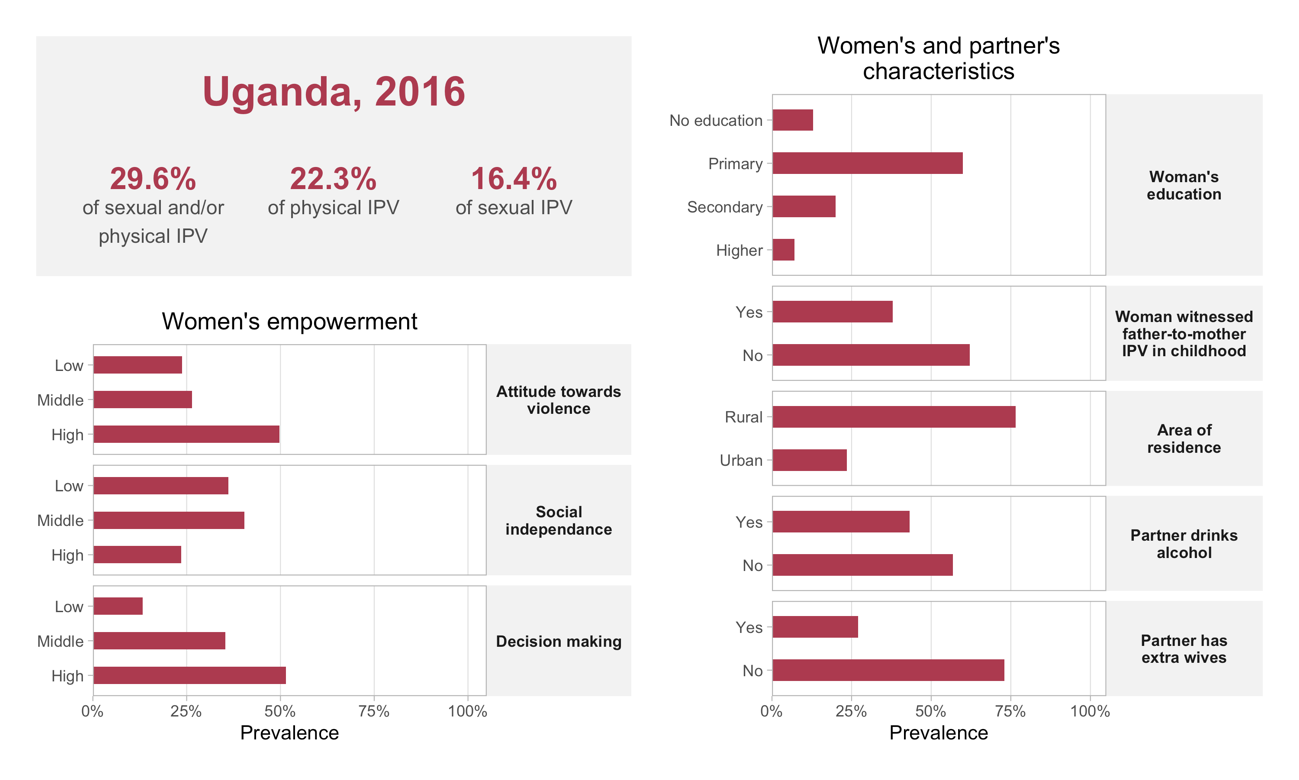
Figure S91 – Uganda, 2016: country profile


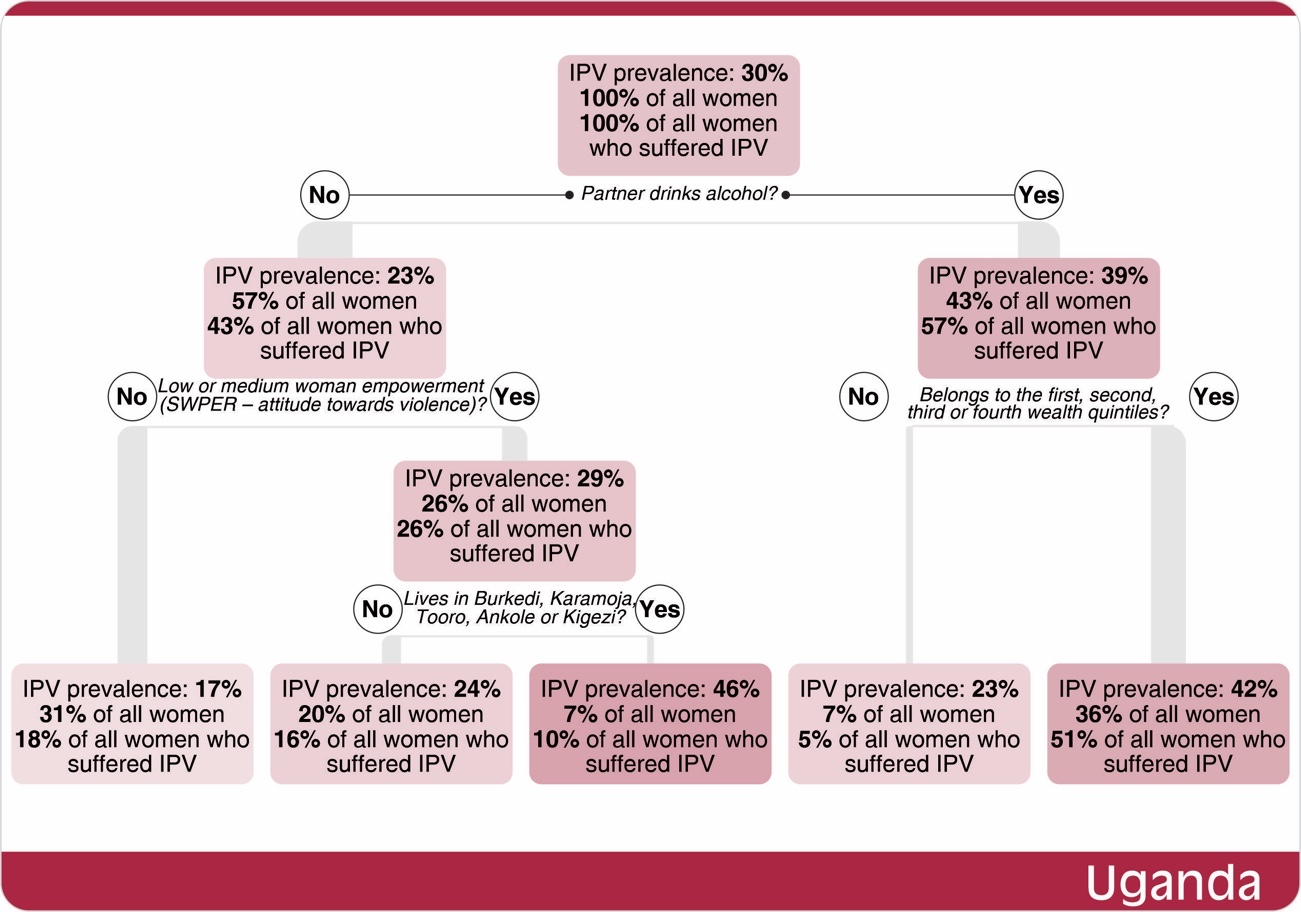


Figure S92 – Uganda, 2016: decision tree


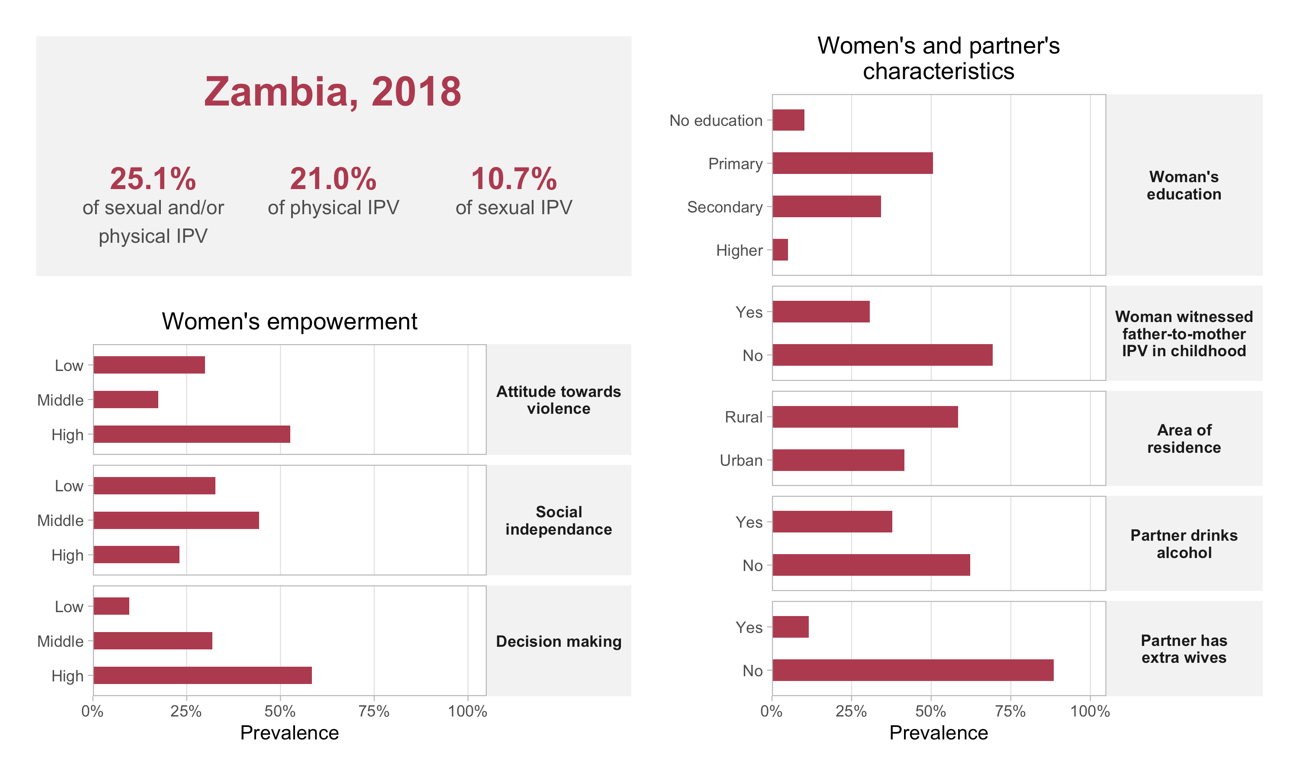
Figure S93 – Zambia, 2018: country profile


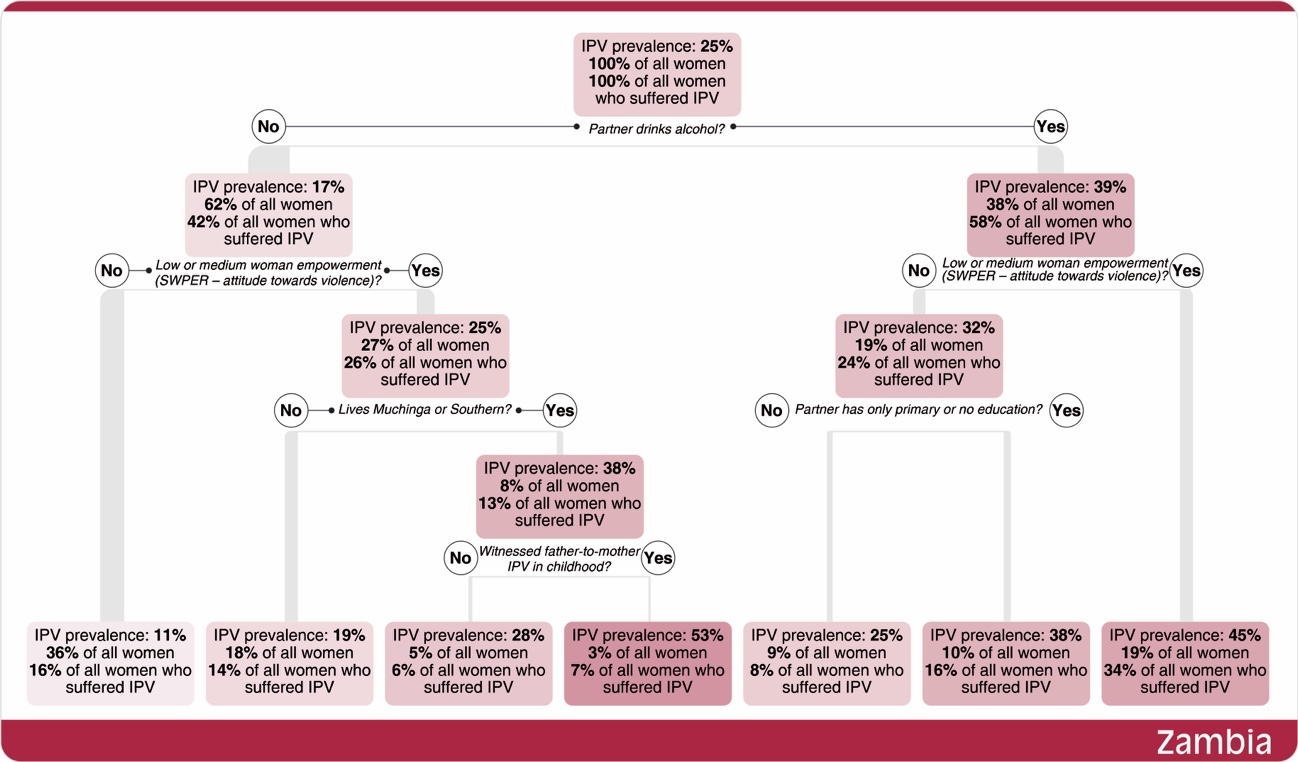


Figure S94 – Zambia, 2018: decision tree


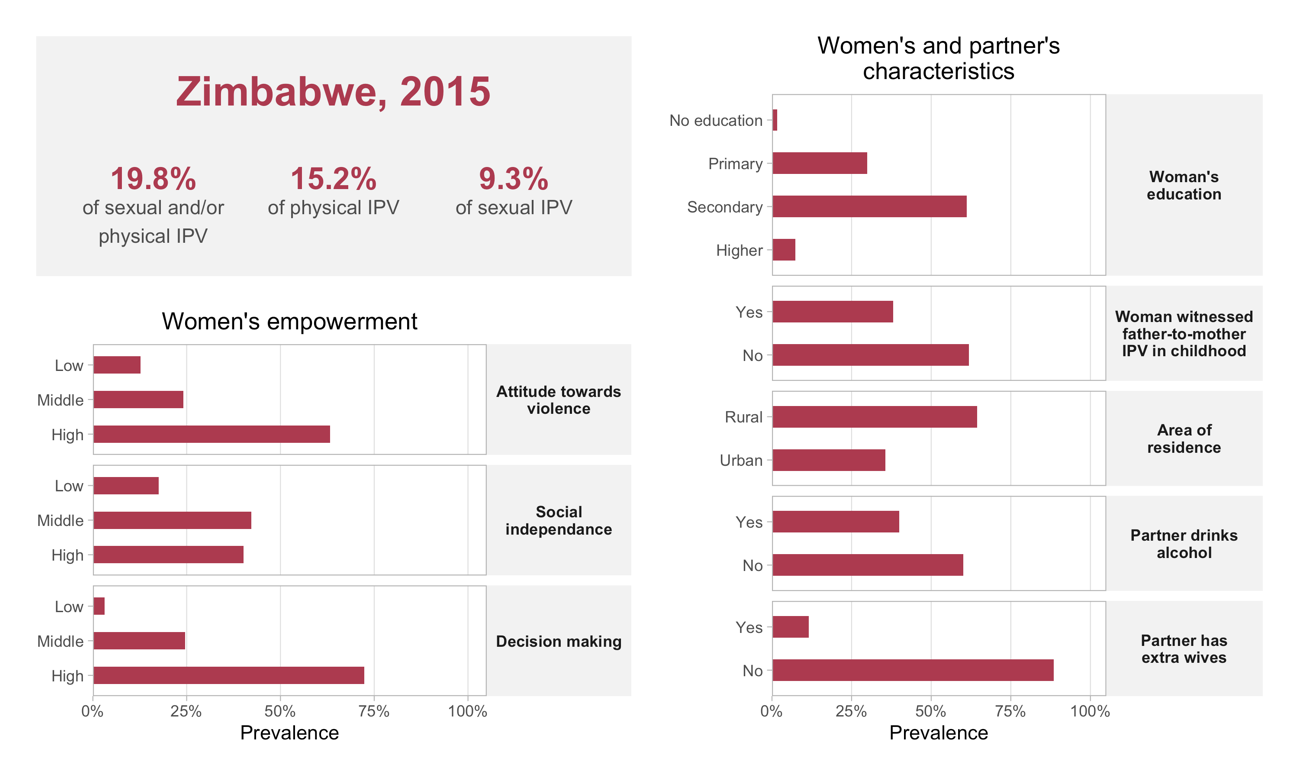
Figure S95 – Zimbabwe, 2015: f


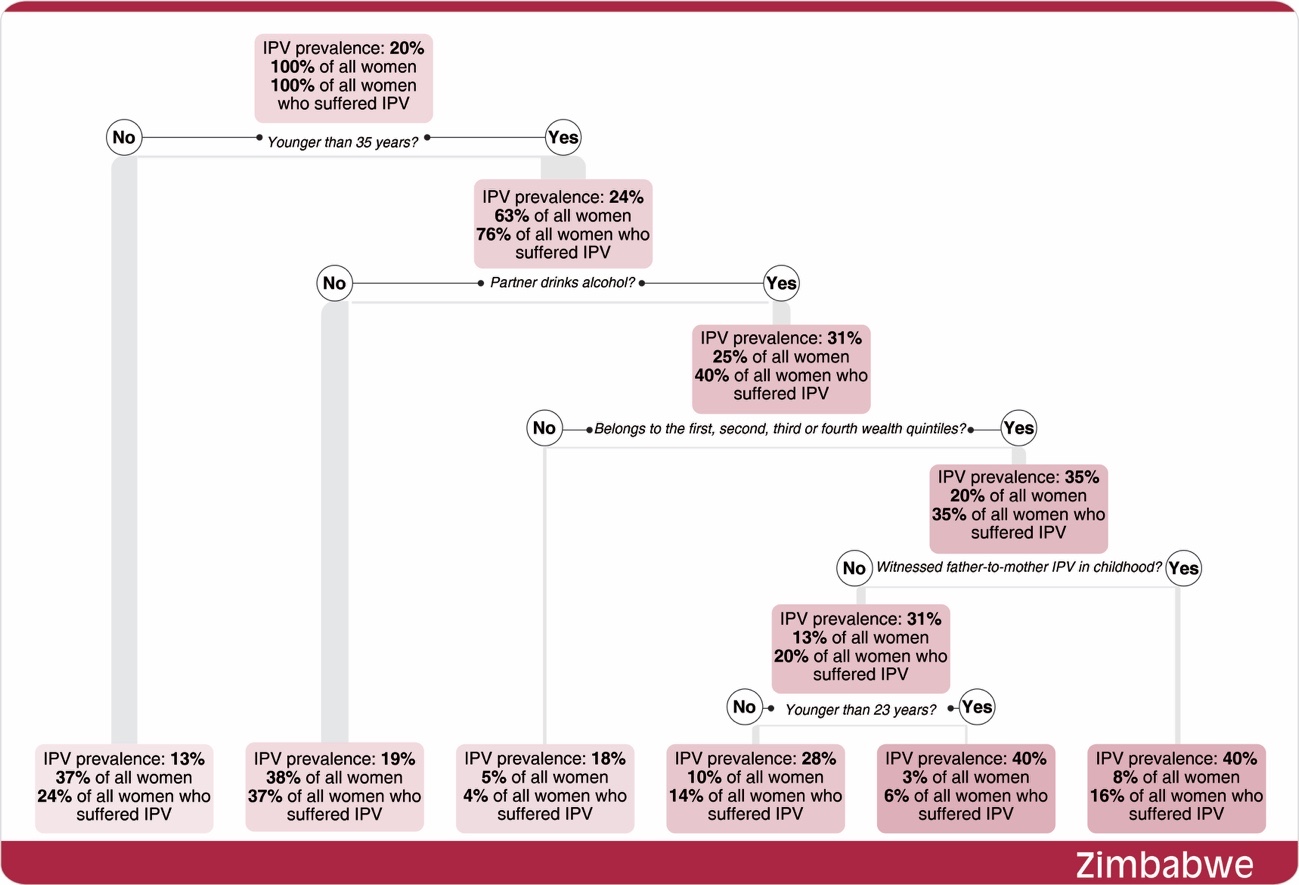


Figure S96 – Zimbabwe, 2015: decision tree

**CART specifications and sensitivity analysis**

By default, the rpart package uses what is called a “complexity parameter”, or cp, to determine a stopping rule: any split that does not decrease the tree’s lack of fit by a value of cp will not be attempted (Therneau et al., 2019). We used the default value of cp of 0.01, meaning splitting stopped if no split would improve the tree’s fit by 1% (Venkatasubramaniam et al., 2017). We used the Gini index as a measurement of node homogeneity and used the standard maximum depth (number of splits) (Therneau & Atkinson, 2019) for any node in the final tree of 30.

In order to evaluate the tree’s sensitivity to its parameters’ values, we have tested 24 different scenarios, varying the following parameters, while maintaining the others constant:

- **Minimum number of women in a node:** 0, 10, 25, **50 (reference)**, 100, 200, and 500
- **Misclassification cost:** 0.5, 1, 1.5, **2 (reference)**, 2.5, 3, and 3.5
- **Complexity parameter:** 0.002, 0.005, 0.0067, **1 (reference)**, 0.015, 0.02, and 0.05
- **Maximum depth:** 10, 20, 25, **30 (reference)**, 35, 40, and 50

The results are presented in Table S6 and Figures S97 to S108. Since tests 1 to 6 and 19 to 24 give the same pooled tree as the one presented in the main text, they are not shown in Figures.

As expected, given the large sample size, the minimum number of women in a node has no effect on the pooled tree and tests 1 to 6 give the same results (Table S6).

For the misclassification cost, using weights 0.5 and 1 (tests 7 and 8) creates trees with no splits. The tree is simply classifying every woman as “not having experienced IPV” and having high accuracy, but 0% sensitivity. This is due to the unbalanced sample. From tests 9 to 12, splits are performed, and the trees use the same variables: whether the woman witnessed father-to-mother IPV in childhood, the woman’s empowerment (attitude to violence) and the partner’s alcohol use. Test 12 also include the social independence domain of empowerment. Although the variables select are consistent, their structure in the tree changes (Figures S97 to S102), especially the partner’s alcohol use.

Varying the complexity parameter has the expected result: the larger its value, the shorter is the tree (Table S6 and Figures S103 to S108). This is due to the fact that the complexity parameter behaves as the main stopping rule of the tree. Larger values (like test 18, cp = 0.05) require each split to improve the tree’s fit by a larger amount, therefore preventing more splits and creating shorter trees (only 3 nodes and one split in test 18). One can think of the complexity parameter as analogous to the p value used for variable selection in a linear regression analysis. A more restrictive p value – i.e., a smaller one – will reduce the number of variables in the final regression model. A more restrictive complexity parameter – i.e., a larger one – will reduce the number of nodes in the final tree. And as with the p-value, the result will be highly sensitive to its choice.

We believe that the standard value of 0.01, or 1%, is an adequate choice in this scenario. Smaller values will create trees with many splits, possibly allowing for overfitting, the creation of many small subgroups and groups with similar IPV prevalence, reducing interpretability. This also resulted in many smaller groups. Larger values will be very restrictive and prevent subgroups identification. Nevertheless, it is important to notice that – as noticed in the misclassification cost analysis – the partner’s alcohol use is the most sensitive indicator and should be interpreted with caution.

Finally, the maximum depth had no effect on the pooled tree and tests 19 to 24 give the same results (Table S6).

Therneau, T., & Atkinson, B. (2019). *rpart: Recursive Partitioning and Regression Trees*.

Therneau, T., Atkinson, B., & Ripley, B. (2019). rpart: Recursive partitioning for classification, regression and survival trees. *CRAN R Package Version 4.1-15*. https://cran.r-project.org/package=rpart

Venkatasubramaniam, A., Wolfson, J., Mitchell, N., Barnes, T., Jaka, M., & French, S. (2017). Decision trees in epidemiological research. In *Emerging Themes in Epidemiology* (Vol. 14, Issue 1). https://doi.org/10.1186/s12982-017-0064-4

**Table S6 – Sensitivity Analysis**

| **Test** | **Minimum n of women in a node** | **Misclassification cost** | **Complexity parameter** | **Maximum depth** | **Number of nodes** | **Witnessed father-to-mother IPV in childhood** | **Woman’s empowerment – attitude to violence** | **Partner’s alcohol use** | **Religious affiliation** | **Wealth quintiles** | **Woman’s empowerment – decision making** | **Woman’s empowerment – social independence** | **Area of residence (urban/rural)** | **Number of living children** | **Partner’s education** | **Partner’s occupation** | **Polygamy** | **Woman’s age** | **Woman’s education** |
| --- | --- | --- | --- | --- | --- | --- | --- | --- | --- | --- | --- | --- | --- | --- | --- | --- | --- | --- | --- |
| **Reference** | **50** | **2** | **0.01** | **30** | **7** | **✓** | **✓** | **✓** |  |  |  |  |  |  |  |  |  |  |  |
| 1 | 0 | 2 | 0.01 | 30 | 7 | ✓ | ✓ | ✓ |  |  |  |  |  |  |  |  |  |  |  |
| 2 | 10 | 2 | 0.01 | 30 | 7 | ✓ | ✓ | ✓ |  |  |  |  |  |  |  |  |  |  |  |
| 3 | 25 | 2 | 0.01 | 30 | 7 | ✓ | ✓ | ✓ |  |  |  |  |  |  |  |  |  |  |  |
| 4 | 100 | 2 | 0.01 | 30 | 7 | ✓ | ✓ | ✓ |  |  |  |  |  |  |  |  |  |  |  |
| 5 | 200 | 2 | 0.01 | 30 | 7 | ✓ | ✓ | ✓ |  |  |  |  |  |  |  |  |  |  |  |
| 6 | 500 | 2 | 0.01 | 30 | 7 | ✓ | ✓ | ✓ |  |  |  |  |  |  |  |  |  |  |  |
| 7 | 50 | 0.5 | 0.01 | 30 | 1 |  |  |  |  |  |  |  |  |  |  |  |  |  |  |
| 8 | 50 | 1 | 0.01 | 30 | 1 |  |  |  |  |  |  |  |  |  |  |  |  |  |  |
| 9 | 50 | 1.5 | 0.01 | 30 | 7 | ✓ | ✓ | ✓ |  |  |  |  |  |  |  |  |  |  |  |
| 10 | 50 | 2.5 | 0.01 | 30 | 11 | ✓ | ✓ | ✓ |  |  |  |  |  |  |  |  |  |  |  |
| 11 | 50 | 3 | 0.01 | 30 | 7 | ✓ | ✓ | ✓ |  |  |  |  |  |  |  |  |  |  |  |
| 12 | 50 | 3.5 | 0.01 | 30 | 9 | ✓ | ✓ | ✓ |  |  |  | ✓ |  |  |  |  |  |  |  |
| 13 | 50 | 2 | 0.002 | 30 | 19 | ✓ | ✓ | ✓ | ✓ | ✓ | ✓ |  |  |  |  |  |  |  |  |
| 14 | 50 | 2 | 0.005 | 30 | 15 | ✓ | ✓ | ✓ | ✓ | ✓ |  |  |  |  |  |  |  |  |  |
| 15 | 50 | 2 | 0.0067 | 30 | 9 | ✓ | ✓ | ✓ | ✓ |  |  |  |  |  |  |  |  |  |  |
| 16 | 50 | 2 | 0.015 | 30 | 5 | ✓ | ✓ |  |  |  |  |  |  |  |  |  |  |  |  |
| 17 | 50 | 2 | 0.02 | 30 | 5 | ✓ | ✓ |  |  |  |  |  |  |  |  |  |  |  |  |
| 18 | 50 | 2 | 0.05 | 30 | 3 | ✓ |  |  |  |  |  |  |  |  |  |  |  |  |  |
| 19 | 50 | 2 | 0.01 | 10 | 7 | ✓ | ✓ | ✓ |  |  |  |  |  |  |  |  |  |  |  |
| 20 | 50 | 2 | 0.01 | 20 | 7 | ✓ | ✓ | ✓ |  |  |  |  |  |  |  |  |  |  |  |
| 21 | 50 | 2 | 0.01 | 25 | 7 | ✓ | ✓ | ✓ |  |  |  |  |  |  |  |  |  |  |  |
| 22 | 50 | 2 | 0.01 | 35 | 7 | ✓ | ✓ | ✓ |  |  |  |  |  |  |  |  |  |  |  |
| 23 | 50 | 2 | 0.01 | 40 | 7 | ✓ | ✓ | ✓ |  |  |  |  |  |  |  |  |  |  |  |
| 24 | 50 | 2 | 0.01 | 50 | 7 | ✓ | ✓ | ✓ |  |  |  |  |  |  |  |  |  |  |  |
| Number of trees that featured the variable: | | | | | | 23 | 22 | 20 | 3 | 2 | 1 | 1 | 0 | 0 | 0 | 0 | 0 | 0 | 0 |


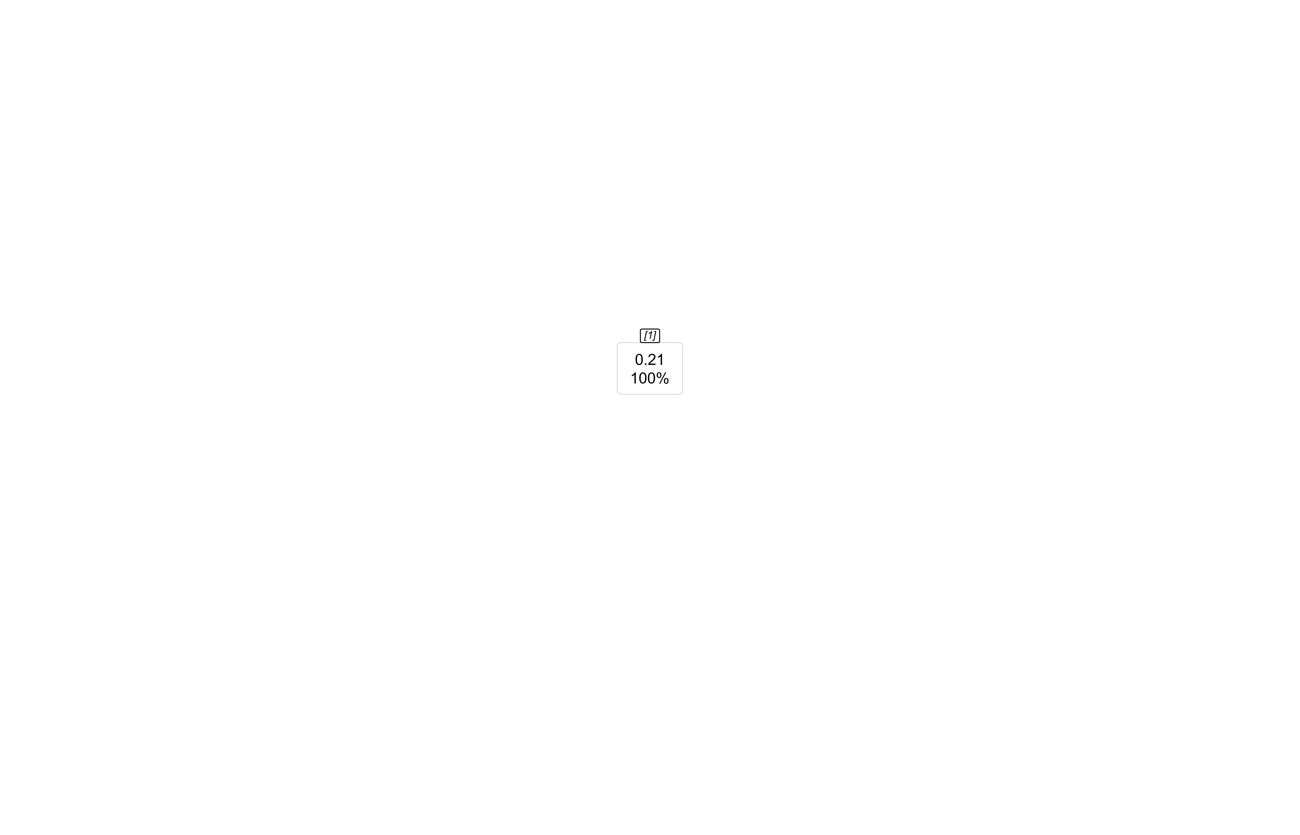


**Figure S97 – Test 7’s decision tree^[[1]](#footnote-1)^**


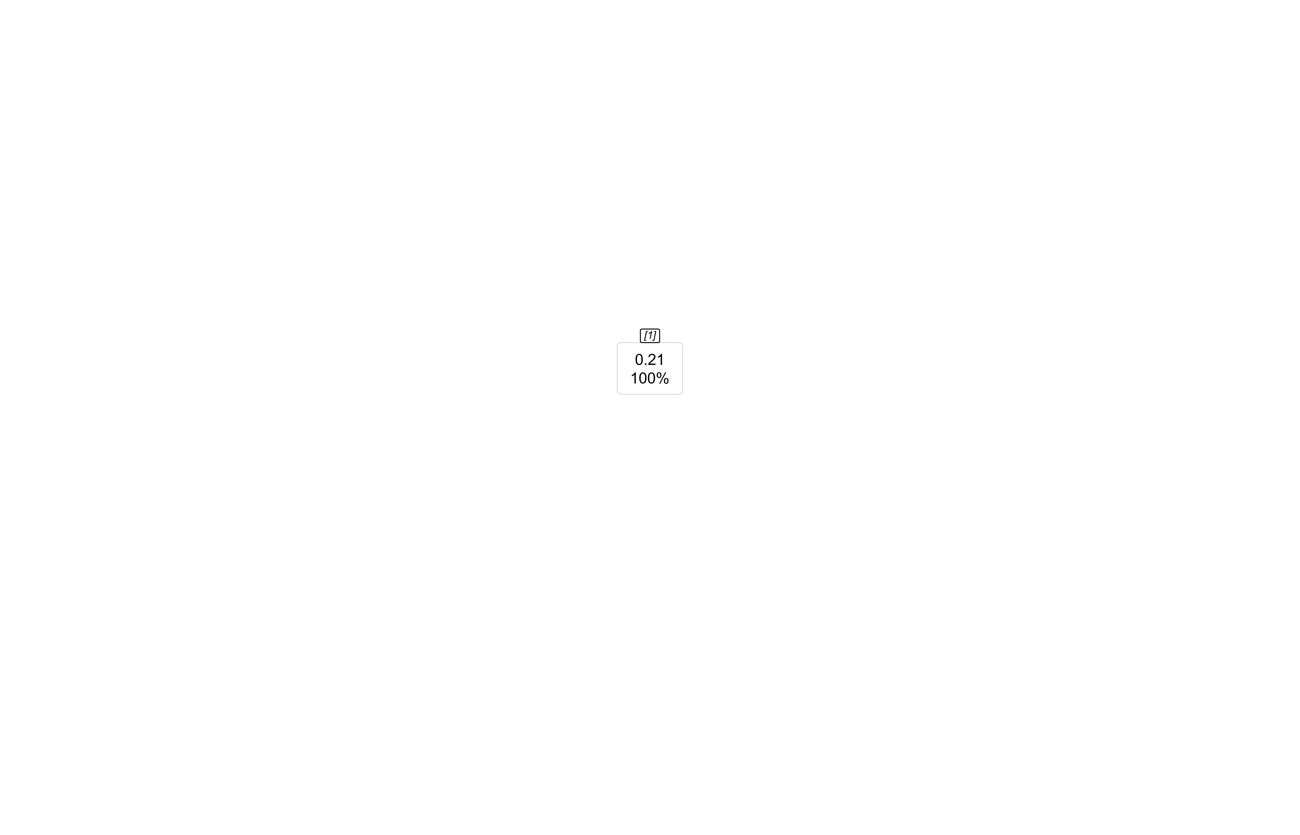


**Figure S98 – Test 8’s decision tree**


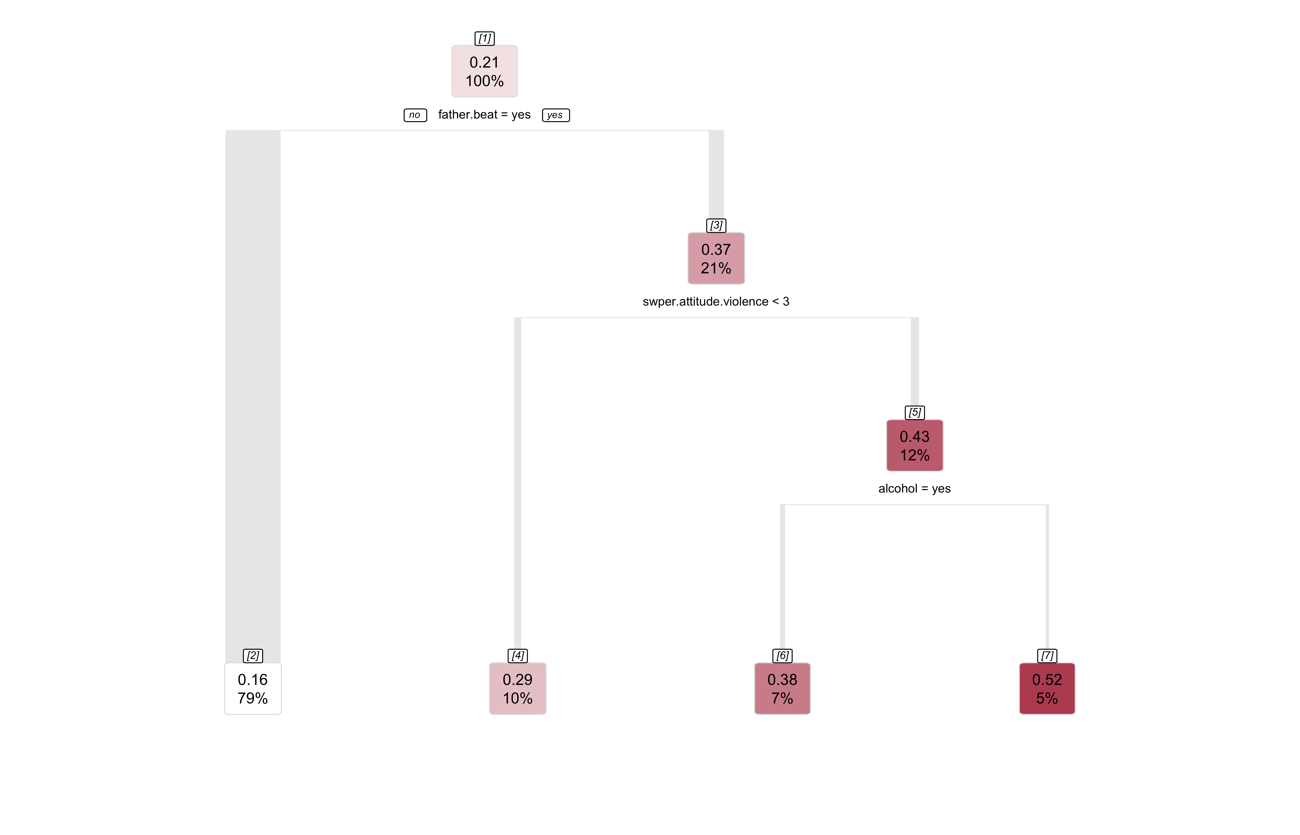


**Figure S99 – Test 9’s decision tree^[[2]](#footnote-2)^**


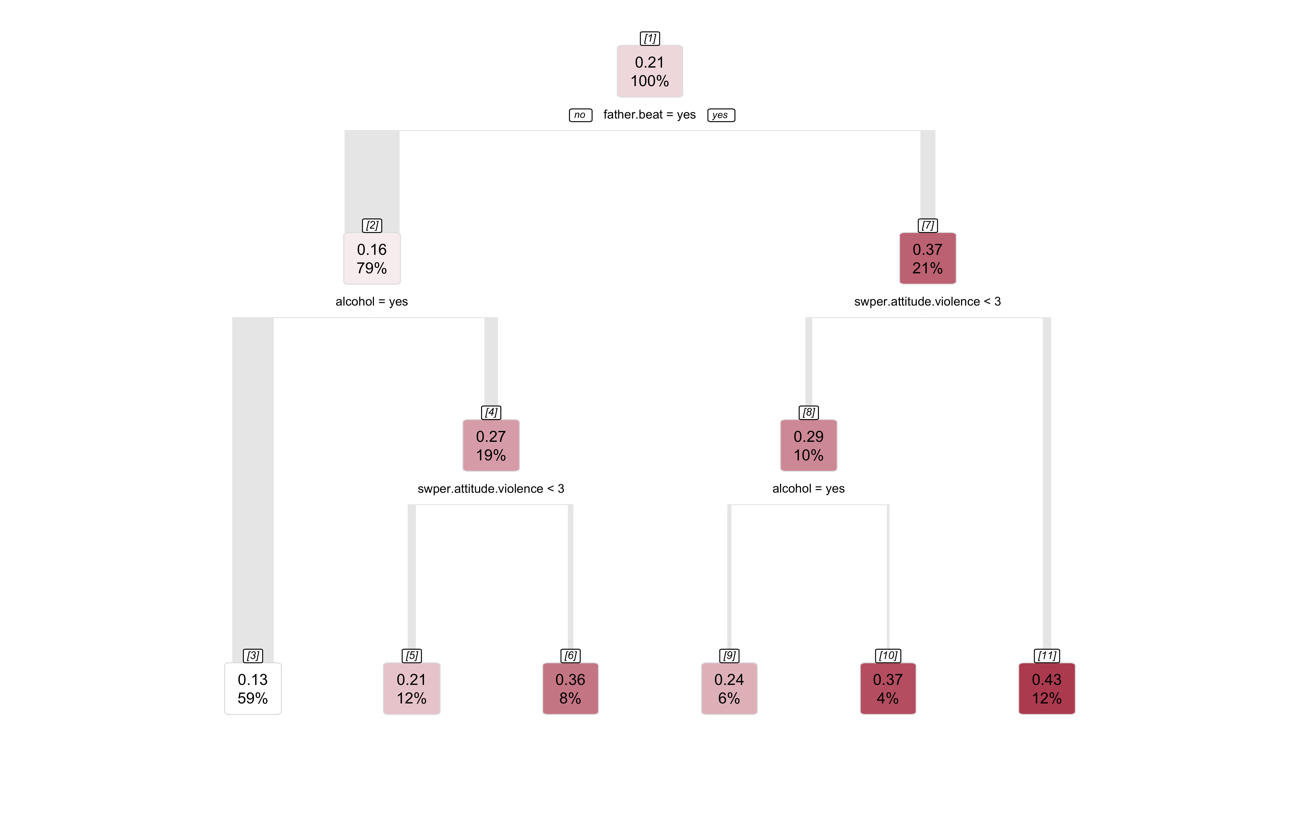


**Figure S100 – Test 10’s decision tree**

**Figure S101 – Test 11’s decision tree**

**Figure S102 – Test 12’s decision tree^[[3]](#footnote-3)^**

**Figure S103 – Test 13’s decision tree^[[4]](#footnote-4)^**

**Figure S104 – Test 14’s decision tree**

**Figure S105 – Test 15’s decision tree**

**Figure S106 – Test 16’s decision tree**

**Figure S107 – Test 17’s decision tree**

**Figure S108 – Test 18’s decision tree**

1. In each node, there is a fraction and a percentage. The fraction is the IPV prevalence (21%) and the percentage represents the percentage of all women in the sample that were assigned to this node (100%). [↑](#footnote-ref-1)
2. father.beat = Witnessed father-to-mother IPV in childhood; swper.attitude.violence = Woman’s empowerment – attitude to violence; alcohol = Partner’s alcohol use [↑](#footnote-ref-2)
3. swper.social.independence = Woman’s empowerment – social independence [↑](#footnote-ref-3)
4. swper.decision.making = Woman’s empowerment – decision making; wiq = Wealth index quintiles; [↑](#footnote-ref-4)
